# Colonic *Engyodontium* fungus triggers neutrophil antimicrobial activity to suppress *Lactobacillus johnsonii*–derived glutamic acid–maintained Tregs

**DOI:** 10.1172/JCI196788

**Published:** 2026-02-17

**Authors:** Xinying Wang, Haiyang Sun, Ying Tan, Shaoting Xu, Zishan Liu, Kaile Ji, Ding Qiu, Jianping Deng, Bingbing Feng, Xueting Wu, Yoichiro Iwakura, Minhu Chen, Rui Feng, Chanyan Huang, Ce Tang

**Affiliations:** 1 Department of Gastroenterology and Hepatology, and; 2 Institute of Precision Medicine, The First Affiliated Hospital, Sun Yat-sen University, Guangzhou, Guangdong Province, China.; 3 College of Basic Medical Science, Dalian Medical University, Dalian, China.; 4 Center for Animal Disease Models, Research Institute for Biomedical Sciences, Tokyo University of Science, Noda, Chiba, Japan.; 5 Department of Anesthesiology, and; 6 Animal Experiment Center, The First Affiliated Hospital, Sun Yat-sen University, Guangzhou, Guangdong Province, China.

**Keywords:** Gastroenterology, Microbiology, Innate immunity, Microbiome

## Abstract

Isolating commensal fungi from mouse intestines has been challenging, limiting our understanding of their role in intestinal immune homeostasis and diseases. Using an Fc fusion protein of the C-type lectin receptor Dectin-2, we successfully purified the commensal Ascomycota fungus *Engyodontium* sp. from mouse feces. *Engyodontium* enhances the antimicrobial activity of colonic neutrophils via the CARD9 pathway and exacerbates colitis by impairing the colonization of intestinal *Lactobacillus johnsonii* WXY strain. *L*. *johnsonii* produces high levels of l-glutamic acid by expressing the glutaminase-encoding gene *glsA* to facilitate Treg expansion via enhancing IL-2 receptor signaling. Patients with Crohn disease (CD) and ulcerative colitis harbored increased *Engyodontium* and decreased *L*. *johnsonii* abundance. *Engyodontium* directly induced calprotectin in human colonic neutrophils, and patients with CD had lower levels of l-glutamic acid, which also promoted human Treg expansion. These findings highlight the *Engyodontium*-calprotectin axis against the *Lactobacillus*-glutamate-Treg cascade to aggravate colitis, suggesting commensal *Engyodontium*-triggered signaling as a therapeutic target for mucosal inflammatory diseases.

## Abstract 2

Gut fungus Engyodontium exacerbates colitis by reducing calprotectin-sensative commensal Lactobacillus that support regulatory T cells, revealing a new target for treating inflammatory bowel diseases.

## Graphical abstract

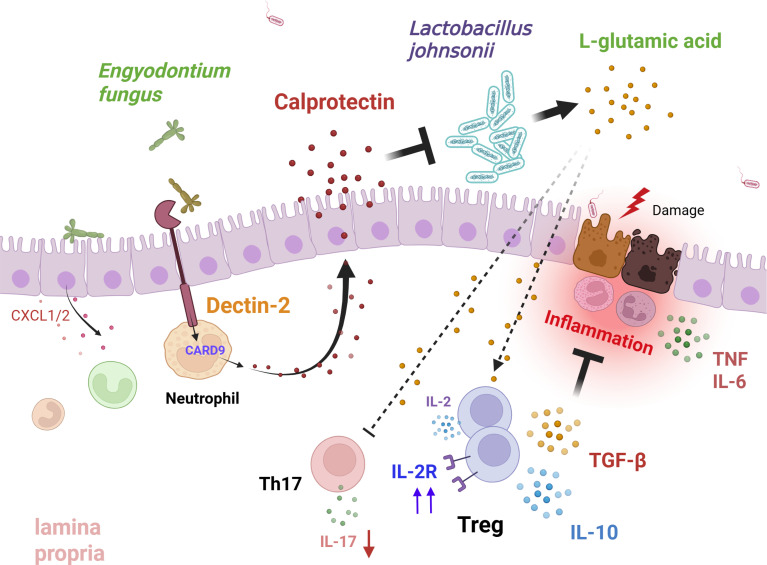

## Introduction

Commensal fungi are increasingly recognized as components of the gut microbiota, yet their roles in intestinal homeostasis and disease remain poorly understood (1). Although more than 2,700 prokaryotic species have been isolated from humans (2), only approximately 200 fungal species have been identified in feces (3), and very few have been cultured from mouse intestines (4, 5). Many fungi detected by sequencing remain unculturable, highlighting the need for new approaches to isolate live gut fungi and define their functions.

Inflammatory bowel disease (IBD), including Crohn disease (CD) and ulcerative colitis (UC), is driven by complex interactions among genetic, microbial, environmental, and immune factors (6–8). Fungi such as *Candida albicans* have been implicated in IBD (3, 9); however, *C*. *albicans* exacerbates dextran sulphate sodium–induced (DSS-induced) colitis only in immunocompromised mice and does not induce disease in immunocompetent hosts or after long-term colonization (9, 10). Despite frequent detection of *Candida* species by internal transcribed spacer (ITS) sequencing (4), viable strains have not been isolated from mouse intestines, to our knowledge.

Innate and adaptive immunity both contribute to IBD, with pattern recognition receptors, including NOD-like receptors, Toll-like receptors (TLRs), and C-type lectin receptors (CLRs), maintaining intestinal immune homeostasis (11–13). The CLR Dectin-1 recognizes β-glucan and limits pathogenic *Candida* invasion (4). Dectin-2 (gene *Clec4n* in mice, *CLEC6A* in humans), another CLR member also expressed on myeloid-derived cells, recognizes α-mannan and other mannosyl glycans (14–16), and activates SYK/CARD9/NF-κB signaling to induce IL-1, IL-23, and Th17 responses (15, 17, 18). However, its role in sensing commensal fungi in the gut remains unclear.

Specific commensal microbes shape intestinal immunity through antigens and metabolites. Segmented filamentous bacteria induce Th17 cells (19). *Bacteroides fragilis* drives Treg cell development via polysaccharides (20), and *Clostridium* species induce Treg differentiation via butyrate (21, 22). In contrast, *Fusobacterium nucleatum* enhances Th17 infiltration via aryl hydrocarbon receptor activation (23), underscoring the diverse immunomodulatory effects of microbial metabolites.

Here, we isolated a live Ascomycota fungal species from mouse feces using Dectin-1 and -2 fused to an Ab Fc fragment (Dectin-1/2-Fc). We show that this fungus activates colonic inflammation through Dectin-2/CARD9 signaling, enhances neutrophil antimicrobial responses, suppresses Treg populations, and exacerbates colitis. Moreover, it antagonizes a *Lactobacillus* species that supports Treg differentiation. These findings identify a commensal fungus–Dectin-2 axis that contributes to IBD pathogenesis and represents a potential therapeutic target.

## Results

### Intestinal commensal Engyodontium fungus modulates mucosal immunity and reshapes the commensal microbiota.

**Figure 1:**
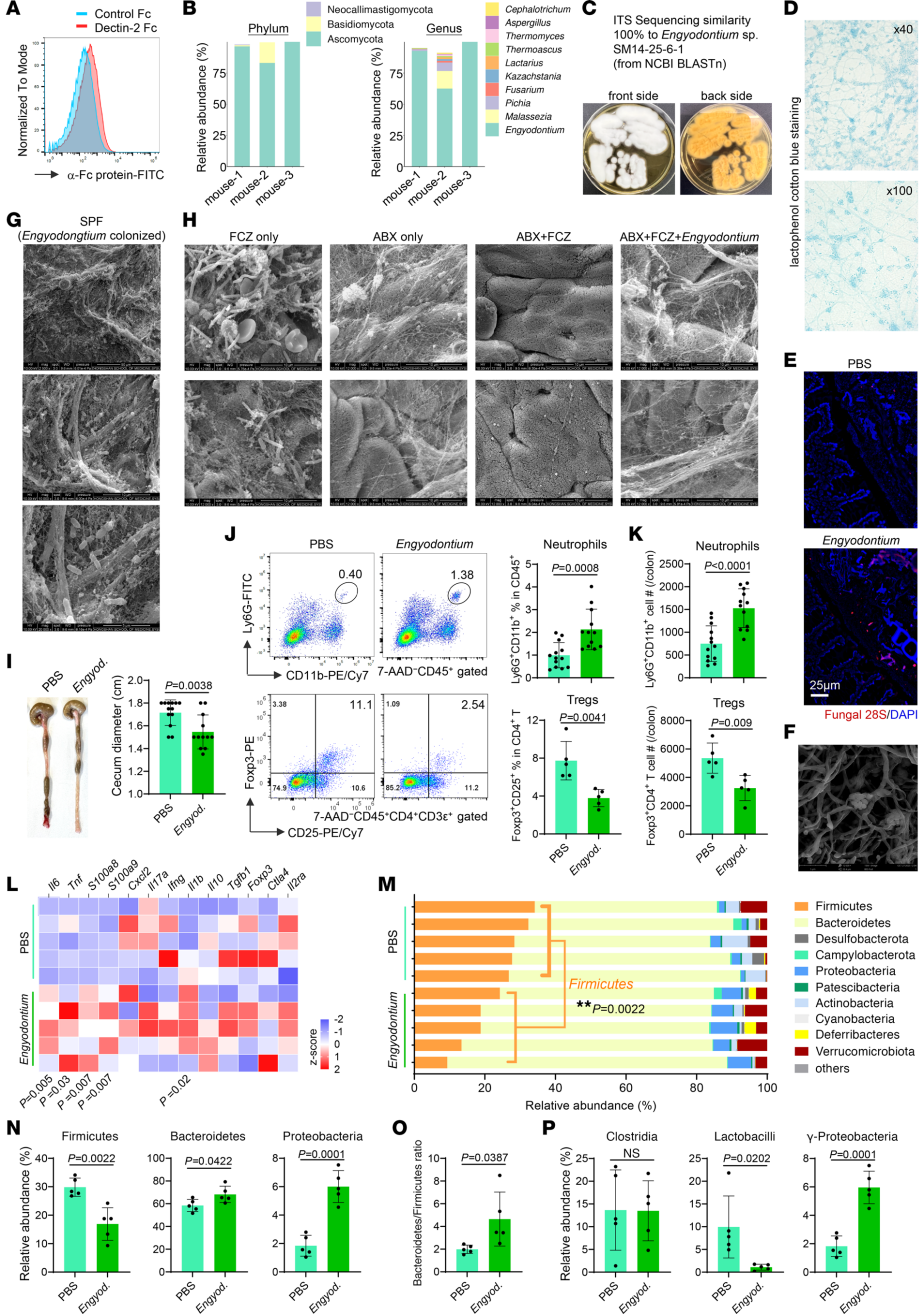
*Engyodontium* sp. colonization modifies intestinal immune profiles and commensal microbiota in steady-state mice. (**A**) Flow cytometric analysis of Dectin-2-Fc binding to mouse fecal commensal microorganisms. (**B**) Phylum- and genus-level composition of mouse fecal commensal fungi isolated using Dectin-2-Fc and identified by ITS1–2 sequencing. (**C**) Representative images of purified *Engyodontium* sp. colonies cultured on antibiotic-containing medium (kanamycin, gentamicin, colistin, metronidazole, vancomycin, and erythromycin) at room temperature for 4 days. Left, top view of culture plate; right, bottom view of culture plate. (**D**) Light microscopy images of *Engyodontium* sp. cultured on PDA plates for 48 hours and stained with lactophenol cotton blue. (**E**) C57BL/6J SPF mice were treated with fluconazole for 1 week and orally administered *Engyodontium* sp. every other day for 3 doses. Mice were euthanized 7 days after the final administration, and fungal colonization was assessed by FISH using probes specific for fungal 28S rDNA on colon tissue sections. (**F**) Scanning electron microscopy (SEM) images of hyphae and conidia of *Engyodontium* sp. cultured on PDA plates for 2 days. (**G** and **H**) SPF mice were pretreated with combined antibiotics and/or fluconazole for 1 week, followed by *Engyodontium* colonization every other day for 3 doses. Colons were harvested 13 days (**G**) or 8 days (**H**) after the initial colonization and analyzed by SEM to visualize intestinal microorganisms. (**I**–**P**) SPF mice were colonized with *Engyodontium* sp. every other day for 3 doses and euthanized 13 days later. (**I**) Representative images of the cecum and colon. (**J**) Representative flow cytometry plots showing frequencies of Ly6G^+^CD11b^+^ neutrophils and Foxp3^+^CD25^+^ Treg cells in cLP. (**K**) Absolute numbers of cLP neutrophils and Treg cells quantified by flow cytometry (**I**–**K**: PBS *n* = 13, *Engyodontium* *n* = 12). (**L**) Heatmap depicting mRNA expression of immune-related genes in colon tissues by qPCR. (**M**–**P**) Fecal bacterial microbiota analyzed by 16S rDNA-Seq, shown as stacked bar plots at the phylum level (**M**), relative abundance of indicated bacterial phyla (**N**), Bacteroidetes/Firmicutes ratio (**O**), and relative abundance of indicated bacterial orders (**P**) (**L**–**P**: *n* = 5/group). Data in **I**–**K** are pooled from 3 independent experiments. Data in **L** and **M** are from 1 of 2 independent experiments. Data in **I**–**K** and **N**–**P** are presented as mean ± SD. Statistical analysis: 2-tailed unpaired Student’s *t* test (**I**–**P**). *Engyod*., *Engyodontium;* ABX, antibiotics; FCZ, Fluconazole.

To isolate live intestinal fungi, we generated mouse Dectin-1-Fc and Dectin-2-Fc fusion proteins, both of which bound fecal commensal microorganisms (Figure 1A and Supplemental Figure 1A; supplemental material available online with this article; https://doi.org/10.1172/JCI196788DS1). Magnetic purification followed by ITS sequencing revealed Ascomycota as the dominant phylum, with *Engyodontium* as the predominant genus (Figure 1B and Supplemental Figure 1, B and C). Although *Engyodontium* species have been reported as opportunistic pathogens in humans (24), they have not previously been identified as murine commensals, to our knowledge.

CLR-bound microorganisms cultured on potato dextrose agar (PDA) medium yielded white hyphal colonies, and ITS sequencing confirmed 100% identity to *Engyodontium* sp. SM14-25-6-1 (hereafter, *Engyodontium* sp.) (Figure 1C and Supplemental Figure 1D). Isolation from Dectin-1–bound fractions was unsuccessful, likely due to low fungal abundance and bacterial contamination. Lactophenol cotton blue staining revealed hyphae with conidia (Figure 1D). FISH targeting fungal 28S rDNA demonstrated fungal localization in the colonic lumen and epithelial surface of colonized mice (Figure 1E). Scanning electron microscopy confirmed hyphae and conidia in culture (Figure 1F) and hyphal structures associated with epithelial surfaces and net-like extracellular structures in vivo (Figure 1, G and H).

Two-week oral colonization of specific-pathogen-free (SPF) mice with *Engyodontium* sp. did not alter colon length but slightly reduced cecal size (Figure 1I and Supplemental Figure 1E). Flow cytometry revealed increased CD11b^+^Ly6G^+^ neutrophils and reduced CD25^+^Foxp3^+^ Treg cells in the colonic lamina propria (cLP) (Figure 1, J and K), with increased IL-17^+^CD4^+^ T cells but no changes in other immune populations (Supplemental Figure 1, F and G). Colonic expression of *Il6*, *Tnf*, *S100a8*, and *S100a9*, as well as protein levels of IL-6, TNF-α, IL-1β, and calprotectin, were significantly increased (Figure 1L and Supplemental Figure 1, H and I).

Microbiota profiling revealed marked dysbiosis, characterized by reduced Firmicutes, increased Proteobacteria, and an altered Bacteroidetes/Firmicutes ratio (Figure 1, M–O). Within Firmicutes, the species *Lactobacillus* was selectively depleted, whereas γ-Proteobacteria were enriched (Figure 1P), indicating that *Engyodontium* selectively reshapes bacterial communities.

### Engyodontium induces epithelial chemokines to recruit neutrophils and exacerbates colitis.

**Figure 2:**
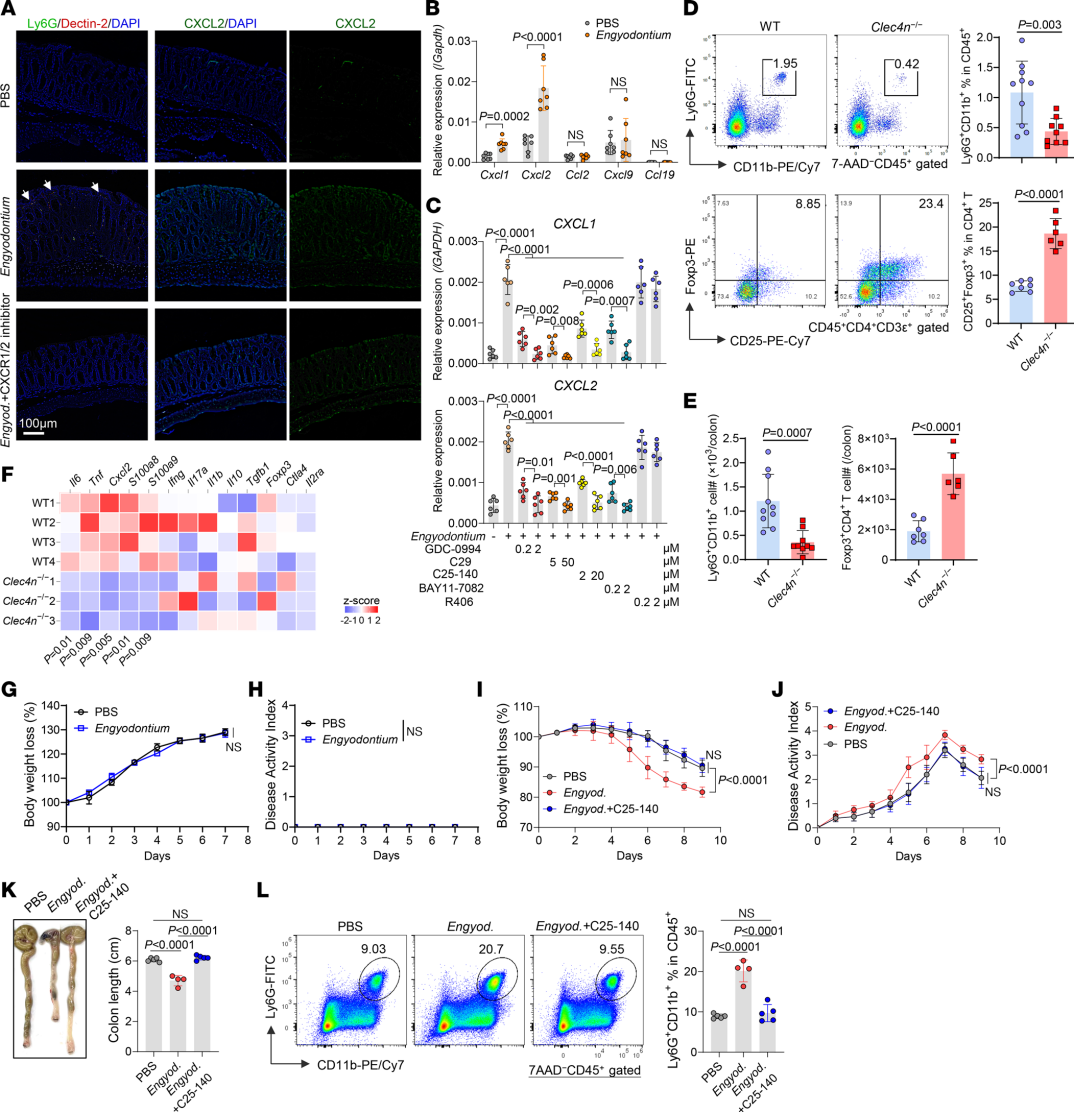
*Engyodontium* induces epithelial chemokine production to recruit neutrophils and exacerbates colitis. (**A** and **B**) C57BL/6J mice were orally colonized with *Engyodontium* sp. every other day for 3 doses and simultaneously treated intraperitoneally with the CXCR1/2 inhibitor reparixin (15 mg/kg) for 3 doses. On day 8 after the initial colonization, colons were harvested. (**A**) Immunofluorescent staining of Ly6G, Dectin-2, and CXCL2 on serial colon tissue sections. (**B**) Colonic epithelial cells were isolated after EDTA treatment, and chemokine mRNA expression was quantified by qPCR (*n* = 7 technical replicates/group). (**C**) NCM460 epithelial cells were cocultured with *Engyodontium* sp. for 12 hours in the presence of inhibitors targeting ERK (GDC-0994), NF-κB (BAY11-7082), TLR2 (C29), TRAF6 (C25-140), or SYK (R406). *CXCL1* and *CXCL2* mRNA expression was measured by qPCR (*n* = 6 technical replicates/group). (**D**–**F**) WT and *Clec4n*^–/–^ mice were analyzed under physiological conditions. (**D** and **E**) Frequencies (**D**) and absolute numbers (**E**) of cLP neutrophils (WT *n* = 10, *Clec4n*^–/–^ *n* = 9) and Tregs (WT *n* = 7, *Clec4n*^–/–^ *n* = 6) measured by flow cytometry. (**F**) Heatmap showing qPCR analysis of cytokine and antimicrobial gene expression in colon tissues (WT *n* = 4, *Clec4n*^–/–^ *n* = 3). (**G** and **H**) Body weight change (**G**) and colitis DAI (**H**) after *Engyodontium* colonization. (**I**–**L**) Fluconazole-pretreated mice were colonized with *Engyodontium* sp. and treated with 1.5% DSS for 7 days starting from the third fungal administration. TRAF6 inhibitor C25-140 was administered intraperitoneally every other day for 4 doses. (**I**) Body weight loss, (**J**) DAI, (**K**) gross colon morphology and length, and (**L**) cLP neutrophil frequencies assessed by flow cytometry (PBS *n* = 5, *Engyodontium* *n* = 4; *Engyodontium*-C25-140 *n* = 5). Data in **B**–**E** (Treg panel) are pooled from 2 independent experiments. Data in **D** and **E** (neutrophil panel) are pooled from 3 independent experiments. Data in **F**–**L** are from 1 of 2 independent experiments. Data in **B**–**E** and **G**–**L** are presented as mean ± SD. Statistical analysis: 2-tailed unpaired Student’s *t* test (**B**, and **D**–**H**), 1-way ANOVA with Bonferroni’s multiple-comparison test (**I** and **J**), and 1-way ANOVA with Tukey’s multiple-comparison test (**C**, **K**, and **L**). *Engyod.*, *Engyodontium*.

*Engyodontium* colonization selectively increased colonic Dectin-2 (*Clec4n*) expression without affecting Dectin-1 or other CLRs (Supplemental Figure 2A). Colonized mice showed marked accumulation of Ly6G^+^ neutrophils in the lamina propria and epithelium-adjacent regions (Figure 2A), accompanied by robust CXCL2 expression in epithelial and goblet cells. Dectin-2 colocalized predominantly with recruited neutrophils, and CXCR1/2 blockade reduced neutrophil infiltration without altering epithelial CXCL2 expression (Figure 2A).

qPCR analysis demonstrated selective induction of epithelial *Cxcl1* and *Cxcl2*, whereas *Ccl2*, *Cxcl9*, and *Ccl19* remained unchanged (Figure 2B). In vitro, *Engyodontium* directly induced CXCL1/2 in human NCM460 epithelial cells via TLR2/TRAF6/NF-κB and ERK signaling, independently of SYK (Figure 2C), consistent with epithelial cell–driven neutrophil recruitment (25).

ITS sequencing revealed comparable fungal diversity and abundance between WT and Dectin-2–deficient (*Clec4n*^–/–^) mice, with only minor enrichment of Tremellales in *Clec4n*^–/–^ mice (Supplemental Figure 2, B–F), indicating that Dectin-2 does not control fungal colonization. Single-cell RNA-Seq (scRNA-Seq) analysis showed *Clec4n* expression primarily in neutrophils, correlating with neutrophil markers *G0S2* and *Cxcr2* (Supplemental Figure 2, G and H).

At steady state, *Clec4n*^–/–^ mice exhibited normal colon morphology and epithelial integrity (Supplemental Figure 2, I and J) and normal populations of antigen-presenting myeloid cells and T cells (Supplemental Figure 2, K–M), but had fewer colonic neutrophils and expanded Treg populations (Figure 2, D and E), with decreased expression of *Il6*, *Tnf*, *Cxcl2*, *S100a8*, and *S100a9* (Figure 2F and Supplemental Figure 2N). Neutrophils were the major source of S100A8/S100A9 in the colon (Supplemental Figure 2O).

Although *Engyodontium* alone did not induce colitis (Figure 2, G and H), its colonization markedly exacerbated DSS-induced weight loss, diarrhea, bleeding, and colon shortening (Figure 2, I and J). Pharmacological inhibition of TRAF6 abolished *Engyodontium*-driven neutrophil accumulation and colitis exacerbation (Figure 2, I–L, and Supplemental Figure 2P), indicating that *Engyodontium* exacerbates intestinal inflammation first through recruiting neutrophils.

### Engyodontium promotes colitis via Dectin-2/CARD9 signaling.

**Figure 3:**
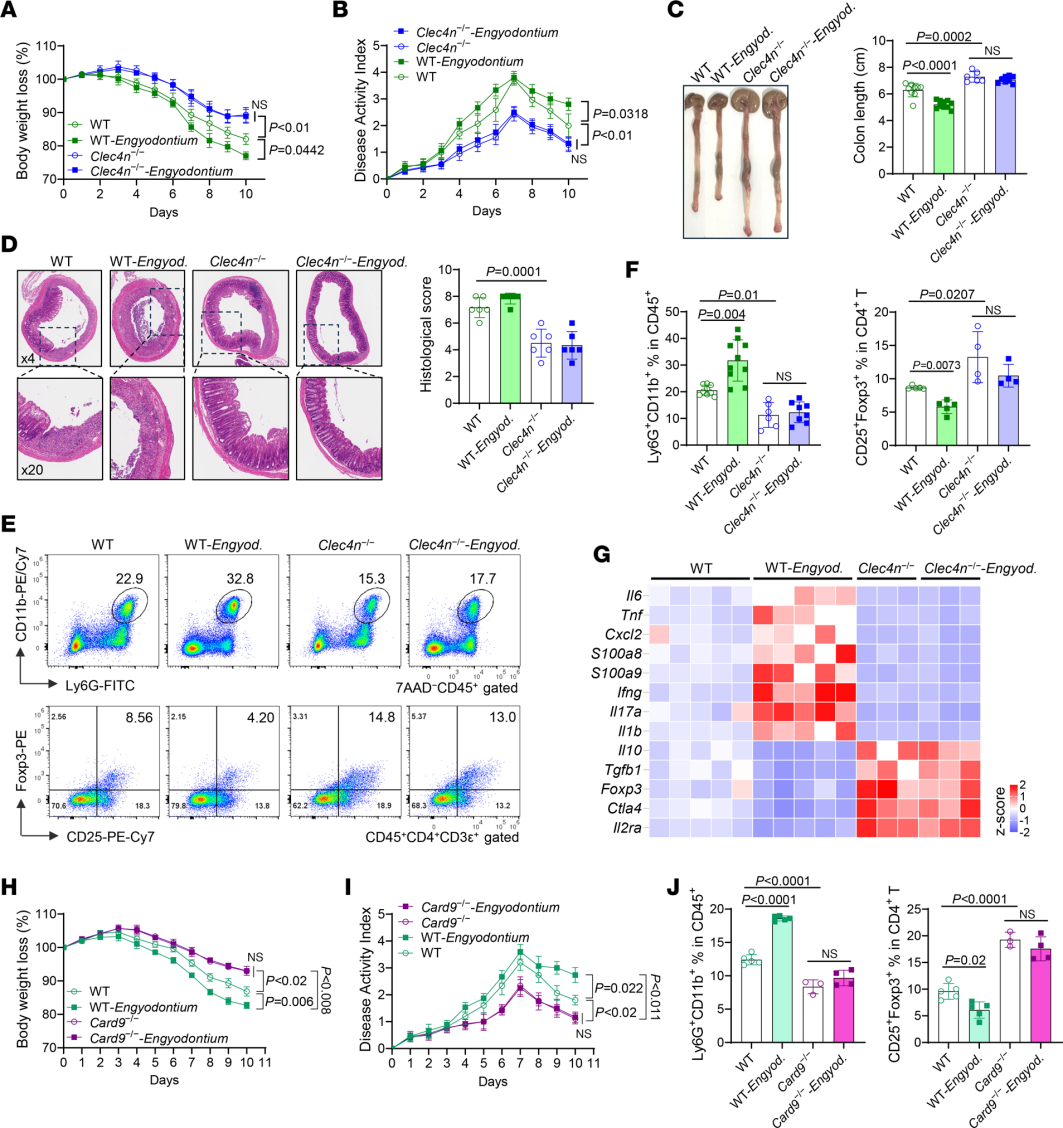
*Engyodontium* exacerbates mouse colitis through the Dectin-2/CARD9 pathway. (**A**–**G**) WT and *Clec4n*^–/–^ mice were orally administered PBS or *Engyodontium* sp. every other day for 3 doses and treated with 1.5% DSS for 7 days starting from the second fungal administration. (**A**) Body weight loss and (**B**) DAI were monitored daily. (**C**) Gross colon morphology and length measurements at sacrifice on day 10. (**D**) Representative H&E-stained distal colon sections. (**E** and **F**) Frequencies of cLP neutrophils and Treg cells measured by flow cytometry. (**G**) Heatmap of immune-related gene expression in colon tissues assessed by qPCR. Neutrophil panel (A–C and F, left): WT *n* = 9, WT-*Engyodontium* *n* = 10, *Clec4n*^–/–^ *n* = 6, *Clec4n*^–/–^-*Engyodontium* *n* = 8; (**D**) *n* = 6/group; (**F**, Treg panel) WT *n* = 5, other groups *n* = 4; (**G**) WT groups *n* = 5, *Clec4n*^–/–^ groups *n* = 3. (**H**–**J**) WT and *Card9*^–/–^ mice were subjected to the same *Engyodontium* colonization and DSS treatment protocol. Body weight loss (**H**), DAI (**I**), and frequencies of cLP neutrophils and Treg cells (**J**) determined by flow cytometry (WT groups *n* = 5; *Card9*^–/–^ *n* = 3; *Card9*^−/−^-*Engyodontium* *n* = 4). Data in **A**–**D** and **F** (neutrophil panel) are pooled from 2 independent experiments. Data in **E** and **F** (Treg panel) and **G**–**J** are from 1 of 2 independent experiments. Data in **A**–**D**, **F**, and **H**–**J** are presented as mean ± SD. Statistical analysis: 1-way ANOVA with Bonferroni’s multiple-comparison test (**A**, **B**, **H**, and **I**), or 1-way ANOVA with Tukey’s multiple-comparison test (**C**, **D**, **F**, and **J**). *Engyod.*, *Engyodontium*.

To define the role of Dectin-2, *Engyodontium* was transferred into *Clec4n^–/–^* mice prior to DSS treatment. Unlike WT mice, *Clec4n^–/–^* mice were protected from *Engyodontium*-mediated disease exacerbation (Figure 3, A and B) and had reduced histological inflammation and immune infiltration (Figure 3, C and D). *Engyodontium* increased neutrophils, reduced Tregs, and expanded Th17 cells only in WT mice (Figure 3, E and F, Supplemental Figure 3A). Proinflammatory cytokines were elevated in WT but suppressed in *Clec4n^–/–^* mice, whereas *Il10*, *Tgfb1*, and *Foxp3* were selectively increased in *Clec4n^–/–^* colons (Figure 3G and Supplemental Figure 3B).

Consistent with Dectin-2 signaling via SYK/CARD9 (15, 17), *Card9^–/–^* mice phenocopied *Clec4n^–/–^* mice, displaying attenuated DSS colitis and resistance to *Engyodontium*-mediated exacerbation (Figure 3, H and I, and Supplemental Figure 3, C and D). *Card9^–/–^* mice had fewer neutrophils, increased Tregs, suppressed inflammatory cytokines, and elevated mRNA expression of *Il10*, *Tgfb1*, *Foxp3*, and *Ctla4* irrespective of fungal colonization (Figure 3J and Supplemental Figure 3, E and F).

Although Dectin-1 was also expressed on neutrophils (Supplemental Figure 2G), *Clec7a^–/–^* mice still developed *Engyodontium*-exacerbated colitis with preserved neutrophil accumulation (Supplemental Figure 3, G–J). Gene expression analyses indicated that Dectin-1 selectively regulated *S100a8* but was dispensable for Dectin-2–dependent inflammatory amplification (Supplemental Figure 3K).

Together, these data demonstrate that *Engyodontium* exacerbates colitis primarily through Dectin-2/CARD9 signaling, driving neutrophil recruitment, calprotectin production, Treg suppression, and inflammatory amplification, whereas Dectin-1 plays a limited role.

### Elimination of intestinal commensal fungi suppresses Dectin-2/CARD9–dependent colitis development and calprotectin expression.

*Engyodontium* grows optimally at approximately 25°C; therefore, we examined its persistence in the mouse gut. Following fluconazole-mediated fungal depletion, mice received 3 oral doses of *Engyodontium* (5 × 10^8^ CFU) (Supplemental Figure 5A). qPCR analysis showed rapid decline after initial colonization, stabilizing by day 28 (~0.03 relative to murine *Gapdh*) and persisting for at least 4 weeks, with 10 times higher than the level in mice at steady state (Supplemental Figure 4, A and B). Despite suboptimal temperature, *Engyodontium* achieved stable long-term colonization, accounting for approximately 85%–95% of intestinal Ascomycota and displacing endogenous fungi (Supplemental Figure 4C).

**Figure 4:**
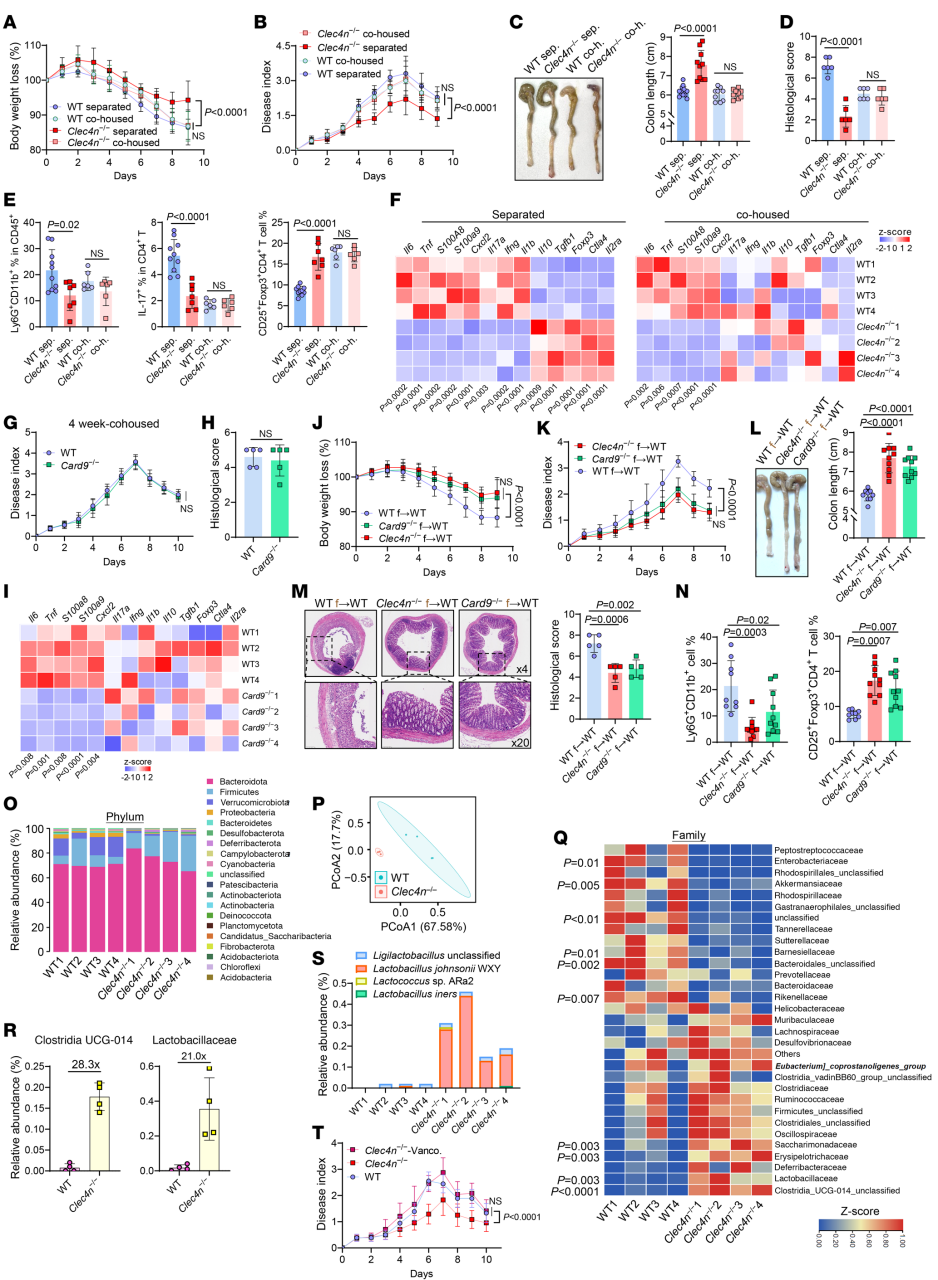
Dectin-2/calprotectin axis restricts intestinal colonization of *L.j.* WXY, which suppresses colitis via Treg-promoting metabolite. (**A**) Fecal microbiota from *Clec4n*^–/–^ mice were cultured on MRS medium supplemented with neomycin for 3 days. Single colonies were isolated and identified by PCR amplification and sequencing of bacterial 16S rDNA. (**B**–**F**) *L.j*. WXY strain was isolated from mouse feces. WT mice were treated with antibiotics (ABX), followed by oral transfer of the indicated bacterial strains twice over 7 days. Whole fecal microbiota from normal WT mice were then transferred back for 3 days, followed by DSS treatment for 7 days and sacrifice on day 10. Body weight loss (**B**), DAI (**C**), colon length measurement (**D**), distal colon histology (**E**), and cLP neutrophil and Treg frequencies (**F**) (**B**–**D**, and **F**, PBS, *n* = 7; *L*.*j*. WXY, *n* = 7; *Lactobacillus* mixture, *n* = 8; *E*. *coli*, *n* = 7; **E**, *n* = 3 /group). (**G**) Correlation between fecal *Lactobacillus* abundance and colonic *S100a8*/*S100a9* mRNA expression in C57BL/6J mice under steady-state conditions (*n* = 18). (**H**) *L.j*. WXY was cultured anaerobically at 37°C with recombinant S100A8 + S100A9 (1:1 mixture, 5 μg/mL each). Bacterial growth was quantified spectrophotometrically at 6 and 24 hours (*n* = 6 technical replicates/group). (**I**) *Clec4n*^–/–^ mice received intrarectal administration of recombinant S100A8 + S100A9 for 5 hours, followed by quantification of fecal *L.j*. abundance by qPCR (*n* = 10). (**J** and **K**) WT and *Clec4n*^–/–^ recipient mice were lethally irradiated and reconstituted with BM cells from WT or *Clec4n*^–/–^ donors. After 30 days, colonic expression of *S100a8* and *S100a9* was assessed by qPCR (**J**), and fecal *Lactobacillus* abundance was measured by qPCR on days 10, 20, and 30 after transfer (**K**) (WT→WT, *n* = 8; WT→*Clec4n*^–/–^, *n* = 7; *Clec4n*^–/–^→WT, *n* = 8; *Clec4n*^–/–^→*Clec4n*^–/–^, *n* = 8.). (**L**–**N**) WT and *Clec4n*^–/–^ mice were treated intraperitoneally with anti-Ly6G neutralizing Ab or isotype control IgG (100 μg/mouse) every other day for 5 doses. Two days after the final injection, colonic tissues and feces were collected. S100A8 and S100A9 protein levels in colon lysates were measured by ELISA (**L**), fecal *L.j*. abundance was determined by qPCR (**M**), and colonic *Il6* and *Tnf* expression was assessed by qPCR (**N**) (control [con] IgG, *n* = 3–4; anti-Ly6G, *n* = 3–4). (**O**–**S**) WT mice were orally administered culture supernatant (sup.) from *L.j*. WXY or heat-killed (hk) bacteria daily for 3 days before and throughout 7 days of DSS treatment (*n* = 10 total administrations). Body weight loss (**O**), DAI (**P**), colon length measurement at sacrifice on day 9 (**Q**), distal colon histology (**R**), and cLP Treg frequencies (**S**) (PBS *n* = 8; WXY-sup. *n* = 8; WXY-hk *n* = 9). (**T**) CD11b^+^ and CD11c^+^ cells isolated from WT cLP were stimulated in vitro with *L.j*. WXY, *Lactobacillus* mixture, or *E*. *coli*. After 12 hours, *Il10* and *Tgfb1* mRNA expression was quantified by qPCR (*n* = 4 technical replicates/group). (**U**) CD62L^+^ naive CD4^+^ T cells isolated from WT spleen and lymph nodes were polarized toward Treg differentiation in the presence of *L.j*. WXY culture supernatant. After 6 days, *Il10* and *Tgfb1* expression was measured by qPCR (*n* = 9 technical replicates from 3 biological replicates/group). (**V** and **W**) ABX-treated WT mice received oral *L.j*. WXY or PBS, followed by fecal microbiota transplantation from normal WT mice and subsequent DSS treatment. Cecal contents were collected on day 10 for untargeted metabolomic analysis by liquid chromatography. Histogram (**V**) shows the top 20 enriched metabolites, and volcano plot (**W**) highlights significantly altered metabolites in WXY-treated mice. (**X**) Targeted metabolomic analysis of l-glutamic acid levels in cecal contents from 8-week-old WT and *Clec4n*^–/–^ mice under physiological conditions (*n* = 3/group). (**Y**) l-Glutamic acid concentrations in culture supernatants of *L.j*. WXY, *Lactobacillus* mixture, or *E*. *coli* measured by targeted metabolomics (*n* = 3 replicates/group). Data in **B**–**F**, **H**–**K**, and **O**–**S** are pooled from 2 independent experiments. Data in **B**–**F**, **J**, **L**–**U**, **X**, and **Y** are presented as mean ± SD. Statistical analysis: 1-way ANOVA with Bonferroni’s multiple-comparison test (**B**, **C**, **K**, **O**, and **P**); 1-way ANOVA with Tukey’s multiple-comparison test (**D**–**F**, **J**, **L**–**N**, **Q**–**T**, and **Y**); Spearman’s correlation test (**G**); 2-way ANOVA test with repeated measures (**H**); paired Student’s *t* test (**I**); or 2-tailed unpaired Student’s *t* test (**U** and **X**).

To assess the contribution of commensal fungi to colitis, intestinal fungi were depleted before DSS treatment. Fluconazole-treated WT mice had reduced body weight loss, disease severity, colon shortening, epithelial damage, and inflammatory infiltration compared with untreated WT mice (Supplemental Figure 4, D–G, and Figure 4, B and C). Populations of neutrophils and IL-17^+^CD4^+^ T cells were reduced, whereas Tregs increased to levels comparable to those in *Clec4n^–/–^* mice (Supplemental Figure 4, H and I). Correspondingly, *Il6*, *Tnf*, *S100a8*, and *S100a9* expression was markedly suppressed in WT mice (Supplemental Figure 4J and Figure 4D). In contrast, *Clec4n^–/–^* and *Card9^–/–^* mice showed no additional benefit from fungal depletion (Supplemental Figure 4, D–L, and Figure 4, E–H), indicating fungal exacerbation of colitis requires Dectin-2/CARD9 signaling.

*Engyodontium* colonization robustly induced colonic calprotectin (S100A8/S100A9) expression (Figure 1L and Supplemental Figure 1I), an effect impaired in *Clec4n*^–/–^ and *Card9*^–/–^ mice at steady state and after colonization (Figure 2F, Figure 3G, and Supplemental Figure 3, B and F). During DSS-induced colitis progression, neutrophils expanded by day 7 and remained elevated during recovery (day 35). *Clec4n* expression was enriched in activated neutrophils (*Cd177*^+^, *Csf3r*^+^) (Supplemental Figure 5I), and *S100a8*^+^*Clec4n*^+^ neutrophils peaked on day 7, correlating with *S100a8*/*S100a9* expression (Supplemental Figure 4M, and Figure 4, J and K).

Direct stimulation with the Dectin-2 agonist α-mannan (15) induced *S100a8*/*S100a9* in BM-derived neutrophils in a CARD9-dependent manner (Supplemental Figure 4, N and O). Similarly, cLP neutrophils stimulated with *Engyodontium* upregulated calprotectin (Supplemental Figure 4P). These results indicate that intestinal fungi, particularly *Engyodontium*, persistently activate neutrophils through the Dectin-2/CARD9 pathway to induce calprotectin production.

### Intestinal fungus–induced colitis exacerbation is mediated by commensal bacteria.

**Figure 5:**
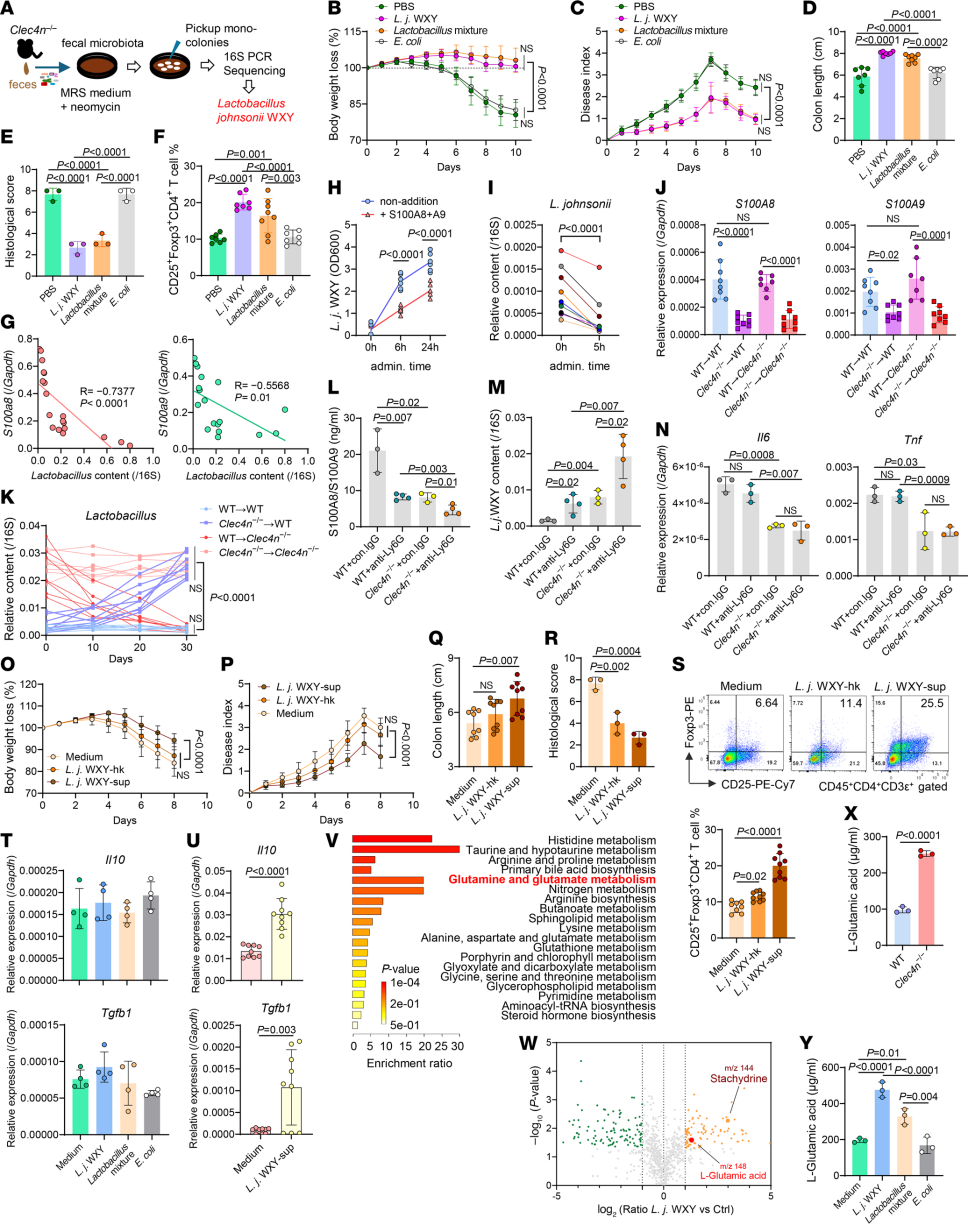
Attenuation of fungus-promoted colitis in Dectin-2–deficient mice is dependent on commensal microbiota. (**A**–**F**) WT and *Clec4n*^–/–^ mice were separated or co-housed for 4 weeks and subsequently treated with DSS while maintained in housing conditions. (**A**) Body weight loss and (**B**) DAI. (**C**) Gross colon morphology and length measurements at sacrifice on day 9. (**D**) Representative H&E-stained distal colon sections. (**E**) Frequencies of cLP neutrophils and Treg cells measured by flow cytometry. (**F**) Heatmap of immune-related gene expression in colon tissues assessed by qPCR (**A**–**C**, *n* = 9/co-housed group, WT separated *n* = 14, *Clec4n*^–/–^ separated *n* = 10; **D**, *n* = 6/group; **E**, *n* = 6/co-housed group, WT separated *n* = 10, *Clec4n*^–/–^ separated *n* = 7; **F**, *n* = 4/group). (**G**–**I**) WT and *Card9*^–/–^ mice were co-housed for 4 weeks and treated with DSS. DAI (**G**), histological analysis (**H**), and colon gene expression profiles (**I**) (**G**, *n* = 7/group; **I**, *n* = 4/group; **H**, *n* = 5/group). (**J**–**N**) Antibiotic-pretreated WT mice received fecal microbiota transplants from WT (WT f), *Clec4n*^–/–^ (*Clec4n*^–/–^ f), or *Card9*^–/–^ (*Card9*^–/–^ f) donor mice followed by DSS treatment. Body weight loss (**J**), DAI (**K**), gross colon morphology and length (**L**), distal colon histology (**M**), and cLP neutrophil and Treg frequencies (**N**) (**J**–**L**, and **N**, WT f, *n* = 9; *Clec4n*^–/–^ f, *n* = 10; *Card9*^–/–^ f, *n* = 10; **M**, *n* = 5/group). (**O**–**S**) 16S rDNA-Seq analysis of fecal microbiota from WT and *Clec4n*^–/–^ mice under physiological conditions, showing phylum-level composition (**O**), β diversity by PCoA (**P**), family-level abundance (**Q**), abundance of selected taxa (**R**), and detectable lactic acid bacteria species (**S**) (*n* = 4/group). (**T**) DAI in WT and *Clec4n*^–/–^ mice treated with DSS with or without vancomycin (Vanco) (WT, *n* = 6; *Clec4n*^–/–^, *n* = 8; *Clec4n*^–/–^ Vanco, *n* = 8). Data in **A**–**C**, and **G** are pooled from 3 independent experiments, and data in **D**, **E**, **J**–**L**, **N**, and **T** are pooled from 2 independent experiments. Data in **F** and **I** are from 1 of 2 independent experiments. Data in **A**–**E**, **G**, **H**, **J**–**L**, **R**, and **T** are presented as mean ± SD. Statistical analysis: 1-way ANOVA with Bonferroni’s multiple-comparison test (**A**, **B**, **J**, **K**, **T**), 1-way ANOVA with Tukey’s multiple-comparison test (**C**–**E** and **L**–**N**), 2-tailed unpaired Student’s *t* test (**F**, **H**, and **Q**), and 2-way ANOVA test with repeated measures (**G**).

Because intestinal inflammation is shaped by host genetics and microbiota (26), WT and *Clec4n*^–/–^ or *Card9*^–/–^ mice were co-housed for 4 weeks (Supplemental Figure 6A). Co-housed *Clec4n*^–/–^ or *Card9*^–/–^ mice developed DSS colitis comparable to WT mice, including body weight loss, colon shortening, epithelial damage, and immune infiltration (Figure 5, A–D, G, and H, and Supplemental Figure 6, B, C, and E–G). Treg frequencies also normalized across groups (Figure 5E and Supplemental Figure 6, D and H), suggesting microbiota-driven regulation. In contrast, mRNA expression of innate inflammatory genes (*Tnf*, *Il6*, *Cxcl2*, *S100a8*, *S100a9*) remained reduced in *Clec4n*^–/–^ or *Card9*^–/–^ mice (Figure 5, F and I), indicating their direct regulation by Dectin-2/CARD9 signaling.

To directly test microbiota dependence, antibiotic-treated WT mice received fecal microbiota from WT, *Clec4n*^–/–^, or *Card9*^–/–^ donors before DSS (Supplemental Figure 6I). Mice receiving *Clec4n*^–/–^ or *Card9*^–/–^ microbiota had milder disease severity, colon shortening, and histopathology (Figure 5, J–M, and Supplemental Figure 6J), along with increased numbers of colonic Tregs (Figure 5N and Supplemental Figure 6K).

16S rDNA-Seq revealed distinct bacterial communities in *Clec4n*^–/–^ mice despite similar species-level microbiome structure (Supplemental Figure 6L), including a reduced Verrucomicrobiota population (Figure 5, O and P). Populations of members of Akkermansiaceae decreased, whereas those of *Clostridia* UCG-014 and Lactobacillaceae increased markedly (>28-fold and >21-fold, respectively) (Figure 5, Q and R). A dominant *Lactobacillus* strain, *L*. *johnsonii* WXY (*L.j*. WXY), was abundant in *Clec4n*^–/–^ mice but absent in WT mice (Figure 5S).

Vancomycin-mediated depletion of Gram-positive bacteria abolished DSS resistance in *Clec4n*^–/–^ mice, restoring colitis severity and reducing Tregs to WT levels (Figure 5T and Supplemental Figure 6, N–Q). Neutrophil-associated genes remained suppressed (Supplemental Figure 6R), indicating their independence from bacterial changes. Collectively, Dectin-2/CARD9–dependent fungal sensing exacerbates colitis indirectly by reshaping Gram-positive bacteria that may support Treg-mediated protection.

### Calprotectin inhibits colonization of L.j. WXY, which ameliorates colitis via secreted metabolite(s).

We isolated the single strain *L.j*. WXY and a *Lactobacillus* strain mixture from *Clec4n*^–/–^ feces (Figure 4A). Colonization of antibiotic-treated WT mice with these strains, followed by WT fecal microbiota transfer and DSS challenge, significantly reduced weight loss, disease severity, colon shortening, epithelial damage, and inflammation compared with PBS or *E*. *coli* controls (Figure 4, B–E, Supplemental Figure 7, A–C). *L.j*. WXY increased cLP Foxp3^+^ Tregs (Figure 4F and Supplemental Figure 7D).

Calprotectin expression inversely correlated with *Lactobacillus* abundance (Figure 4G). Recombinant S100A8/S100A9 inhibited *L.j*. WXY growth in vitro (Figure 4H) and reduced fecal *L*. *johnsonii* after intrarectal administration, without affecting other bacteria (Figure 4I and Supplemental Figure 7E), indicating selective suppression.

BM chimera experiments showed that *Clec4n*^–/–^ BM in WT hosts reduced calprotectin and increased *Lactobacillus*, whereas WT BM in *Clec4n*^–/–^ hosts restored high levels of calprotectin and suppressed *Lactobacillus* (Figure 4, J and K). Neutrophil depletion similarly reduced calprotectin and increased *L.j*. WXY in both genotypes (Figure 4, L and M), without altering *Il6* or *Tnf* (Figure 4N), identifying neutrophils as key regulators of calprotectin-mediated *Lactobacillus* control.

Functionally, *L.j*. WXY culture supernatant, but not heat-killed bacteria, protected against DSS colitis, reduced tissue pathology, and increased the population of cLP CD25^+^Foxp3^+^ Tregs (Figure 4, O–S, and Supplemental Figure 7, F and G), indicating metabolite-mediated protection. Consistently, the supernatant induced *Il10* and *Tgfb1* in Tregs but not CD11b^+^ or CD11c^+^ cells (Figure 4, T and U).

Metabolomic profiling revealed enrichment of amino acid–related pathways in *L.j*. WXY–colonized mice (Figure 4V). Filtering identified 22 candidate metabolites (Supplemental Table 1); based on reported inflammatory effects (27–29) and availability, l-glutamic acid and stachydrine were selected (Figure 4W). *Clec4n*^–/–^ mice had elevated levels of l-glutamic acid (Figure 4X and Supplemental Figure 7H), and *L.j*. WXY produced substantial l-glutamic acid but not stachydrine (Figure 4Y and Supplemental Figure 7, H and I), identifying l-glutamic acid as a candidate metabolite for colitis protection.

### L.j. WXY inhibits colitis by producing l-glutamic acid to promote the Treg population.

**Figure 6:**
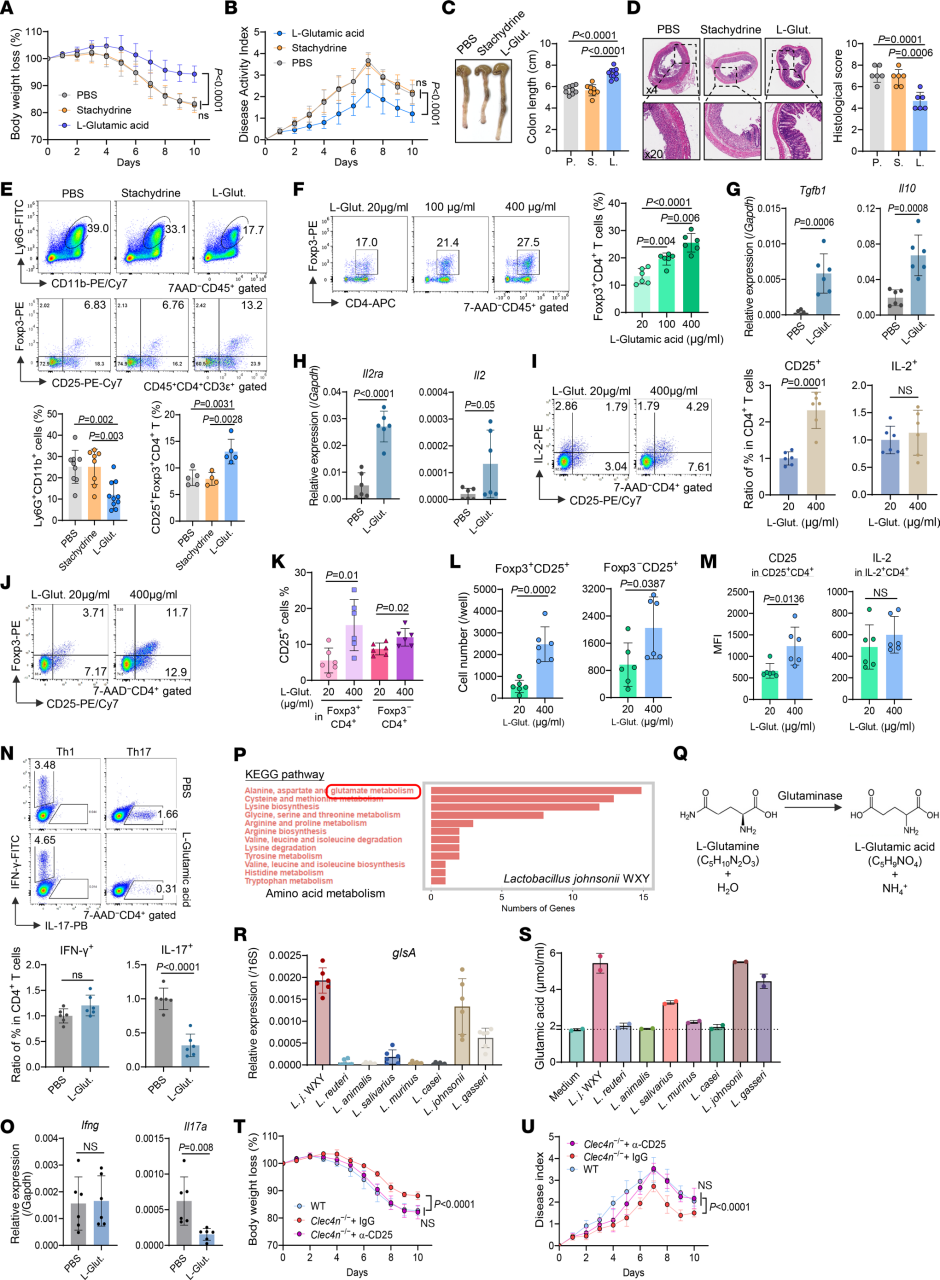
Glutamic acid secreted by *L.j.* WXY attenuates colitis by promoting Treg expansion through enhanced IL-2R signaling. (**A**–**E**) WT mice were orally administered l-glutamic acid (L-Glut.) or stachydrine daily for 3 days, followed by DSS treatment with continued daily metabolite administration sacrifice on day 10. Body weight loss (**A**), DAI (**B**), gross colon morphology and length (**C**), distal colon histology (**D**), and cLP neutrophil and Treg frequencies (**E**) (**A**–**C**, and **E**, PBS *n* = 9, stachydrine *n* = 8, l-glutamic acid *n* = 10; **D**, *n* = 6/group). P, PBS; S, Stachydrine; L, L-glutamic acid. (**F**–**H**) CD62L^+^ naive CD4^+^ T cells isolated from WT spleen and lymph nodes were polarized toward Treg differentiation and simultaneously stimulated with l-glutamic acid. After 6 days, Treg frequencies were assessed by flow cytometry (**F**), and mRNA expression of *Tgfb1* and *Il10* (**G**), as well as *Il2ra* and *Il2* (**H**), was quantified by qPCR after stimulation with 400 μg/mL l-glutamic acid (*n* = 6 biological replicates/group). (**I**) CD62L^+^ naive CD4^+^ T cells were activated with anti-CD3 and anti-CD28 Abs and treated with 400 μg/mL l-glutamic acid. After 4 days, proportions of IL-2^+^ and CD25^+^ cells among CD4^+^ T cells were analyzed by flow cytometry (*n* = 6 biological replicates/group). (**J**–**M**) CD62L^+^ naive CD4^+^ T cells were polarized toward Treg differentiation in the presence of 400 μg/mL l-glutamic acid for 6 days. The proportions (**J** and **K**) and absolute numbers (**L**) of CD25^+^Foxp3^+^ and CD25^+^Foxp3^–^ cells, as well as the MFI of CD25 in CD25^+^CD4^+^ and IL-2 in IL-2^+^CD4^+^ cells (**M**) were determined by flow cytometry (*n* = 6 biological replicates/group). (**N** and **O**) CD62L^+^ naive CD4^+^ T cells were polarized toward Th1 or Th17 differentiation in the presence of l-glutamic acid. After 5 days, the frequencies of IFN-γ^+^ and IL-17^+^ CD4^+^ T cells were measured by flow cytometry (**N**), and *Ifng* and *Il17a* mRNA expression was quantified by qPCR (**O**) (*n* = 6 biological replicates/group). (**P**) KEGG pathway annotation highlighting amino acid metabolism in *L.j*. WXY based on whole-genome sequencing. (**Q**) Chemical structural formula illustrating the metabolic conversion of l-glutamine to l-glutamic acid involving glutaminase. (**R**) Relative expression of the bacterial glutaminase-encoding gene *glsA* in the indicated *Lactobacillus* strains, normalized to 16S rDNA by qPCR (*n* = 6 technical replicates/group). (**S**) Indicated *Lactobacillus* strains (1 × 10^7^ CFU) were cultured in MRS medium for 24 hours, and glutamic acid concentrations in culture supernatants were measured using a glutamic acid assay kit (*n* = 2 replicates/group). (**T** and **U**) *Clec4n*^–/–^ mice were administered anti-CD25 neutralizing Ab or isotype control IgG intraperitoneally every other day for 3 doses, followed by 7 days of DSS treatment with continued Ab administration for an additional 5 doses. Body weight loss (**T**) and DAI (**U**) were monitored daily (control IgG, *n* = 6; anti-CD25 Ab, *n* = 8; WT, *n* = 7). Data in **A**–**O**, **R**, **T**, and **U** are pooled from 2 independent experiments. Data in **A**–**I**, **K**–**O**, and **R**–**U** are presented as mean ± SD. Statistical analysis: 1-way ANOVA with Bonferroni’s multiple-comparison test (**A**, **B**, **T**, and **U**); 1-way ANOVA with Tukey’s multiple-comparison test (**C**–**F**), or 2-tailed unpaired Student’s *t* test (**G**–**I** and **K**–**O**).

Intrarectal administration of l-glutamic acid markedly alleviated DSS-induced colitis, reducing body weight loss, colon shortening, epithelial damage, and inflammatory infiltration compared with stachydrine or PBS (Figure 6, A–D, and Supplemental Figure 8A). l-Glutamic acid treatment decreased neutrophil infiltration and increased cLP Foxp3^+^ Treg cells (Figure 6E).

In vitro, l-glutamic acid dose dependently enhanced Foxp3^+^CD4^+^ Treg differentiation and enhanced their *Il10* and *Tgfb1* expression (Figure 6, F and G). Mechanistically, l-glutamic acid upregulated *Il2ra* (CD25), but not *Il2*, increasing the proportion of CD25^+^ in CD4^+^ T cells without affecting IL-2 production (Figure 6, H and I). CD25 induction occurred in both Foxp3^+^ Treg and Foxp3^–^ subsets (Figure 6, J–L), indicating global enhancement of IL-2 receptor (IL-2R) availability. l-Glutamic acid also intensified CD25 expression per CD25^+^ T cell without altering IL-2 intensity (Figure 6M). Consistent with enhanced IL-2R signaling, l-glutamic acid suppressed IL-17 but not IFN-γ production by CD4^+^ T cells (Figure 6, N and O), in line with competitive STAT5-STAT3 regulation (30).

Genome sequencing and Kyoto Encyclopedia of Genes and Genomes (KEGG) analysis of *L.j*. WXY revealed enrichment of the glutamate metabolism pathway (Figure 6P and Supplemental Figure 8B). Expression of glutaminase (*glsA*), which converts l-glutamine to l-glutamic acid, was high in *L.j*. WXY and *L.j*. NBRC13952, correlating with robust l-glutamic acid production, whereas *L*. *gasseri* LG21 had less expression and production (Figure 6, Q–S).

To assess the functional importance of IL-2 signaling in vivo, *Clec4n*^–/–^ mice were treated with anti-CD25 Ab before DSS exposure (Supplemental Figure 8C). IL-2Rα blockade abolished the colitis resistance of *Clec4n*^–/–^ mice, restoring disease severity, colon shortening, epithelial damage, inflammatory infiltration, and reducing CD25^+^Foxp3^+^ Tregs to WT levels (Figure 6, T and U, and Supplemental Figure 8, D–G). These results indicate that l-glutamic acid–driven IL-2R signaling is essential for Treg-mediated protection under Dectin-2 deficiency.

### Engyodontium–DECTIN-2 axis in the human gut potentially contributes to IBD development.

**Figure 7:**
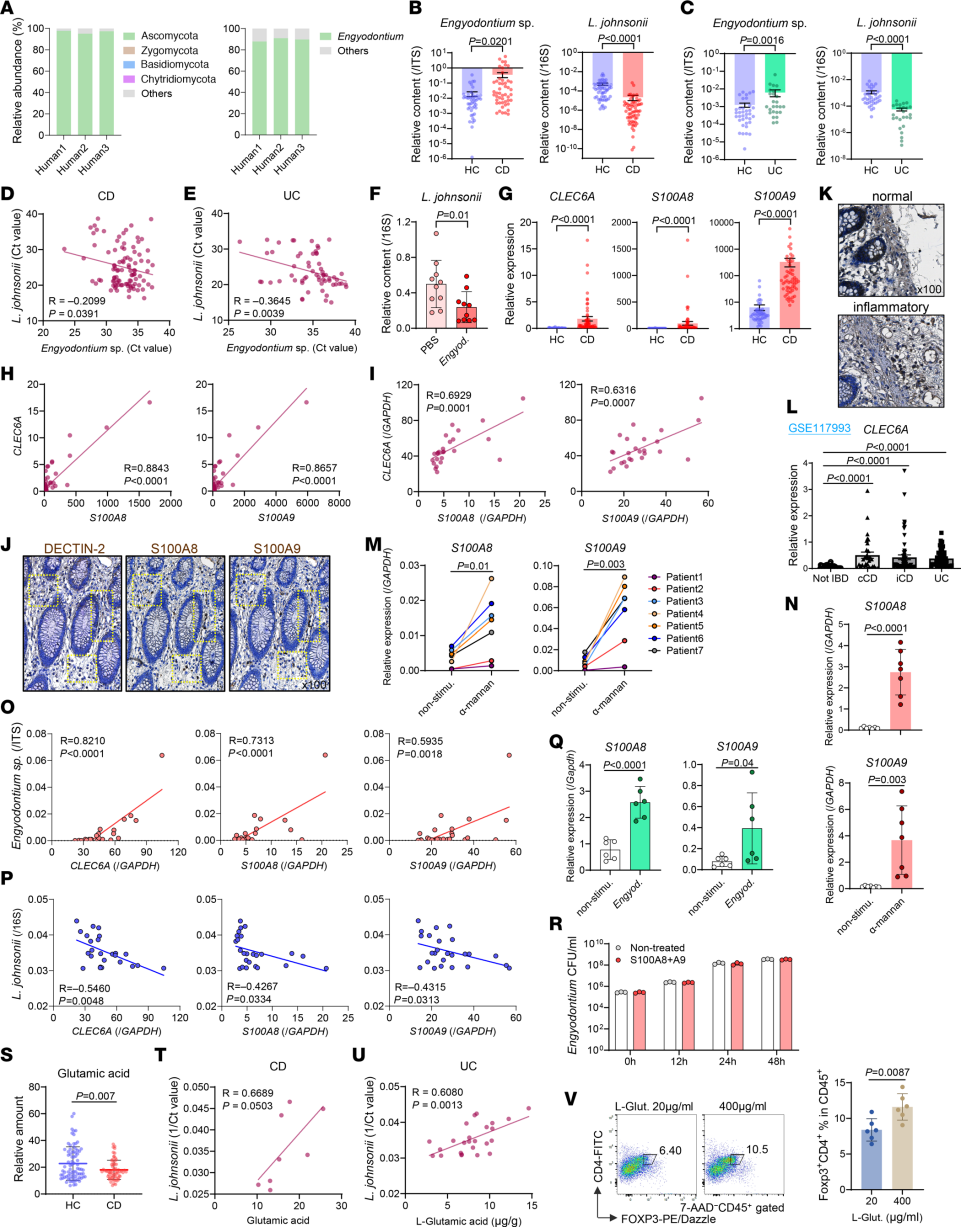
The *Engyodontium*–DECTIN-2 axis in the pathogenesis of human IBD. (**A**) Phylum- and genus-level composition of human fecal commensal fungi isolated using Dectin-2–Fc and identified by qPCR with species-specific primers. (**B**) Relative contents of *Engyodontium* sp. in fecal fungi and *L.j*. in fecal bacteria from patients with CD and healthy individuals (HC), determined by qPCR (*Engyodontium*: HC, *n* = 59; CD, *n* = 62. *Lacutobacillus*: HC, *n* = 62; CD, *n* = 63). (**C**) Relative contents of *Engyodontium* sp. in fecal fungi and *L.j*. in fecal bacteria from patients with UC and healthy individuals, determined by qPCR (HC, *n* = 36; UC, *n* = 25). (**D**) Correlation between Ct value of *L.j*. and *Engyodontium* sp. from human individuals as described in **B** (*n* = 97 [including HC, *n* = 48 and CD, *n* = 49]). (**E**) Correlation between Ct value of *L.j*. and *Engyodontium* sp. from human individuals as described in **C** (n=61 [including HC, n=36 and UC, n=25]). (**F**) C57BL/6J SPF mice were orally colonized with *Engyodontium* sp. (*Engyod.*) every other day for 3 administrations. Thirteen days later, fecal samples were collected, and the relative abundance of fecal *L.j*. was quantified by qPCR (*n* = 10 /group). (**G**) Relative expression of *CLEC6A*, *S100A8*, and *S100A9* in colon tissues from patients with CD and healthy control (HC) individuals, determined by bulk RNA-Seq analysis (HC, *n* = 47; CD, *n* = 57). (**H**) Correlation between *CLEC6A* and *S100A8* or *S100A9* expression based on bulk RNA-Seq data described in **G**. (**I**) Correlation between *CLEC6A* and *S100A8* or *S100A9* relative expression in colon tissues of patients with UC, determined by qPCR (*n* = 25). (**J**) IHC staining for DECTIN-2 and S100A8/S100A9 proteins in serial inflammatory colon sections obtained from a patient with CD after surgical resection. (**K**) IHC staining for human DECTIN-2 in paired normal and inflamed colon regions from the patient with CD shown in **J**. (**L**) Transcriptional levels of *CLEC6A* in colon tissues from non-IBD control individuals and patients with colonic CD (cCD), ileal CD (iCD), or UC, analyzed by reprocessing a public bulk RNA-Seq dataset (GSE117993) (not IBD, *n* = 55; cCD, *n* = 31; iCD, *n* = 60; UC, *n* = 43). (**M**) Colon tissues from 3 patients with IBD were obtained after resection. cLP CD11b^+^ cells were isolated and stimulated with α-mannan in vitro for 6 hours. *S100A8* and *S100A9* relative expression was quantified by qPCR (*n* = 7). (**N**) Neutrophils isolated from peripheral blood of 2 patients with IBD were stimulated with α-mannan in vitro for 6 hours, and *S100A8* and *S100A9* relative expression was measured by qPCR (*n* = 7 technical replicates from 2 biological replicates/group). (**O**) Correlation between fecal *Engyodontium* sp. abundance and colonic expression of *CLEC6A*, *S100A8*, or *S100A9* in patients with UC (*n* = 25). (**P**) Correlation between fecal *L.j*. abundance and colonic expression of *CLEC6A*, *S100A8*, or *S100A9* in patients with UC (*n* = 25). (**Q**) CD11b^+^ cells isolated from cLP of resected colon tissues from 2 patients with IBD were stimulated with *Engyodontium* sp. for 24 hours in vitro, followed by qPCR analysis of *S100A8* and *S100A9* expression (*n* = 6 technical replicates from 2 biological replicates/group). non-stimu., nonstimulated. (**R**) *Engyodontium* sp. was cultured at room temperature with recombinant S100A8 + S100A9 (1:1 mixture, 5 μg/mL each) for 12, 24, and 48 hours. Fungus growth was quantified by CFU enumeration on PDA plates (*n* = 3 replicates/group). (**S**) Relative amount of glutamic acid in fecal samples from patients with CD and healthy control individuals, assessed by liquid chromatography for untargeted metabolomics (HC, *n* = 80; CD, *n* = 65). (**T**) Correlation between 1/Ct value of *L.j*. and fecal glutamic acid levels in patients with CD (*n* = 8). (**U**) Correlation between 1/Ct value of *L.j*. and glutamic acid concentration in colon tissues from patients with UC (*n* = 25). (**V**) Peripheral blood leukocytes from 2 healthy donors were isolated and induced toward Treg differentiation in the presence of l-glutamic acid. After 5 days, the proportion of Foxp3^+^CD4^+^ among CD45^+^ leukocytes was analyzed by flow cytometry (*n* = 6 technical replicates from 2 biological replicates/group). Data in **F**, **M**, **N**, **Q**, and **V** are pooled from 2 independent experiments. Data in **B**, **C**, **G**, and **L** are presented as mean ± SEM, and in **F**, **N**, **Q**, **R**, **S**, and **V** as mean ± SD. Statistical analysis: 2-tailed Mann-Whitney test (**B**, **C**, and **G**), 2-tailed unpaired Student’s *t* test (**F**, **N**, **Q**–**S**, and **V**), 1-way ANOVA with Kruskal-Wallis and Dunn’s test (**L**), Spearman’s correlation test (**D**, **E**, **H**, **I**, **O**, **P**, **T**, and **U**), or paired Student’s *t* test (**M**).

Using a Dectin-2-Fc fusion protein, Dectin-2–binding fungi were isolated from feces of healthy individuals and were dominated by Ascomycota members, with *Engyodontium* as the major genus (Figure 7A). In fecal samples from patients with CD, *Engyodontium* abundance was significantly increased (18-fold), whereas that of *L*. *johnsonii* was markedly reduced (23-fold) compared with healthy controls (Figure 7B). Similar microbial changes were observed in patients with UC (Figure 7C). *Engyodontium* and *L*. *johnsonii* abundances were inversely correlated, suggesting competitive exclusion (Figure 7, D and E), consistent with reduced *L*. *johnsonii* in *Engyodontium*-colonized mice (Figure 7F). Total *Lactobacillus* abundance did not differ significantly between groups (Supplemental Figure 9A).

Bulk RNA-Seq of colonic tissues revealed increased *CLEC6A* (DECTIN-2), *S100A8*, and *S100A9* expression in inflamed CD tissues (Figure 7G), with significant correlations between *CLEC6A* and calprotectin in CD and UC cohorts (Figure 7, H and I). IHC confirmed colocalization of DECTIN-2 with S100A8/S100A9-positive cells and extensive infiltration of DECTIN-2–positive inflammatory cells in inflamed regions (Figure 7, J and K). Public RNA-Seq data (GSE117993) showed elevated *CLEC6A* in inflamed UC and CD tissues, correlating with neutrophil markers (Figure 7L, Supplemental Figure 9B). scRNA-Seq (GSE202052) localized *CLEC6A* expression predominantly to neutrophils expressing *CSF3R*, *G0S2*, and *S100A8*/*S100A9* (Supplemental Figure 9, C–E). Consistently, α-mannan stimulation induced calprotectin in human CD11b^+^ cLP cells and peripheral neutrophils (Figure 7, M and N).

*Engyodontium* abundance positively correlated with *CLEC6A* and calprotectin levels, whereas there was inverse correlation of *L*. *johnsonii* populations in patients with UC (Figure 7, O and P), recapitulating the mechanism observed in murine models. *Engyodontium* directly induced calprotectin in human CD11b^+^ cells (Figure 7Q); calprotectin did not inhibit *Engyodontium* growth (Figure 7R).

Metabolomic analysis revealed reduced fecal glutamic acid in patients with CD, with no change in glutamine (Figure 7S and Supplemental Figure 9F). Glutamic acid levels positively correlated with *L*. *johnsonii* abundance in patients with CD and those with UC (Figure 7, T and U). Moreover, l-glutamic acid promoted human FOXP3^+^ Treg differentiation in vitro (Figure 7V). Public scRNA-Seq data showed broad expression of glutamate transporters (*SLC3A2*, *SLC7A5*) across T cell subsets (Supplemental Figure 9, G–I). Lastly, neither *Engyodontium* nor *L*. *johnsonii* abundance correlated with clinical parameters in patients with CD (Supplemental Figure 10, A and B).

Collectively, these data mirror murine findings and support a conserved *Engyodontium*–DECTIN-2–calprotectin axis in humans, providing a mechanistic framework for how commensal fungi shape intestinal microbiota and the immune system that influence IBD consequently.

## Discussion

Most intestinal fungi detected by ITS rDNA-Seq are difficult to isolate because many originate from diet or water, lack thermotolerance, or require strict anaerobic conditions incompatible with standard cultivation. Consequently, sequencing signals may reflect transient fungi or DNA from dead organisms. Nevertheless, even nonviable or transient fungi may exert sustained immunological effects through conserved cell-wall polysaccharides that stimulate host immune receptors.

Using a CLR-Fc–based purification strategy, we isolated a colonic commensal *Engyodontium* sp. from SPF mouse feces. This fungus exacerbated colitis by activating the Dectin-2/CARD9 pathway in neutrophils, promoting mucosal inflammation and suppressing *Lactobacillus*-mediated Treg support. We identified S100A8/S100A9 calprotectin as a downstream effector of *Engyodontium*–Dectin-2 signaling. Consistent with its antimicrobial activity against Gram-positive bacteria (31, 32), calprotectin-sensitive taxa, including *Lactobacillus*, *Clostridia*, and members of Erysipelotrichaceae, were increased in *Clec4n*^–/–^ mice (Supplemental Figure 6M). The isolated *L.j*. WXY strain expressed *glsA* and produced l-glutamic acid, which enhanced Treg differentiation via IL-2R signaling.

Recent work identified *Kazachstania pintolopesii* as a commensal fungus promoting Th2 responses and allergic inflammation in mice (33). Notably, this species was rarely detected in our facility and was absent in animals housed at The Jackson Laboratory (33), highlighting substantial facility-dependent heterogeneity in mycobiome composition. Such contextual differences likely shape divergent immune outcomes, emphasizing the importance of isolating and functionally characterizing locally relevant fungal species.

Calprotectin (S100A8/S100A9), abundantly expressed by neutrophils and macrophages, mediates antimicrobial defense and serves as a clinical biomarker for IBD and arthritis (31, 34, 35). Our data indicate that *Engyodontium*-induced calprotectin suppresses probiotic *Lactobacillus* colonization, thereby disrupting immune homeostasis.

At steady state, *Engyodontium* markedly increased colonic neutrophils, whereas *Clec4n*^–/–^ mice had reduced numbers of neutrophils and expanded Treg populations. Correspondingly, populations of Firmicutes and *L*. *johnsonii* declined following *Engyodontium* colonization, supporting a model in which neutrophil-derived antimicrobials suppress Treg-supportive bacteria. Although bacteria frequently antagonize fungi (e.g., antimicrobial peptide–producing commensal bacteria inhibit *Candida* *albicans*, ref. 36; and *Lactobacillus* species restrict fungal growth and filamentation, ref. 37), examples of fungi suppressing bacteria are limited. Notable exceptions include *Candida*-mediated suppression of bacterial species via candidalysin (38). Our findings demonstrate that *Engyodontium* reduces *L*. *johnsonii* colonization, reshaping host immunity and exacerbating colitis.

Neutrophil depletion reduced calprotectin levels and restored *L*. *johnsonii* colonization, confirming neutrophils as key mediators of fungus-driven microbiota disruption. Despite limited proliferation, *Engyodontium* persisted at high levels, consistent with other Ascomycota species. Although optimal growth occurred at room temperature, *Engyodontium* evaded immune clearance in CLR- and TLR-sufficient hosts. Its epithelial proximity induced CXCL1/2 via TLR/TRAF6 signaling, promoting neutrophil recruitment independently of CLR/SYK pathways.

Heat-killed *L.j*. WXY modestly reduced histological inflammation without affecting body weight, colon length, or Treg frequency, indicating that bacterial structural components may confer Treg-independent benefits. Previous studies showed *Lactobacillus* *reuteri* ameliorates colitis by inducing IL-10–producing dendritic cells (39). *Lactobacillus* cell wall components, including peptidoglycan and teichoic acids, modulate immune signaling (40), although additional bioactive molecules likely contribute.

Microbial metabolites are key regulators of mucosal immunity (41). *Clostridia* spp*.–*derived butyrate promotes Foxp3 expression, whereas *L*. *reuteri* and *L*. *NK2* metabolize arginine into l-ornithine to induce IL-22 from type 3 innate lymphoid cells (42). Although *Lactobacillus* species commonly produce lactic acid and bacteriocins (43), glutamic acid production is less well characterized (44). We found that *L*. *johnsonii* strains produce substantial l-glutamic acid via *glsA*, promoting Treg expansion. Other glutamate-producing microbes, including *Corynebacterium* *glutamicum* and *Lactococcus* *lactis*, may similarly influence immune regulation (45).

Glutamic acid regulates T cell fate through multiple metabolic pathways, including glutamate/glutathione and glutamate/α-KG axes (46). GOT1-mediated conversion of α-KG to glutamate influences Th17–Treg balance: genetic deletion of GOT1 inhibits Treg and enhances Th17 differentiation (47). Consistent with this finding, glutamic acid increased Foxp3 and suppressed *Il17a* expression while upregulating CD25, suggesting reinforcement of IL-2R signaling to support Treg expansion.

Collectively, our data identify Dectin-2 as a sensor of commensal *Engyodontium* that drives neutrophil-mediated suppression of *Lactobacillus* colonization and Treg development. Although targeting this axis may alleviate mucosal inflammation, complete inhibition could compromise antifungal immunity, because Dectin-2 and CARD9 are essential for host defense. In patients with IBD, elevated *Engyodontium* abundance, increased DECTIN-2 and calprotectin expression, reduced numbers of *L*. *johnsonii*, and lower glutamic acid levels mirrored our experimental findings. Correlations observed in UC cohorts further support an antagonistic fungal–bacterial axis in human disease. These observations may mirror the phenomenon seen in mice, with the inflammatory axis dominating in disease. Given that *Engyodontium* induces calprotectin in human neutrophils and glutamic acid promotes human Treg differentiation, targeting the *Engyodontium*–Dectin-2 pathway may represent a promising therapeutic strategy for IBD.

## Methods

### Sex as a biological variable.

Sex was not considered a biological variable for patient samples. Both male and female mice were used in the study except for the in vivo DSS-induced colitis experiments, in which male mice were mainly used because some female mice could not develop typical colitis symptoms, whereas these were stably developed in most male mice.

### Human samples.

Colon tissues from patients with CD were collected after resection for IHC analysis of DECTIN-2 and calprotectin. In selected experiments, CD11b^+^ cells were isolated from cLP tissues, stimulated with α-mannan for 6 hours, and calprotectin expression was analyzed by qPCR. Peripheral blood from 2 patients with CD was collected, and neutrophils were isolated by Ficoll gradient centrifugation. Residual erythrocytes were removed by brief hypotonic lysis (600 μL of distilled water for 5–10 seconds followed by 200 μL of 4× PBS). Purified neutrophils were stimulated with α-mannan for 6 hours and analyzed by qPCR.

For transcriptomic correlation analyses, inflammatory colon tissues from 57 patients with CD and normal colon tissues from 47 healthy individuals were analyzed by bulk RNA-Seq. Fecal samples from 65 patients with CD and 95 healthy individuals were collected for qPCR-based quantification of *Lactobacillus* and *Engyodontium*. Additionally, fecal samples from 65 patients with CD and 80 healthy control individuals were analyzed for glutamine and glutamic acid content using untargeted liquid chromatography–based (LC-based) metabolomics. Fecal and colonic samples from 25 patients with UC and 36 healthy individuals were collected for qPCR-based quantification of *Lactobacillus* and *Engyodontium* abundance, and *CLEC6A*, *S100A8*, and *S100A9* relative expression, respectively.

### Mice.

*Clec4n*^–/–^ and *Card9*^–/–^ mice on a C57BL/6J background were generated using CRISPR-Cpf1 technology at the Center for Animal Disease Models, Tokyo University of Science. Heterozygous founders were backcrossed to C57BL/6J mice to obtain homozygous mutant lines. WT C57BL/6J mice (Sankyo Lab Service) were used as controls. All mice were maintained under SPF conditions with a γ-irradiated diet, acidified water (0.002N HCl, pH 2.5), and autoclaved bedding at Sun Yat-sen University animal facilities. Mice aged 8–10 weeks were used.

### Generation of Clec4n^–/–^ and Card9^–/–^ mice.

Guide RNAs targeting exon 2 of *Card9* and exons 1 and 4 of *Clec4n* were designed to include protospacer-adjacent motif sequences (24 bp) (Supplemental Table 3) and synthesized by Integrated DNA Technologies (IDT). A mixture of 1.2 mM CRISPR RNA and 0.6 mM CRISPR-Cpf1 nuclease (IDT, 1081068) was microinjected into C57BL/6J zygotes. Two-cell embryos were transferred into pseudopregnant females. Genotypes were confirmed by RT-PCR and sequencing (primers are listed in Supplemental Table 1). A *Clec4n* mutant carrying a >5.7 kb deletion spanning exon 1 to exon 4 (including ATG) was used to establish the *Clec4n*^–/–^ line. For *Card9*^–/–^, a 17 bp deletion in exon 2 caused a frameshift producing a truncated 101-aa protein, and this line was expanded to homozygosity.

### DSS-induced colitis.

Acute colitis was induced by administering 1.5% DSS (9011-18-1, MP Biomedicals) in drinking water for 7 days, followed by normal water for 2–3 days. Disease activity was scored daily based on stool consistency, bleeding, and body weight loss. The disease activity index (DAI) was calculated as the mean of these 3 parameters. Mice were sacrificed on days 9 or 10 for analysis.

### Isolation of cLP cells.

Colons were excised, washed with PBS, and cut into approximately 2 mm fragments. Tissues were incubated in HBSS containing 3 mM EDTA at 37°C for 30 minutes with agitation to remove epithelial cells. After washing, tissues were digested in RPMI-1640 medium supplemented with 10% FCS, collagenase (400 U/mL; C5138-1, Sigma-Aldrich), and DNase (5 U/mL; D8070, Solarbio) at 37°C for 120 minutes. Single-cell suspensions were obtained by vortexing and filtration. In some experiments, lymphocytes were enriched using a 45%/66.6% Percoll (17-0891-01, Cytiva Life Sciences) gradient (400*g*, 20 minutes, 22°C).

### Flow cytometry.

Cells were washed in FACS buffer (HBSS with 2% FCS) and Fc receptors were blocked with 2.4G2 (Tonbo Biosciences, catalog 70-0161-U500). Surface staining was performed using fluorophore-conjugated Abs against mouse CD45 (catalog 103116), CD11b (catalog 101216), Ly6G (catalog 127606), Ly6C (catalog 128007), CD11c (catalog 117322), F4/80 (catalog 123115), CD3ε (catalog 100305), CD4 (catalog 100411), CD8α (catalog 100707), CD25 (catalog 102015), and I-A/I-E (catalog 107614) (all BioLegend). For intracellular cytokine staining, cells were stimulated with PMA (50 ng/mL; P1585, Merck Millipore) and ionomycin (500 ng/mL; 56092-82-1, Merck Millipore) in the presence of 1 mM monensin (475895, Merck Millipore) for 5 hours, fixed, permeabilized (BD Cytofix/Cytoperm; 554722, BD Biosciences), and stained for IL-17A (catalog 506918), IFN-γ (catalog 505806), or IL-2 (catalog 503805). Foxp3 (126403, BioLegend) staining was performed using the Foxp3/Transcription Factor Fixation/Permeabilization kit (00-5123-43, Thermo Fisher Scientific). Dead cells were excluded using 7-aminoactinomycin D. Human Tregs were identified by staining for CD45 (catalog 368526), CD4 (catalog 300506), CD25 (catalog 302606), and FOXP3 (catalog 320126) (all BioLegend). Data were acquired on Attune NxT or CytoFLEX cytometers and analyzed using FlowJo, version 10.1 (BD Biosciences). Gating strategy for cLP neutrophils and Tregs of mice is shown in Supplemental Figure 8H. Information of all antibodies used in the study is listed in Supplemental Table 4.

### RT-qPCR.

Total RNA was extracted using the Mammalian Total RNA Miniprep Kit (RTN350-1KT, Sigma-Aldrich) and reverse transcribed using Evo M-MLV RT Master Mix (AG11706, Agbio). qPCR was performed using SYBR Green Premix Pro Tag HS (Ag11701, Agbio) on a QuantStudio 5 system ((Thermo Fisher Scientific). Relative gene expression was calculated using the ΔCt method and normalized to *Gapdh*. Primer sequences are listed in Supplemental Table 3.

### IHC.

Colon tissues from patients with CD were fixed in 10% formalin, paraffin-embedded, and sectioned at 5 μm. Sections were deparaffinized, rehydrated, treated with H_2_O_2_, and subjected to heat-induced antigen retrieval at 60°C overnight. After blocking with 1% BSA, sections were incubated overnight at 4°C with rabbit Abs against DECTIN-2 (catalog DF10182), S100A8 (catalog DF6556), or S100A9 (catalog DF7596) (all 1 μg/mL and all from Affinity Biosciences). HRP-conjugated secondary Ab (GB23303, Servicebio) was applied for 15 minutes, followed by DAB development and hematoxylin counterstaining. Images were captured using a Leica DM6FS microscope (Leica Camera AG).

### Fecal microbiota DNA isolation and quantitation.

Mouse fecal samples were collected, and total microbial DNA was extracted using the QIAamp DNA Stool Mini Kit (51504, Quiagen). Bacterial genomic DNA was quantified by real-time RT-PCR on a QuanStudio5 system (Thermo Fisher Scientific), using 20 ng DNA and primers listed in Supplemental Table 2. Relative bacterial abundance was calculated by the ΔCt method and normalized to 16S rDNA expression.

### 16S rDNA-Seq analysis.

Bacterial genomic DNA (>500 ng) was subjected to 16S rDNA-Seq by Lian Chuan Biotechnology Co. The V3–V4 region was amplified using primers 338F (5′-ACTCCTACGGGAGGCAGCAG-3′) and 806R (5′-GGACTACHVGGGTWTCTAAT-3′). PCR products were verified on 2% agarose gels, purified with AMPure XT beads (Beckman Coulter Genomics), and quantified using Qubit (Invitrogen, Thermo Fisher Scientific). Libraries were assessed using a 2100 Bioanalyzer (Agilent) with the Library Quantification Kit for Illumina (Kapa Biosciences), spiked with 30% PhiX Control, version 3 (Illumina), and sequenced on an Illumina MiSeq platform (2 × 300 bp).

Paired-end reads were demultiplexed, trimmed, merged with FLASH (http://ccb.jhu.edu/software/FLASH/), filtered using Fqtrim version 0.94 (http://ccb.jhu.edu/software/fqtrim/), and checked for chimeras using Vsearch, version 2.3.4 (https://github.com/torognes/vsearch). Operational taxonomic units OTUs were clustered at 97% similarity with Vsearch and taxonomically annotated using the SILVA database (https://www.arb-silva.de/; accessed Feb. 1, 2022). Phylogenetic analysis was performed with Mafft, version 7.310 (https://mafft.cbrc.jp/alignment/server/index.html). OTU tables were normalized to the lowest sequencing depth. The α diversity (Chao1, Shannon) and β diversity (PCoA, clustering) analyses were conducted using QIIME 1.8.0 (https://qiime2.org/).

### Histological analysis.

Colon tissues were fixed in 10% neutral buffered formalin, paraffin-embedded, sectioned, and stained with H&E. Histological scores were calculated as the sum of epithelial damage and inflammatory infiltration, as follows: for epithelial damage: 0, normal; 1, goblet cell loss; 2, extensive goblet cell loss; 3, crypt loss; 4, extensive crypt loss. For inflammatory infiltration: 0, none; 1, pericryptal infiltration; 2, infiltration to muscularis mucosae; 3, extensive infiltration with edema; 4, submucosal infiltration.

### Antibiotic treatment and fecal microbiota transfer.

Mouse fecal pellets (*n* = 2–3) were homogenized in PBS, filtered through a 100 μm nylon mesh, centrifuged at 5,000*g* for 10 minutes at 4°C, and washed twice with PBS. The bacterial suspension was orally transferred into mice pretreated with an antibiotic cocktail containing kanamycin (400 mg; catalog 25389-94-0), gentamicin (35 mg; catalog 1405-41-0), colistin (57 mg; catalog 1264-72-8), metronidazole (215 mg; catalog 443-48-1), vancomycin (45 mg; catalog 1404-93-9), and erythromycin (10 mg; catalog 114-07-8; all from Macklin) in 1 L of drinking water (CEGKMV-ABX), followed by 1.5% DSS. In some experiments, *Clec4n*^–/–^ mice received vancomycin alone for 1 week, followed by DSS treatment.

### Culture and identification of Lactobacillus strains.

Mouse fecal samples were plated on De Man–Rogosa–Sharpe (MRS) agar (Solarbio) supplemented with neomycin (1405-10-3, Macklin) and incubated for 2 days. Single colonies were expanded, genomic DNA extracted, and 16S rDNA amplified using primers (forward: 5′-TGGAAACAGRTGCTAATACCG-3′; reverse: 5′-GTCCATTGTGGAAGATTCCC-3′) for sequencing-based identification. *Lactobacillus* strains were cultured in MRS broth at 37°C under anaerobic conditions.

### Antimicrobial peptide treatment.

*Lactobacillus* cultures were incubated in MRS medium containing a 1:1 mixture of recombinant S100A8 and S100A9 peptides (5 μg/mL each; HY-P71076, MCE) for 6 or 24 hours under anaerobic conditions. Bacterial growth was measured using a NanoPhotometer N60 (IMPLEM). For in vivo treatment, mice were intrarectally administered recombinant S100A8/S100A9 for 5 hours. Feces were collected before and after treatment, and *Lactobacillus* abundance was quantified by real-time RT-PCR normalized to 16S rDNA.

### BM transfer.

WT and *Clec4n*^–/–^ mice (6–8 weeks old) were irradiated with 6.5 Gy x-rays. BM cells (1 × 10^6^) from WT or *Clec4n*^–/–^ donors were intravenously transferred into recipients. Fecal samples were collected on days 0, 10, 20, and 30 after transfer to quantify *Lactobacillus* levels by real-time RT-PCR.

### BM-derived macrophages and neutrophils.

BM cells from femurs, tibias, and humeri were cultured in RPMI-1640 medium supplemented with 10% FBS and 1% penicillin–streptomycin after ACK-mediated erythrocyte lysis. For BM-derived macrophages, cells were differentiated with 30 ng/mL macrophage colony-stimulating factor (315-02, PeproTech), with medium replacement on day 4 and harvest on day 7. For BM-derived neutrophils, cells were cultured with 20 ng/mL granulocyte colony-stimulating factor (250-05, PeproTech) for 6 days. In some experiments, α-mannan (9036-88-8, Sigma-Aldrich) was added for 24 hours.

### Metabolomics analysis.

Untargeted metabolomics was performed using LC–tandem mass spectrometry (LC-MS/MS). Cecal supernatants (100 μL) were extracted with methanol/acetonitrile (1:1, 400 μL), sonicated for 20 minutes at 4°C, precipitated at –20°C for 30 minutes, centrifuged at 13,000*g* for 15 minutes, and dried under nitrogen. LC-MS/MS analysis was conducted on a UHPLC-Q Exactive HF-X system (Thermo Fisher Scientific). Data were processed using Progenesis QI (Waters) (https://www.nonlinear.com/) and annotated against Metlin (https://metlin.scripps.edu) and the Majorbio Databases (https://www.majorbio.com/) (All accessed June 7, 2023). Metabolites were identified by Student’s *t* test (*P* < 0.05) and fold-change analysis. Targeted l-glutamic acid analysis was conducted in the same way using a standard from Solarbio (catalog SG8540).

### Treg/Th1/Th17 cell in vitro differentiation.

CD4^+^ T cells were isolated from WT mouse spleens and lymph nodes by negative selection using biotin-conjugated Abs against CD11b (catalog 101203), CD11c (catalog 117303), I-A/I-E (catalog 107603), CD19 (catalog 115504), TCRγδ (catalog 118103), NK1.1 (catalog 108703), and CD8α (catalog 100704) (all from BioLegend), followed by anti-biotin microbeads (130-090-485, Miltenyi Biotec) and autoMACS separation. Naive CD4^+^ T cells were further purified using anti-CD62L microbeads (130-049-701, Miltenyi Biotec). Cells were stimulated with anti-CD3/CD28 Dynabeads (8 × 10^4^ beads/well; 11456D, Thermo Fisher Scientific). Treg differentiation was induced with TGF-β (5 ng/mL; 80116-RNAH, Sino Biological) and IL-2 (212-12, PeproTech) for 6 days. Th1 differentiation used IL-12 (5 ng/mL; 212-12, PeproTech) and anti–IL-4 Ab (5 μg/mL; 11B11, 504135, BioLegend). Th17 differentiation IL-6 (20 ng/mL; catalog 216-16, Gibco), TGF-β (5 ng/mL; 80116-RNAH, Sino Biological), IL-1β (10 ng/mL; catalog 211-11B, Gibco), IL-23 (10 ng/mL; catalog 200-23, Invitrogen), anti–IFN-γ Ab (catalog 505847, Biolgend), anti–IL-4 Ab (catalog 504135, BioLegend). l-Glutamic acid (100–400 μg/mL; IG0710, Solarbio) or *Lactobacillus* culture supernatant was added where indicated.

### Human Treg cell differentiation.

Human PBMCs were isolated from freshly collected peripheral blood using Lymphoprep (07851, STEMCELL Technologies). Cells were stimulated with Dynabeads Human T-Activator CD3/CD28 (8 × 10^4^ beads/well; 11161D, Thermo Fisher Scientific), recombinant TGF-β (5 ng/mL), and l-glutamic acid (400 μg/mL) for 5 days to induce Treg differentiation.

### Anti-CD25 Ab treatment.

*Clec4n*^–/–^ mice received intraperitoneal injections of anti-mouse CD25 Ab (100 μg; PC-61.5.3, BE0012, Bio X Cell) or control IgG (HRPN, BE0088, Bio X Cell) on days −5, −3, −1, 1, 3, 5, 7, and 9. Mice were administered 1.5% DSS on day 0 and euthanized on day 10.

### Preprocessing of scRNA-Seq data.

Preprocessed scRNA-Seq datasets from DSS-treated C57BL/6J mice (GSE168033) and patients with IBD (GSE202052) were obtained from the NCBI Gene Expression Omnibus (GEO) database and analyzed using Seurat (version 4.1.3) (https://github.com/satijalab/seurat/releases). Cells with fewer than 200 or more than 5,000 detected genes or greater than 5% mitochondrial reads were excluded. Data were normalized using the “LogNormalize” method (scale factor 10,000). After filtering, greater than 95% of high-quality cells expressing more than 20,000 protein-coding genes were retained for downstream analysis.

### Cell type identification and dimensional reduction for scRNA-Seq.

Highly variable genes were identified, and data were scaled and subjected to linear dimensional reduction. Cells were clustered based on feature gene expression and annotated using established marker genes: αβ T cells (*Icos*, *Trbc2*, *Il7r*), γδ T cells (*Trdc*, *Tcrg-C1*, *Trat1*), NK cells (*Nkg7*, *Ms4a4b*, *Gzmc*), macrophages (*Lyz2*, *Apoe*, *Ms4a6c*), cDCs (*Cst3*, *Cd74*, *Ccr7*), and neutrophils (*Ngp*, *Cd177*, *Mmp8*, *Csf3r*, *Il1rn*, *Cd300ld*).

### Generation of pFUSE-hIgG2-Dectin-1/2-Fc expression plasmids.

Dectin-1 and Dectin-2 coding sequences were amplified using the following primers: for Dectin-1, forward: 5′-CTTCACCTTGGAGGCCCATT-3′; reverse: 5′-TATTTCTGACTTGAAACGAGTTGGG-3′; for Dectin-2, forward: 5′-CACGAATTCATTTGGCTCCAGCTGCTACCTC-3′; reverse: 5′-GAACCCAATCTTCCAGAAAGATCTGTG-3′.

PCR was performed in a 30 μL reaction containing *Thermococcus kodakaraensis* (KOD) FX Neo (0.15 μL;), primers (1 μL each, 10 mM); deoxynucleotides (dNTPs) (6 μL); KOD buffer (15 μL); sterile water (4.85 μL); and cDNA (2 μL). Cycling conditions were 94°C for 1 minute; 30 cycles of 94°C for 15 seconds, 56°C for 30 seconds, and 72°C for 30 seconds; the final extension was at 72°C for 7 minutes. PCR products were ligated into pFUSE-hIgG2-Fc2 (InvivoGen) via EcoRI and BglII (New England Biolabs), transformed into *E*. *coli* DH5α (AngYuBio), and selected in LB medium (Sangon Biotech) with 25 μg/mL Zeocin (MKBio). Plasmids were verified by Sanger sequencing.

### Generation of Dectin-1/2-Fc fusion proteins.

HEK293T cells were cultured in 5% low-IgG FCS and transfected at approximately 70% confluence with 60 μg pFUSE-hIgG2-Dectin-1/2-Fc plasmid and 180 μL polyethylenimine (1 mg/mL; Beyotime). After incubation at room temperature for 30 minutes, the mixture was added to cells. Supernatants were collected upon cell death, and Fc fusion proteins were purified using Protein A–Sepharose (BioVision) on a Bio-Rad system.

### Isolation of fungi using Dectin-1/2-Fc fusion proteins.

Mouse feces were homogenized in PBS, centrifuged at 13,400 g for 5 minutes at 4°C, and washed once with TSA (tris-buffered saline and 5% bovine serum albumin) buffer. Samples were incubated with Dectin-1/2-Fc fusion proteins (5 mg/mL) for 1 hour at 4°C, washed, and incubated with biotin anti–Fc-hIgG (5 mg/mL; ab97223, Abcam) for 30 minutes, followed by anti-biotin beads (1 × 10^7^ cells/mL; BioLegend) and EasySep magnetic separation. The procedure was repeated to enrich Fc-positive fungi. Positively sorted cells were subjected to ITS sequencing and cultured on PDA (Solarbio) at 25°C. Monoclonal colonies were expanded in potato dextrose broth (PDB) medium (Solarbio) and identified by sequencing.

### Identification of isolated fungal strains by DNA-Seq.

Fungi cultured in PDB were boiled for 10 minutes, and supernatants were used as PCR templates. ITS regions were amplified using ITS1 (5′-TCCGTAGGTGAACCTGCGG-3′) and ITS4 (5′-TCCTCCGCTTATTGATATGC-3′) primers in a 30 μL KOD FX Neo-based reaction (Toyobo). PCR conditions were 94°C for 3 minutes; 30 cycles of 94°C for 30 seconds, 55°C for 60 seconds, and 72°C for 2 minutes; final extension at 72°C for 10 minutes. Nuclease-free water served as a negative control. PCR products were sequenced and aligned using NCBI BLAST.

### Engyodontium culture and colonization.

Identified *Engyodontium* sp. was cultured on PDA plates at approximately 25°C for 48 hours. Two to 3 colonies were transferred into PDB medium and incubated for 72 hours with shaking. Fungal cells were harvested at 1,500*g* for 10 minutes, washed twice, and stored at –80°C in PDB with 20% glycerol. CFUs were determined by PDA plating. For intestinal colonization, fungi were washed with ice-cold PBS and administered by oral gavage (5 × 10^8^ CFU/mouse). Morphology was confirmed by lactophenol cotton blue staining (DM0080, Leagene).

### Whole-genome sequencing of L.j. WXY.

Genomic DNA from *L.j*. WXY was randomly fragmented, end-repaired, A-tailed, and ligated with Illumina adapters. Adapter-ligated fragments were size selected, PCR-amplified, and purified. Library quality and quantity were assessed by Qubit, real-time PCR, and bioanalyzer-based size distribution analysis. Qualified libraries were pooled and sequenced on the Illumina NovaSeq PE150 platform. Raw reads were filtered to remove: (a) reads with more than 40% bases having quality scores of not higher than 20; (b) reads containing greater than 10% ambiguous bases (N); (c) reads with adapter contamination (>15 bp overlap with <3 mismatches); and (d) reads derived from potential host contamination. Clean reads were assembled using SOAPdenovo with multiple K-mers (95, 107, 119), and the optimal assembly was selected based on scaffold number and assembly quality, followed by parameter optimization (−d, −u, −R, −F). Gaps were filled, low-depth reads (<0.35× average depth) and contigs <500 bp were removed, and the final assembly was used for gene prediction and functional annotation based on the KEGG database.

### Determination of glutamic acid content in Lactobacillus supernatants.

Glutamic acid concentrations in bacterial supernatants were measured using a Glu Content Assay Kit (Beijing Boxbio Science & Technology Co.). *L.j*. WXY, *L*. *reuteri* (NBRC15892), *L*. *animalis* (NBRC15882), *L*. *salivarius* (NBRC102160), *L*. *murinus* (NBRC14221), *L*. *casei* (NBRC15883), *L*. *johnsonii* (NBRC13952), and *L*. *gasseri* (LG21) were cultured in MRS medium at 37°C under anaerobic conditions. Cultures were centrifuged at 13,400*g* for 2 minutes at 4°C, and 40 μL of supernatant was mixed with 170 μL reaction mixture in a 96-well UV plate. Absorbance at 450 nm was measured using a Thermo Fisher Scientific Varioskan LUX reader.

### ELISA analysis.

Levels of IL-6, TNF-α, and IL-1β in tissue homogenates and S100A8/S100A9 in fecal samples were quantified using the following ELISA kits: IL-1β (432601, BioLegend), IL-6 (EM0004, HUABIO), TNF-α (EM0010, HUABIO), and S100A8/S100A9 (WJM-13029M1, JINGMEI). Samples were diluted 50- to 100-fold in PBS, and 100 μL was used per assay. Absorbance at 450 nm was recorded using a Thermo Fisher Scientific Varioskan LUX microplate reader.

### Immunofluorescent staining and analysis.

FFPE tissue sections were subjected to antigen retrieval with citric acid–based solution (G1201, Servicebio), blocked with 5% BSA (232100, Sigma-Aldrich) in PBST for 1 hour, and incubated overnight at 4°C with primary Abs against CXCL2 (ab317569, Abcam), Dectin-2 (GTX41453, GeneTex), and Ly6G (0809-11, HUABIO). After PBST washes, sections were incubated with fluorescent secondary Abs for 1 hour at room temperature and counterstained with DAPI (S2110, Solarbio). Images were acquired using a Leica DM6B fluorescence microscope.

### Statistics.

Statistical analyses for comparisons between 2 groups following the normal distribution were performed using a 2-tailed unpaired Student’s *t* test and between 2 statuses of the same individuals using a paired Student’s *t* test. For experiments involving more than 2 groups, 1-way ANOVA with Tukey’s multiple-comparison test was used. For curves of body weight loss and DAI, 2-way ANOVA with repeated measures was used for comparisons between 2 groups, and 1-way ANOVA with Bonferroni’s multiple-comparison test was used for more than 2 experimental groups. For the data obtained from patients that do not follow a Gaussian distribution (confirmed by Shapiro-Wilk normality test), nonparametric statistic test was performed (between 2 groups, Mann-Whitney test; for more than 3 groups, Kruskal-Wallis and Dunn’s test). Spearman’s correlation test was used to analyze correlations, performed using R software (version 4.1.3). Differences with a *P* value of less than 0.05 were considered statistically significant.

### Study approval.

Written informed consent was obtained from all participants prior to sample collection. All procedures were approved by the Committee for Clinical Investigation of the First Affiliated Hospital, Sun Yat-sen University (approval IIT-2021-654, 2019-55) and conducted in accordance with institutional guidelines. All animal experiments were approved by the Experimental Animal Management and Use Committee of Sun Yat-sen University (approvals 2020000113 and 2021001577).

### Data availability.

Raw sequencing files of bacterial 16S rDNA-Seq have been deposited in the NCBI GEO (http://www.ncbi.nlm.nih.gov/geo/; GSE274388 and PRJNA1393726). Fungal ITS sequencing and raw sequencing files of the whole genome of *L.j*. WXY have been deposited in Assembly and Biosample (https://www.ncbi.nlm.nih.gov/assembly/; Bioproject: PRJNA1146165, PRJNA1146185, PRJNA1048685). The untargeted metabolomic raw and processed data have been deposited in NIH Common Fund’s National Metabolomics Data Repository (https://www.metabolomicsworkbench.org/data/; DATATRACK_ID: 5090). The publicly available data of scRNA-Seq and bulk RNA-Seq used in this study are in GEO database under accession codes GSE168033, GSE117993, and GSE202052. All other relevant data supporting the findings of this study (including Supporting Data Values file) are available within the article, in supplemental material, and in source data.

## Author contributions

X Wang, HS, and YT primarily contributed to the work and wrote the manuscript. Their order as co–first authors was determined by the volume of work each performed in the study. ZL analyzed the clinical data of patients with IBD. SX assisted with the analysis of 16S rDNA-Seq and scRNA-Seq data. YT helped analyze metabolite data and performed IHC staining. DQ, KJ, JD, and BF assisted with in vivo mouse experiments. CH collected and pretreated human colon tissue samples. Under MC’s supervision, X Wu and RF collected stools and colon tissues from patients with CD and healthy individuals and analyzed gene transcripts, fecal metabolites, and 16S rDNA-Seq. CT generated all gene-mutant mice for this study, with YI providing advice for mutant mouse generation. CT organized and supervised the project and edited the manuscript with RF, MC, and CH.

## Conflict of interest

The authors have declared that no conflict of interest exists.

## Funding support

- Prevention and Control of Emerging and Major Infectious Diseases – National Science and Technology Major Project (grant 2025ZD01903900 to CT).
- General Program of the National Natural Science Foundation of China (grants 82370540 and 82070564 to CT).
- General Program of the National Natural Science Foundation of Guangdong Province, China (grant 2024A1515013236 to CT).
- The Foundation of 100 Talents Program of Sun Yat-Sen University (Y61231 to CT).
- The Fundamental Research Funds for the Central Universities, Sun Yat-sen University (to CT), The Grants-in-Aid from the Ministry of Education, Culture, Sports, Science and Technology of Japan, Scientific Research (B) (20H03176 to CT).
- The Youth Program of the National Natural Science Foundation of China (grant 82403250 to HS).

## Supplementary Material

Supplemental data

Supporting data values

## Version 1 03/02/2026

In-Press Preview

## Version 2 04/15/2026

Electronic publication
