# Supplementary material for: Colonic Engyodontium fungus triggers neutrophil antimicrobial activity to suppress Lactobacillus johnsonii–derived glutamic acid–maintained Tregs
Source: J Clin Invest. 2026 Feb 17;136(8):e196788. doi: 10.1172/JCI196788 (PMC13078882; doi:10.1172/JCI196788)
Supplement: Supplemental data [file jci-136-196788-s190.pdf]

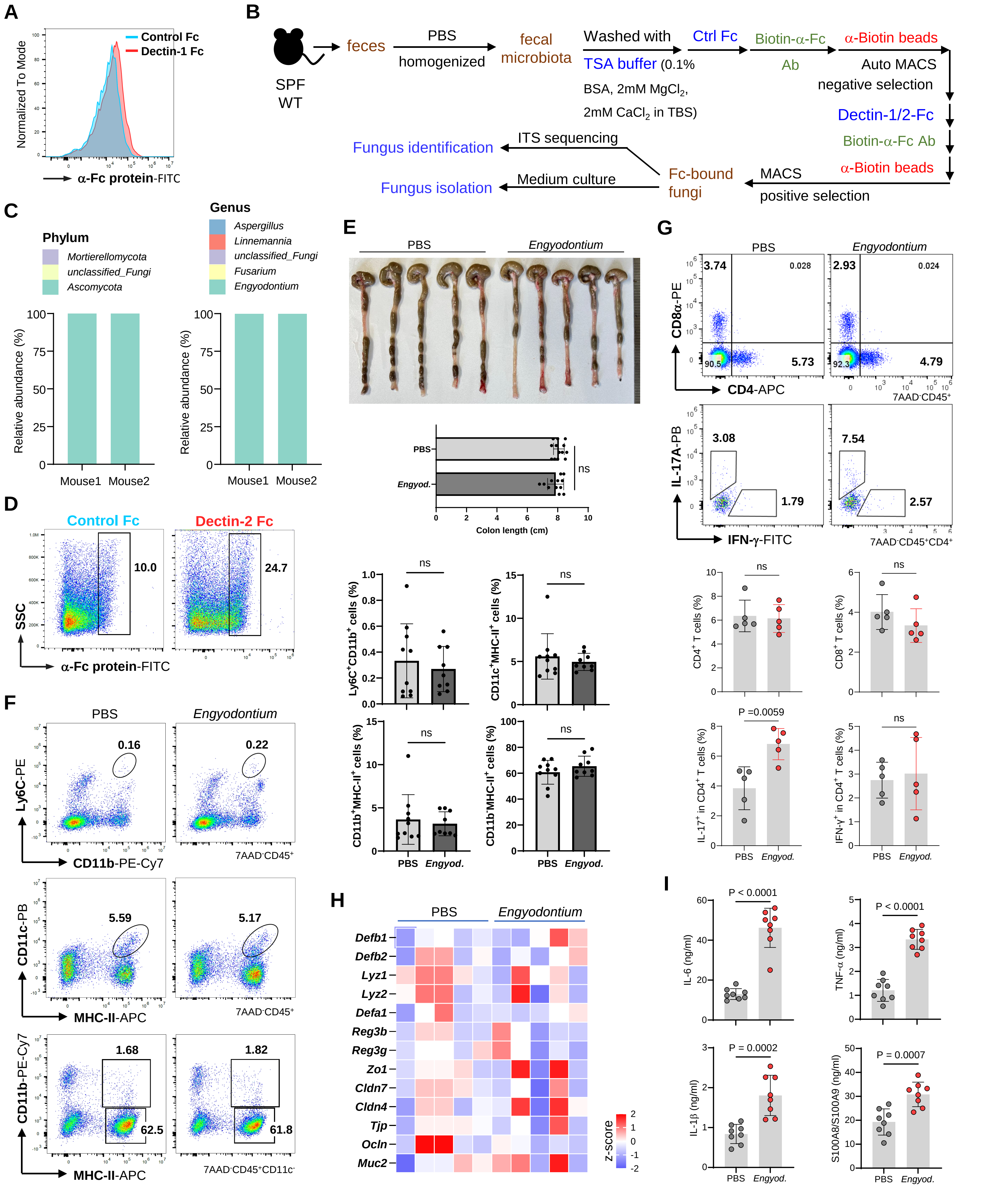
A
Control Fc
Dectin-1 Fc
Normalized To Mode
α-Fc protein-FITC
B
SPF
WT
feces
PBS
homogenized
fecal
microbiota
Washed with
TSA buffer (0.1%
BSA, 2mM MgCl2,
2mM CaCl2 in TBS)
Ctrl Fc
Biotin-α-Fc
Ab
α-Biotin beads
Auto MACS
negative selection
Dectin-1/2-Fc
Biotin-α-Fc Ab
α-Biotin beads
MACS
positive selection
Fc-bound
fungi
ITS sequencing
Fungus identification
Medium culture
Fungus isolation
C
Phylum
Mortierellomycota
unclassified_Fungi
Ascomycota
Genus
Aspergillus
Linnemannia
unclassified_Fungi
Fusarium
Engyodontium
Relative abundance (%)
Mouse1
Mouse2
D
Control Fc
Dectin-2 Fc
10.0
24.7
SSC
α-Fc protein-FITC
E
PBS
Engyodontium
PBS
Engyod.
ns
Colon length (cm)
Ly6C+CD11b+ cells (%)
CD11c+MHC-II+ cells (%)
CD11b+MHC-II+ cells (%)
CD11b-MHC-II+ cells (%)
F
PBS
Engyodontium
0.16
0.22
Ly6C-PE
CD11b-PE-Cy7
7AAD-CD45+
5.59
5.17
CD11c-PB
MHC-II-APC
1.68
1.82
62.5
61.8
CD11b-PE-Cy7
7AAD-CD45+CD11c-
G
PBS
Engyodontium
3.74
0.028
2.93
0.024
90.5
5.73
92.3
4.79
CD8α-PE
CD4-APC
7AAD-CD45+
3.08
1.79
7.54
2.57
IL-17A-PB
IFN-γ-FITC
7AAD-CD45+CD4+
CD4+ T cells (%)
CD8+ T cells (%)
P =0.0059
IL-17+ in CD4+ T cells (%)
IFN-γ+ in CD4+ T cells (%)
H
PBS
Engyodontium
Defb1
Defb2
Lyz1
Lyz2
Defa1
Reg3b
Reg3g
Zo1
Cldn7
Cldn4
Tjp
Ocln
Muc2
z-score
I
P < 0.0001
IL-6 (ng/ml)
P < 0.0001
TNF-α (ng/ml)
P = 0.0002
IL-1β (ng/ml)
P = 0.0007
S100A8/S100A9 (ng/ml)
PBS
Engyod.

Supplementary Figure 1. ***Engyodontium*** **colonization impacts intestinal neutrophils and Treg cells at steady state.**

(A) Flow cytometry analysis of mouse fecal commensal microbiome binding to Dectin-1-Fc protein.

(B) Protocol for intestinal fungus isolation and identification.

(C) Phylum and genus composition of mouse fecal commensal fungi bound by Dectin-1-Fc, identified by ITS1-2 sequencing.

(D) FACS analysis of Dectin-2-Fc-positive fecal microorganisms.

(E-I) In parallel with the experiment described in Figure 1I-1L, the following were evaluated: **(E)** gross colonic appearance and colon length (WT n=13, *Clec4n*$^{-/-}$ n=12), **(F)** proportions of monocytes (Ly6C^{+}CD11b^{+}), dendritic cells (MHC-II^{+}CD11c^{+}), M1 macrophages (MHC-II^{+}CD11b^{+} in CD11c^{-} cells), B cells (MHC-II^{+}CD11b^{-} in CD11c^{-} cells), **(G)** IL-17^{+} CD4^{+} T cells, and IFN-γ^{+}CD4^{+} T cells by FACS analysis (WT n=10, *Clec4n*$^{-/-}$ n=9), **(H)** mRNA expression of antimicrobial peptides and epithelial tight junction genes in colon tissues by qPCR (heatmap) (n=5/group), and **(I)** protein levels of IL-6, TNF-α, IL-1β and S100A8/9 calprotectin in the colon tissue lysates by ELISA (n=8/group).

Data in panels (**E-G**) are pooled from three and in (**I**) are pooled from two independent experiments. Data in panels (**E-G**, **I**) are shown as mean ± SD. Statistical analysis: two-tailed unpaired Student’s t-test (**E-I**).

Supplementary Figure 2

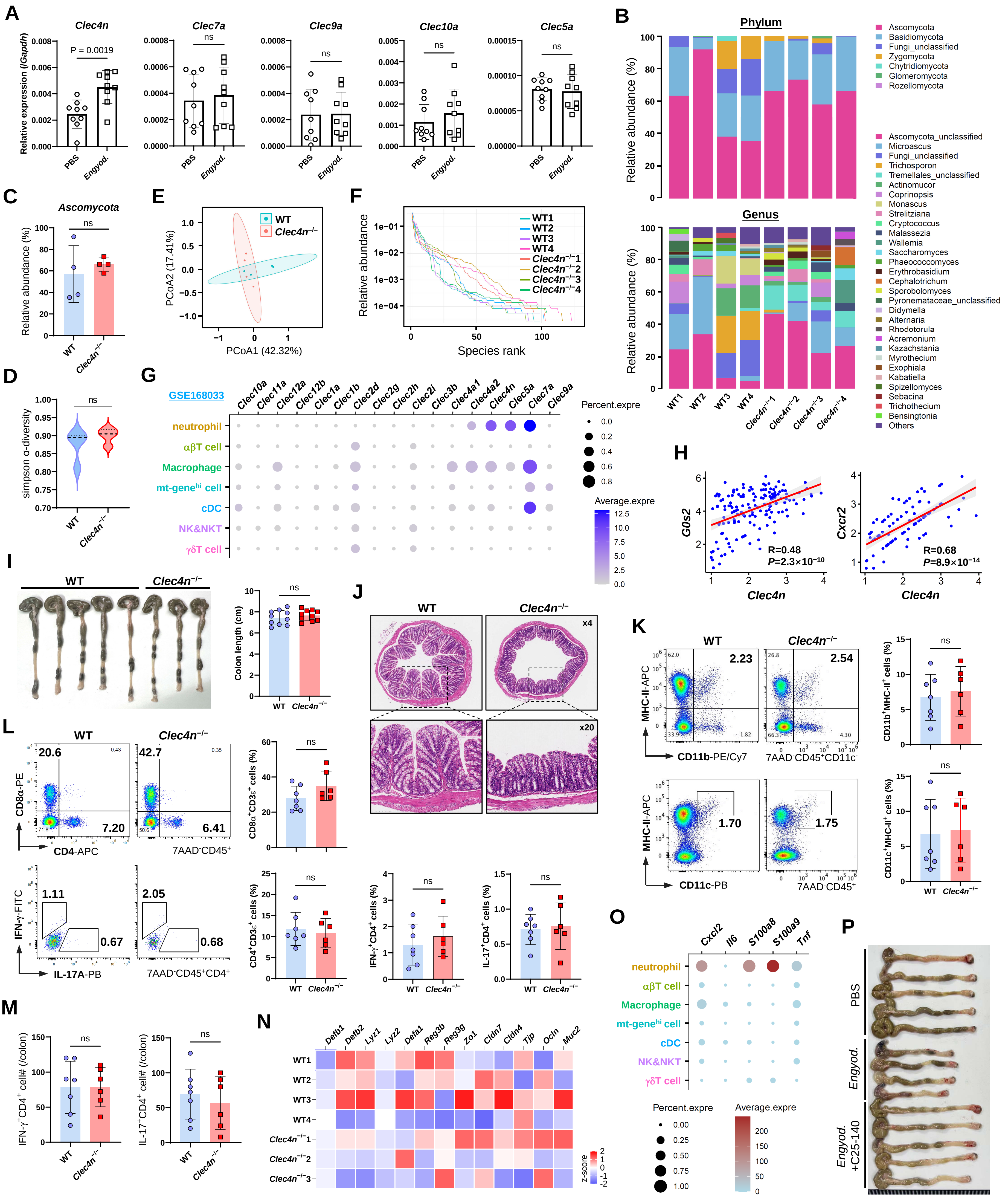

Supplementary Figure 2. ***Engyodontium*** **colonization up-regulates Dectin-2 expression in mouse colon, while Dectin-2 deficiency rarely affects most colonic immune cell populations.**

(A) In parallel with the experiment described in Figure 1I-1L, the mRNA expression levels of genes encoding CLRs in colon tissues were measured by qPCR (n=9 /group).

(B-F) The relative abundance of the entire intestinal fungal community **(B)** and *Ascomycota* **(C)**, α-diversity **(D)**, β-diversity (PCoA, **E**) and the species rank of the intestinal fungi **(F)** in WT and *Clec4n*$^{-/-}$ mouse feces were assessed using fungal ITS1-2 sequencing analysis (n=4 /group).

(G) Dot plots illustrating the proportion of cells expressing specific CLR-encoding genes and the average expression level of each gene within distinct cell clusters of colonic immune cells from C57BL/6 mice under steady-state conditions, derived from the reanalysis of a public single-cell RNA-sequencing (scRNA-seq) dataset (GSE168033).

(H) Point plot depicting the correlation of expression levels between *Clec4n* and *G0S2* or *Cxcr2* in cells double-positive for *Clec4n* and *G0S2* or *Cxcr2*, using scRNA-seq datasets (GSE168033).

(I-N) In parallel with the experiment described in Figure 2D-2F, the following were evaluated: **(I)** gross colonic appearance and colon length (WT n=10, *Clec4n*$^{-/-}$ n=9), **(J)** histological analyses of the distal colon (H&E staining), **(K)** proportions of dendritic cells (MHC-II^{+}CD11c^{+}) and M1 macrophages (MHC-II^{+}CD11b^{+}CD11c^{-}) (WT n=7, *Clec4n*$^{-/-}$ n=6), (**L**, **M**) proportions of CD8^{+} and CD4^{+} T cells, proportions and absolute numbers of IL-17^{+}CD4^{+} T cells and IFN-γ^{+}CD4^{+} T cells by FACS analysis (WT n=7, *Clec4n*$^{-/-}$ n=6), and **(N)** mRNA expression of antimicrobial peptides and epithelial tight junction genes in colon tissues by qPCR, presented in a heatmap (WT n=4, *Clec4n*$^{-/-}$ n=3).

(O) Dot plots showing the proportion of cells expressing indicated genes and the average expression level of each gene within distinct cell clusters of colonic immune cells from C57BL/6 mice under steady-state conditions, derived from the reanalysis of the scRNA-seq dataset GSE168033.

(P) In parallel with the experiment described in Figure 2I-2L, gross colonic appearance in the indicated groups is exhibited.

Data in panels (**A**, **C**, **I**, **K-M**) are pooled from two independent experiments and are presented as mean ± SD. Statistical analysis: two-tailed unpaired Student's t-test (**A**, **C**, **D**, **I**, **K-N**).

Supplementary Figure 3

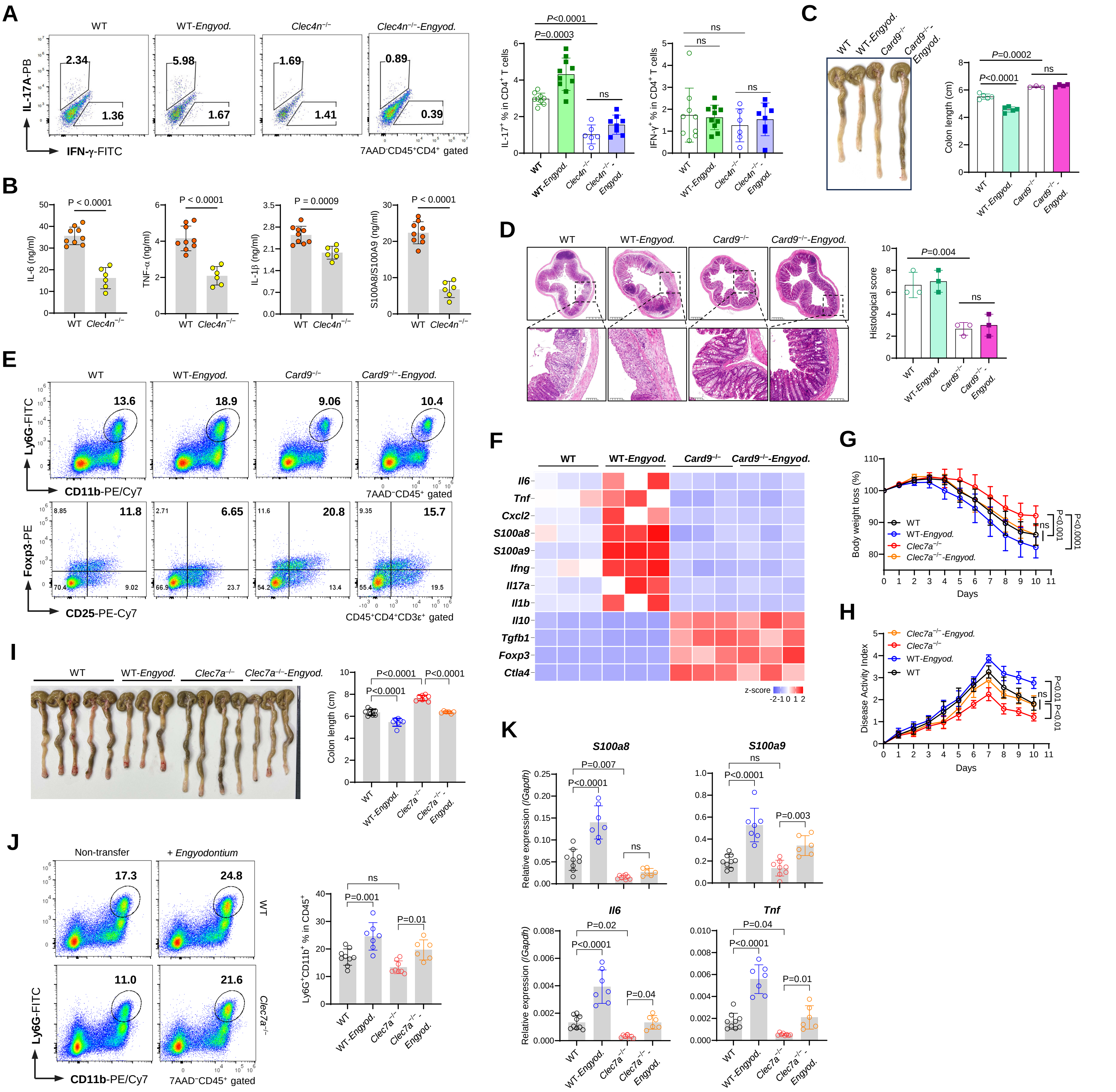

Supplementary Figure 3. *Engyodontium* exacerbates colitis primarily through Dectin-2–CARD9 signaling, not Dectin-1.

(A) In parallel with the experiment described in Figure 3E, IFN-γ/IL-17-producing $CD4^+$ T cells were quantified using FACS (WT n=9, WT-*Engyodontium* n=10, *Clec4n*$^{-/-}$ n=6, *Clec4n*$^{-/-}$-*Engyodontium* n=8).

(B) In parallel with the experiment described in Figure 3G, protein levels of IL-6, TNF-α, IL-1β and S100A8/9 calprotectin in the colon tissue lysates from WT and *Clec4n*$^{-/-}$ mice were examined by ELISA (WT n=9, *Clec4n*$^{-/-}$ n=6).

(C-F) In parallel with the experiment described in Figure 3H-3J, on day 10 post-DSS treatment, mice were sacrificed, and gross colon observations, colon length measurements **(C)**, and histological analyses of the distal colon (H&E staining) **(D)** were conducted. Following leukocyte isolation from the cLP, the proportions of neutrophils ($Ly6G^+CD11b^+$ in $CD45^+$ cells) and Tregs ($CD25^+Foxp3^+$ in $CD4^+$ T cells) were quantified using FACS **(E)**. The mRNA expression levels of immune-related genes in colon tissues were determined by qPCR and presented in a heatmap **(F)** (**C**, **E**, WT groups n=5, *Card9*$^{-/-}$ group n=3, *Card9*$^{-/-}$-*Engyodontium* group n=4; **D**, **F**, n=3/group).

(G-K) WT and *Clec7a*$^{-/-}$ mice were orally administrated with PBS or *Engyodontium* sp. every other day for three times, followed by administration of 1.5% DSS in drinking water from the second fungal colonization for 7 days. Body weight loss **(G)** and disease activity index **(H)** were monitored daily during colitis induction. On day 10 post-DSS treatment, mice were sacrificed, and gross colon observations and colon length measurements **(I)** were conducted. Following leukocyte isolation from the cLP, the proportions of neutrophils ($Ly6G^+CD11b^+$ in $CD45^+$ cells) were quantified using FACS **(J)** (WT n=9, WT-*Engyodontium* n=7, *Clec7a*$^{-/-}$ n=8, *Clec7a*$^{-/-}$-*Engyodontium* n=6). The mRNA expression levels of indicated genes encoding cytokines and calprotectin in colon tissues were determined by qPCR **(K)** (WT n=9, WT-*Engyodontium* n=7, *Clec7a*$^{-/-}$ n=8, *Clec7a*$^{-/-}$-*Engyodontium* group n=6).

Data in panels (**A**, **B**, **G-K**) are pooled from two independent experiments. Data in panels (**A -D**, **G-K**) are presented as mean ± SD. Statistical analysis: one-way ANOVA with Tukey's multiple comparisons test (**A**, **C**, **D**, **F-K**), two-tailed unpaired Student's t-test (**B**).

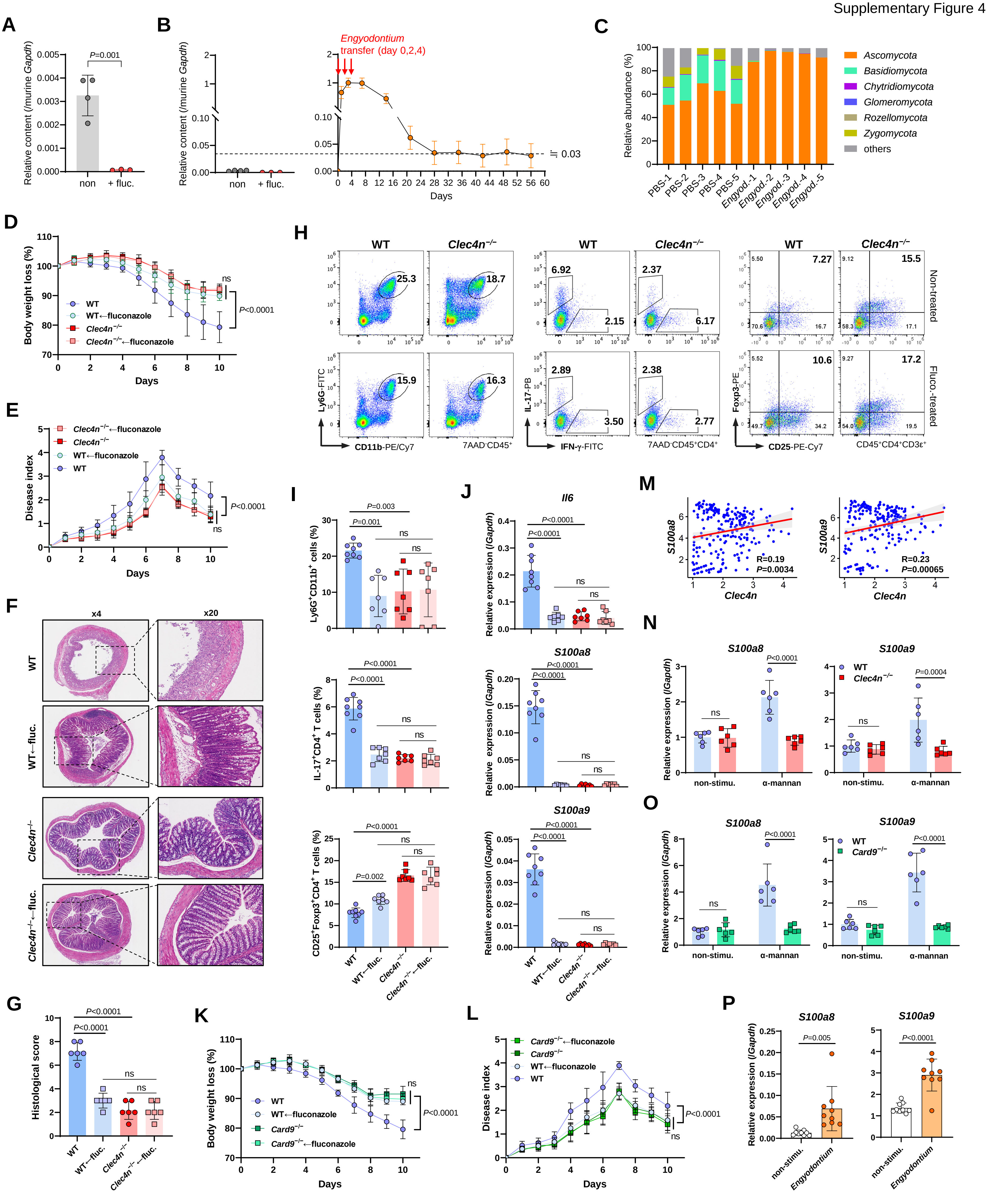
A
B
C
D
E
F
G
H
I
J
K
L
M
N
O
P
Engyodontium transfer (day 0,2,4)
Relative content (/murine Gapdh)
Relative abundance (%)
Body weight loss (%)
Disease index
Histological score
Days
WT
WT←fluconazole
Clec4n−/−
Clec4n−/−←fluconazole
Card9−/−
Card9−/−←fluconazole
Ascomycota
Basidiomycota
Chytridiomycota
Glomeromycota
Rozellomycota
Zygomycota
others
P<0.0001
ns
Non-treated
Fluco.-treated
CD11b-PE/Cy7
Ly6G-FITC
IFN-γ-FITC
IL-17-PB
CD25-PE-Cy7
Foxp3-PE
Il6
S100a8
S100a9
Clec4n
R=0.19
P=0.0034
R=0.23
P=0.00065
non-stimu.
α-mannan
Engyodontium

Supplementary Figure 4. Intestinal commensal fungi aggravate colitis through the Dectin-2-CARD9 pathway.

(A, **B)** C57BL/6 mice were treated with fluconazole for 1 week, and were then administrated with 5×10^8 CFU *Engyodontium* sp. every other day for 3 doses. Mouse feces were then harvested once a week until Day 56 after the initial *Engyodontium* colonization, and relative content of *Engyodontium* was examined by qPCR. **(A)** The content of *Engyodontium* in the feces of normal mice and mice pretreated with fluconazole (murine *Gapdh* as reference control). **(B)** Kinetics of *Engyodontium* content in mouse feces during and after the administration (n=3-4 /group).

(C) C57BL/6 mice were administrated with 5×10^8 CFU *Engyodontium* sp. every other day for 3 doses, and on Day 3 after the last administration, fecal DNA was isolated and relative abundance of commensal fungi was determined by qPCR (n=5 /group).

(**D-J**) WT and *Clec4n*$^{-/-}$ mice were administered fluconazole for 2 weeks, followed by 1.5% DSS in drinking water for 7 days. Body weight loss **(D)** and disease activity index **(E)** were evaluated daily. On day 10 post-DSS treatment, mice were sacrificed. Histological analyses of the distal colon (H&E staining) **(F, G)** were conducted. Leukocytes from the cLP were harvested, and the proportions of neutrophils (Ly6G$^+$CD11b$^+$ in CD45$^+$ cells), IL-17$^+$CD4$^+$ T cells, and Tregs (CD25$^+$Foxp3$^+$ in CD4$^+$ T cells) were determined by FACS **(H**, **I)**. The mRNA expression levels of *Il6*, *S100a8*, and *S100a9* in colon tissues were assessed by qPCR **(J)**. (**D-F**, **H-J**, WT n=8, WT fluconazole treated n=7, *Clec4n*$^{-/-}$ n=7, *Clec4n*$^{-/-}$ fluconazole n=7; **G**, n=6 /group).

(**K**, **L**) WT and *Card9*$^{-/-}$ mice were administered fluconazole for 2 weeks, followed by 1.5% DSS in drinking water for 7 days. Body weight loss **(K)** and disease activity index **(L)** were evaluated daily (WT n=8, WT fluconazole treated n=7, *Card9*$^{-/-}$ n=8, *Card9*$^{-/-}$ fluconazole n=9).

(**M**) Correlation of expression levels between *Clec4n* and *S100a8* or *S100a9* in *Clec4n* and *S100a8*/*S100a9* double-positive cells, analyzed from pooled scRNA-seq datasets (GSE168033) derived from mouse cLP cells on Day 0 to 42 post-DSS treatment.

(**N**, **O**) Bone marrow-derived neutrophils from WT and *Clec4n*$^{-/-}$ mice **(N)** or *Card9*$^{-/-}$ mice **(O)** were induced in vitro and stimulated with α-mannan for 24 hours, followed by qPCR analysis of *S100a8* and *S100a9* expression (n=6 technical replicates/group).

(**P**) C57BL/6 mice were administered 1.5% DSS for 7 days. On day 10 after DSS treatment, mouse colon tissues were obtained, and cLP CD11b$^+$ cells were isolated and stimulated with *Engyodontium* sp. for 24 hours. The relative expression of *S100a8* and *S100a9* in harvested cells after culture was determined by qPCR (n=9 /group).

Data in panels (**D-L, N-P**) are pooled from two independent experiments. Data in panels (**A**, **B**, **D-G**, **I-L**, **N-P**) are presented as mean ± SD. Statistical analysis: two-tailed unpaired Student's t-test (**A**, **B**, **N**, **P**), one-way ANOVA test with Bonferroni's multiple comparisons: (**D**, **E**, **K**, **L**), one-way ANOVA with Tukey's multiple comparisons test (**F**, **I**, **J**), Spearman's correlation test (**M**).

Supplementary Figure 5

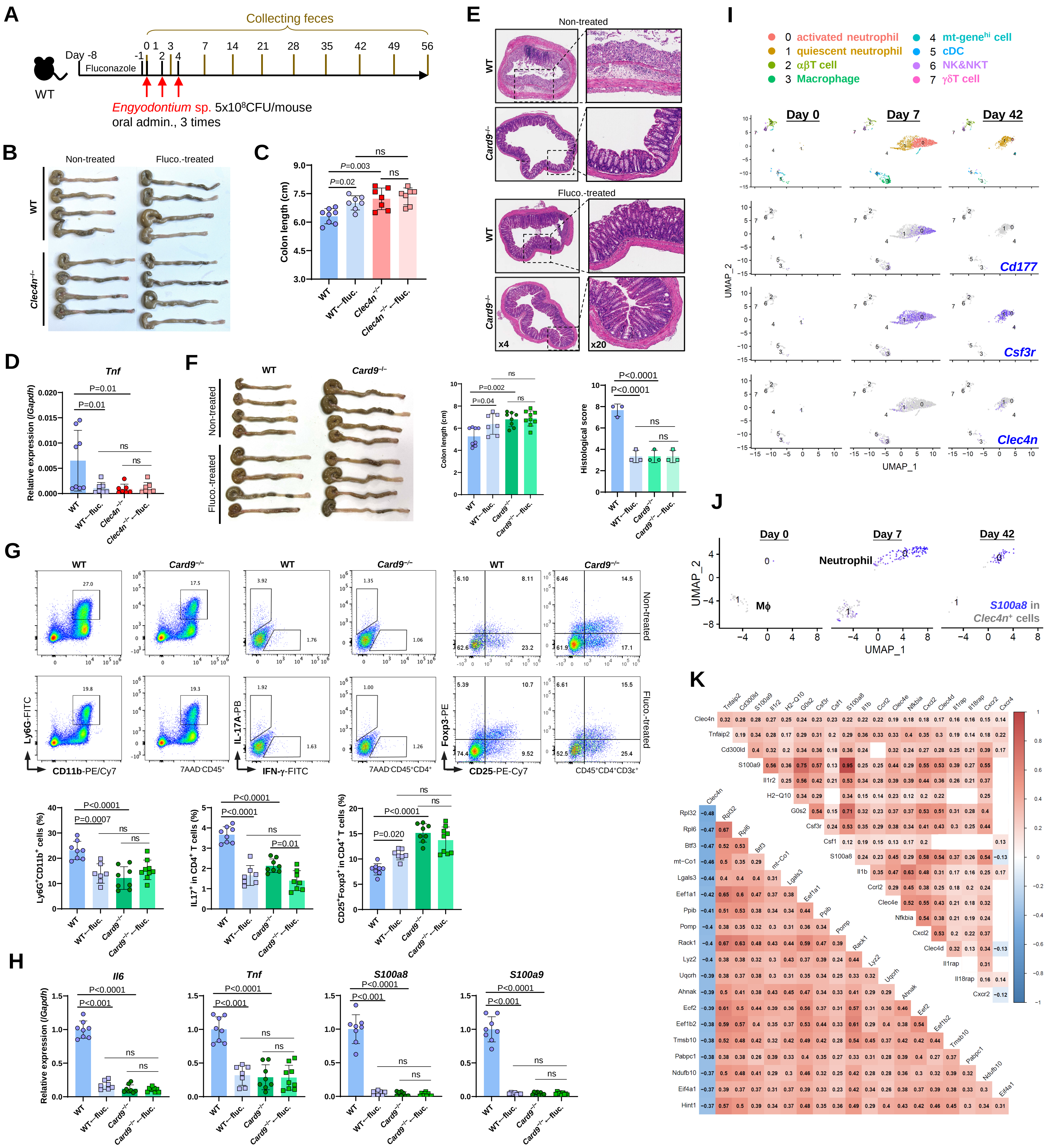

Supplementary Figure 5. Intestinal commensal fungi exacerbate colitis in a Dectin-2-CARD9 signaling dependent manner

(A) The protocol of the experiment described in Supplementary Figure 4A-B.

(B-D) In parallel with the experiment described in Supplementary Figure 4D-J, the following were evaluated: **(B)** gross colonic appearance, **(C)** colon length measurement, and **(D)** mRNA expression of *Tnf* in colon tissues by qPCR (**D**, WT n=8, WT fluconazole treated n=7, *Clec4n*$^{-/-}$ n=7, *Clec4n*$^{-/-}$ fluconazole n=7).

(E-H) In parallel with the experiment described in Supplementary Figure 4K-L, the following were evaluated: **(E)** histological analyses of the distal colon (H&E staining), **(F)** gross colon observations and colon length. **(G)** Leukocytes from the cLP were harvested, and the proportions of neutrophils (Ly6G$^+$CD11b$^+$ in CD45$^+$ cells), IL-17$^+$CD4$^+$ T cells, and Tregs (CD25$^+$Foxp3$^+$ in CD4$^+$ T cells) were determined by FACS. **(H)** The mRNA expression levels of *Il6*, *S100a8*, and *S100a9* in colon tissues were assessed by qPCR (**F-H**, WT n=8, WT fluconazole n=7, *Card9*$^{-/-}$ n=8, *Card9*$^{-/-}$ fluconazole n=9; **E**, n=3/group).

(I-K) UMAPs illustrating the distribution of cells expressing indicated genes in each cell cluster of colonic immune cells **(I)**, and the distribution of *S100a8* expression in each *Clec4n*$^+$ colonic cell cluster **(J)** before and after 7 days of DSS treatment, as well as after 35 days of recovery (Day 42), by reanalyzing the scRNA-seq dataset (GSE168033). **(K)** Heatmap showing the correlation among the indicated gene expressions in *Clec4n*$^+$ colonic cells using Spearman's correlation analysis.

Data in panels (**D**, **F-H**) are pooled from two independent experiments, and in panel (**B**, **C**) are from one of two independent experiments. Data in panels (**D-H**) and are presented as mean ± SD. Statistical analysis: one-way ANOVA with Tukey's multiple comparisons test (**D-H**), Spearman's correlation test (**K**).

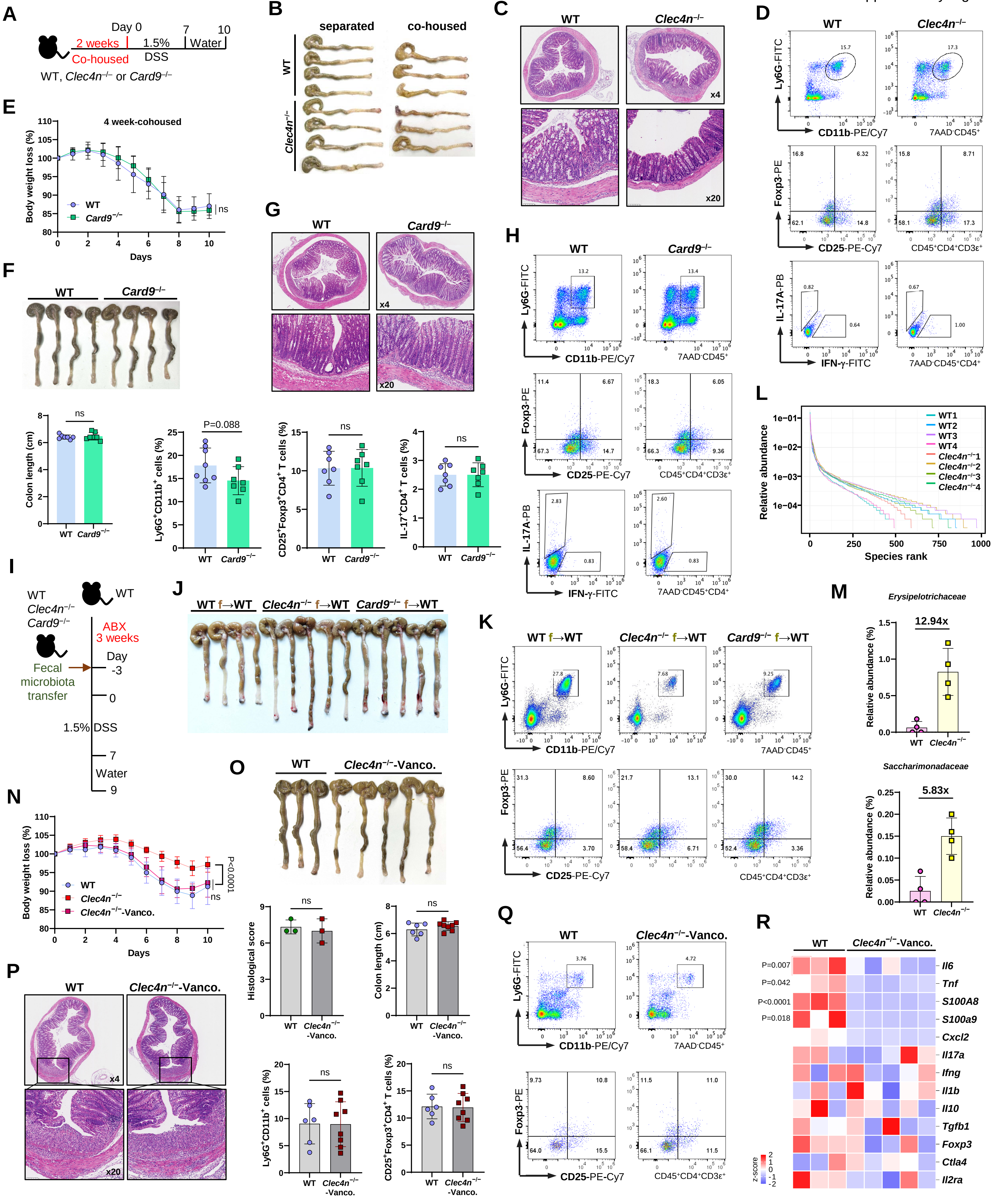
A
Day 0
7
10
2 weeks
1.5%
Water
Co-housed
DSS
WT, Clec4n−/− or Card9−/−
B
separated
co-housed
WT
Clec4n−/−
C
WT
Clec4n−/−
x4
x20
D
WT
Clec4n−/−
Ly6G-FITC
CD11b-PE/Cy7
7AAD-CD45+
15.7
17.3
Foxp3-PE
CD25-PE-Cy7
CD45+CD4+CD3ε+
IL-17A-PB
IFN-γ-FITC
7AAD-CD45+CD4+
E
4 week-cohoused
Body weight loss (%)
Days
WT
Card9−/−
ns
F
WT
Card9−/−
Colon length (cm)
Ly6G+CD11b+ cells (%)
P=0.088
CD25+Foxp3+CD4+ T cells (%)
IL-17+CD4+ T cells (%)
ns
G
WT
Card9−/−
H
WT
Card9−/−
L
Relative abundance
Species rank
WT1
WT2
WT3
WT4
Clec4n−/−1
Clec4n−/−2
Clec4n−/−3
Clec4n−/−4
I
WT
Clec4n−/−
Card9−/−
ABX
3 weeks
Fecal microbiota transfer
Day
-3
0
1.5% DSS
7
Water
9
J
WT f→WT
Clec4n−/− f→WT
Card9−/− f→WT
K
WT f→WT
Clec4n−/− f→WT
Card9−/− f→WT
M
Erysipelotrichaceae
12.94x
Saccharimonadaceae
5.83x
Relative abundance (%)
N
WT
Clec4n−/−
Clec4n−/−-Vanco.
P<0.0001
O
WT
Clec4n−/−-Vanco.
Histological score
Colon length (cm)
Ly6G+CD11b+ cells (%)
CD25+Foxp3+CD4+ T cells (%)
P
WT
Clec4n−/−-Vanco.
Q
WT
Clec4n−/−-Vanco.
R
WT
Clec4n−/−-Vanco.
P=0.007
P=0.042
P<0.0001
P=0.018
Il6
Tnf
S100A8
S100a9
Cxcl2
Il17a
Ifng
Il1b
Il10
Tgfb1
Foxp3
Ctla4
Il2ra
z-score

Supplementary Figure 6. Attenuation of intestinal fungus-promoted colitis in Dectin-2/CARD9 signaling-deficient mice is dependent on commensal microbiota

(A-D) In parallel with the experiment described in Figure 4A-F, the following were evaluated: **(A)** the protocol of the co-housing experiments, **(B)** gross colonic appearance, **(C)** histological analyses of the distal colon (H&E staining) in co-housed groups, and **(D)** proportions of neutrophils ($Ly6G^+CD11b^+$ in $CD45^+$ cells), Treg cells ($CD25^+Foxp3^+$ in $CD4^+$ T cells), IL-17^+/IFN-γ^+CD4^+ T cells in co-housed groups by FACS analysis (**D**, n=6 /co-housed group, WT separated n=10, *Clec4n*$^{-/-}$ separated n=7).

(E-H) In parallel with the experiment described in Figure 4G-I, the following were evaluated: **(E)** daily body weight loss post-DSS treatment, **(F)** gross colonic appearance and colon length, **(G)** histological analysis of the distal colon (H&E staining), and **(H)** proportions of neutrophils ($Ly6G^+CD11b^+$ in $CD45^+$ cells), Treg cells ($CD25^+Foxp3^+$ in $CD4^+$ T cells), and IL-17^+/IFN-γ^+CD4^+ T cells by FACS analysis (**E**, **F**, **H** n=7 /group; **G**, n=5 /group).

(I-K) In parallel with the experiment described in Figure 4J-N, the following were evaluated: **(I)** the protocol of the fecal microbiota-transfer experiments, **(J)** gross colonic appearance, and **(K)** proportions of neutrophils ($Ly6G^+CD11b^+$ in $CD45^+$ cells) and Treg cells ($CD25^+Foxp3^+$ in $CD4^+$ T cells) by FACS analysis (WT-f n=9, *Clec4n*$^{-/-}$-f n=10, *Card9*$^{-/-}$-f n=10).

(**L**, **M**) In parallel with the experiment described in Figure 4O-S, the following were evaluated: **(L)** species rank of the whole fecal microflora, and **(M)** relative abundance of *Erysipelotrichaceae* and *Saccharimonadaceae* in fecal bacteria from WT and *Clec4n*$^{-/-}$ mice, determined by 16S rDNA sequencing analysis (n=4 /group).

(N-R) In parallel with the experiment described in Figure 4T, the following were evaluated: **(N)** daily body weight loss post-DSS treatment, **(O)** gross colonic appearance and colon length, **(P)** histological analysis of the distal colon (H&E staining), **(Q)** proportions of neutrophils ($Ly6G^+CD11b^+$ in $CD45^+$ cells) and Treg cells ($CD25^+Foxp3^+$ in $CD4^+$ T cells) by FACS analysis, and **(R)** mRNA expression of genes encoding cytokines and antimicrobial peptides in colon tissues, shown in a heatmap (WT n=9, *Clec4n*$^{-/-}$ n=8, *Clec4n*$^{-/-}$-Vanco. n=8).

Data in panels (**E**, **F**, **H**, **N**, **O**, **Q**) are pooled from two independent experiments, and in panels (**B**, **C**, **D**, **G**, **J**, **K**, **R**) are from one of two independent experiments. Statistical analysis: two-way ANOVA test with repeated measures (**E**), two-tailed unpaired Student's t-test (**F**, **H**, **O-R**), one-way ANOVA test with Bonferroni's multiple comparisons (**N**). Data in panels (**E**, **F**, **H**, **M-Q**) are presented as mean ± SD.

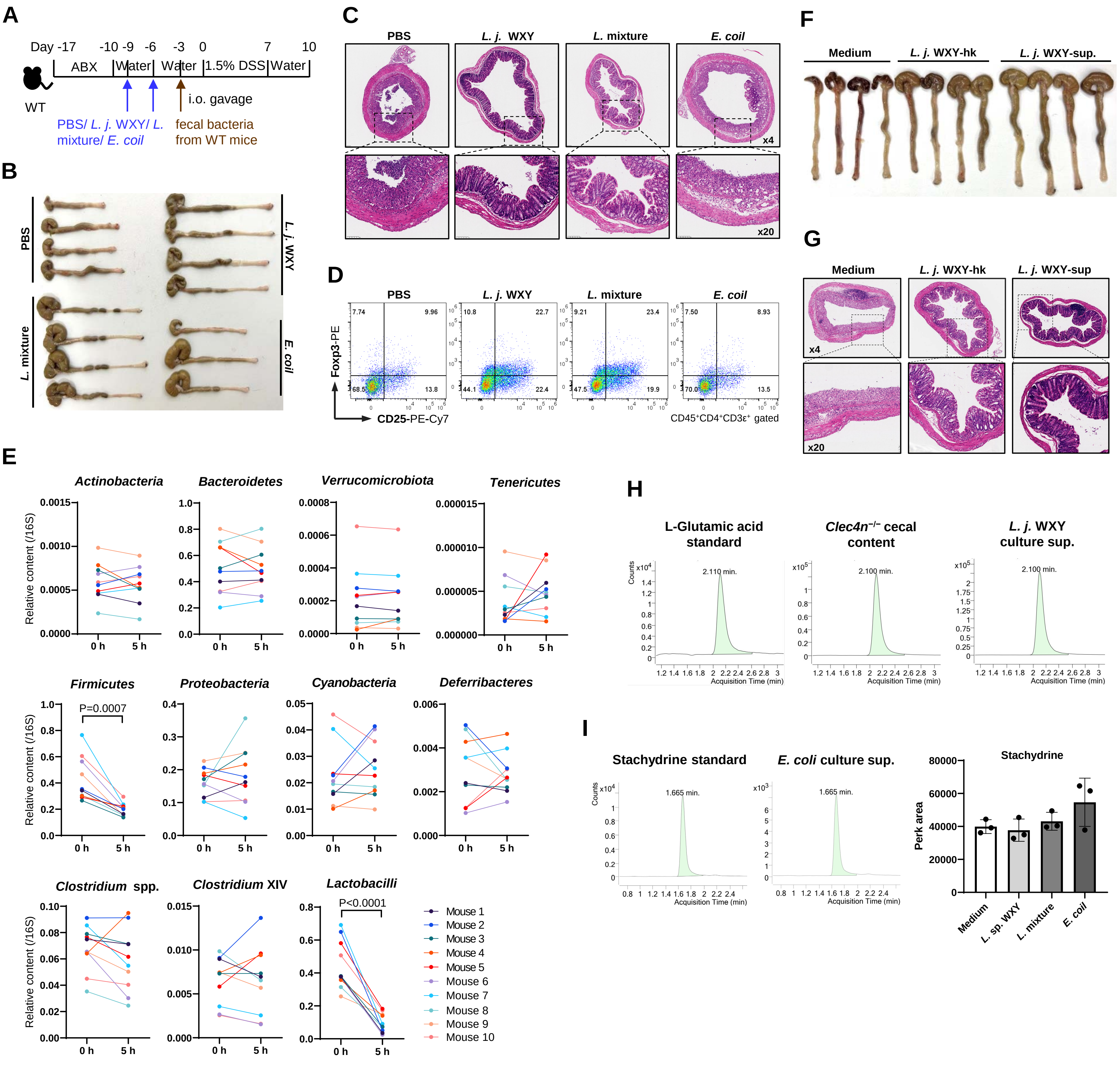
A
Day -17 -10 -9 -6 -3 0 7 10
ABX
Water
Water
1.5% DSS
Water
WT
i.o. gavage
PBS/ L. j. WXY/ L. mixture/ E. coil
fecal bacteria from WT mice
B
PBS
L. j. WXY
L. mixture
E. coil
C
PBS
L. j. WXY
L. mixture
E. coil
x4
x20
D
PBS
L. j. WXY
L. mixture
E. coil
Foxp3-PE
CD25-PE-Cy7
CD45⁺CD4⁺CD3ε⁺ gated
E
Actinobacteria
Bacteroidetes
Verrucomicrobiota
Tenericutes
Firmicutes
P=0.0007
Proteobacteria
Cyanobacteria
Deferribacteres
Clostridium spp.
Clostridium XIV
Lactobacilli
P<0.0001
Relative content (/16S)
0 h
5 h
Mouse 1
Mouse 2
Mouse 3
Mouse 4
Mouse 5
Mouse 6
Mouse 7
Mouse 8
Mouse 9
Mouse 10
F
Medium
L. j. WXY-hk
L. j. WXY-sup.
G
Medium
L. j. WXY-hk
L. j. WXY-sup
x4
x20
H
L-Glutamic acid standard
Clec4n⁻/⁻ cecal content
L. j. WXY culture sup.
2.110 min.
2.100 min.
2.100 min.
Counts
Acquisition Time (min)
I
Stachydrine standard
E. coli culture sup.
1.665 min.
1.665 min.
Stachydrine
Perk area
Medium
L. sp. WXY
L. mixture
E. coil

Supplementary Figure 7. Dectin-2 deficiency-facilitated colonic *Lactobacillus johnsonii* WXY attenuates DSS-colitis

(A-D) In parallel with the experiment described in Figure 5B-F, WT mice were treated with a CEGKMV-mixed antibiotic cocktail in drinking water for 1 week. Subsequently, the indicated bacterial strains were administered orally twice over 7 days, followed by the transfer of the whole fecal microbiota from normal WT mice for 3 days and 1.5% DSS treatment for 7 days **(A)**. On day 10 post-DSS treatment, mice were sacrificed for gross colon observation **(B)** and histological analysis of the distal colon (H&E staining) **(C)**. Proportions of Tregs ($CD25^+Foxp3^+$ in $CD4^+$ T cells) were determined by FACS **(D)** (PBS n=7, *L. j.* WXY n=7, *L.* mixture n=8, *E. coli* n=7).

(E) In parallel with the experiment described in Figure 5I, relative contents of other commensal fecal bacteria were determined by qPCR (n=10).

(F, **G)** In parallel with the experiment described in Figure 5O-S, gross colonic appearance and colon length **(F)** and histological analysis of the distal colon (H&E staining) **(G)** were carried out.

(H) Histograms show the retention time of the L-glutamic acid standard and that of L-glutamic acid detected in the cecal contents from *Clec4n*$^{-/-}$ mice or in the culture supernatant of *L. j.* WXY, in parallel with the experiment described in Figure 6X and 6Y.

(I) Histograms show the retention time of the stachydrine standard and that of stachydrine detected in the culture supernatant of *E. coli*. The bar graph displays the amount of stachydrine detected in the culture supernatants of each indicated bacterial strain using liquid chromatography for targeted metabolomics (n=3 technical replicates/group).

Data in panels (**E**) are pooled from two independent experiments, and in panels (**B**, **C**, **D**, **F**, **G**) are from one of two independent experiments. Statistical analysis: paired Student's t-test (**E**), one-way ANOVA with Tukey's multiple comparisons test (**I**). Data in panels (**I**) are presented as mean ± SD.

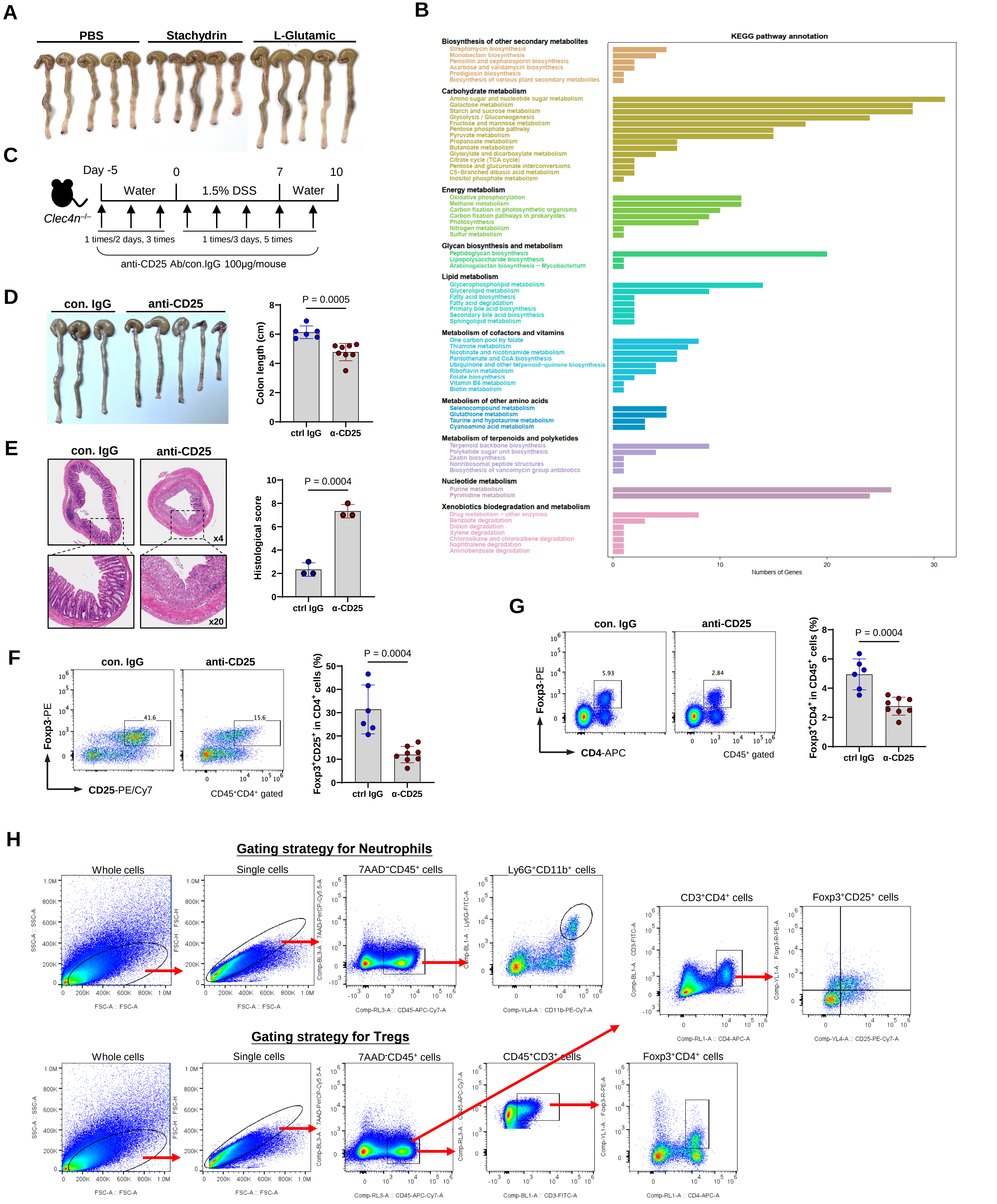

A
PBS
Stachydrin
L-Glutamic
B
KEGG pathway annotation
Biosynthesis of other secondary metabolites
Carbohydrate metabolism
Energy metabolism
Glycan biosynthesis and metabolism
Lipid metabolism
Metabolism of cofactors and vitamins
Metabolism of other amino acids
Metabolism of terpenoids and polyketides
Nucleotide metabolism
Xenobiotics biodegradation and metabolism
Numbers of Genes
C
Day -5
0
7
10
Water
1.5% DSS
Water
Clec4n−/−
1 times/2 days, 3 times
1 times/3 days, 5 times
anti-CD25 Ab/con.IgG 100μg/mouse
D
con. IgG
anti-CD25
P = 0.0005
Colon length (cm)
ctrl IgG
α-CD25
E
con. IgG
anti-CD25
x4
x20
P = 0.0004
Histological score
ctrl IgG
α-CD25
F
con. IgG
anti-CD25
41.6
15.6
Foxp3-PE
CD25-PE/Cy7
CD45+CD4+ gated
P = 0.0004
Foxp3+CD25+ in CD4+ cells (%)
ctrl IgG
α-CD25
G
con. IgG
anti-CD25
5.93
2.84
Foxp3-PE
CD4-APC
CD45+ gated
P = 0.0004
Foxp3+CD4+ in CD45+ cells (%)
ctrl IgG
α-CD25
H
Gating strategy for Neutrophils
Whole cells
Single cells
7AAD−CD45+ cells
Ly6G+CD11b+ cells
CD3+CD4+ cells
Foxp3+CD25+ cells
Gating strategy for Tregs
Whole cells
Single cells
7AAD-CD45+ cells
CD45+CD3+ cells
Foxp3+CD4+ cells

Supplementary Figure 8. Glutamic acid secreted by *Lactobacillus johnsonii* WXY inhibits DSS-colitis by promoting Treg cell expansion

(A) Gross observation of the colon was performed in parallel with the experiment described in Figure 6A-E.

(B) Other physiological metabolic functions of *L. j*. WXY were exhibited from complete genome sequencing analysis using KEGG annotation, in parallel with the experiment described in Figure 6P.

(C-G) The experimental protocol is shown in **(C)** in parallel with the experiment described in Figure 6T and 6U. On day 10 post-DSS treatment, gross observation of the colon tissues, colon length measurement **(D)**, and histological analysis of the distal colon (H&E staining) **(E)** were performed. The proportions of Foxp3^{+}CD25^{+} in CD4^{+} T cells **(F)** and Foxp3^{+}CD4^{+} in CD45^{+} cells **(G)** were examined by FACS (**D**, **F**, **G**, control IgG n=6, anti-CD25 n=8, **E**, n=3/group).

(H) Gating strategy for cLP neutrophils and Tregs of mice by flow cytometry. Whole cells were firstly gated for the cluster located on the left bottom of FSC-SSC dot panel (region from 100K~1M of FSC axis and 0K~900K of SSC axis) with the elliptic gate, and then was gated on FSC-H × FSC-A to get the single cells. live cells were then selected by gating on 7AAD-negative cells with rectangle gate. To analyze neutrophils within the leukocytes, CD45^{+} population was further gated for showing CD11b^{+}Ly6G^{+} cell cluster. To analyze Treg cells, CD45^{+} population was further gated for showing Foxp3^{+}CD4^{+} cell cluster in CD3ε^{+} T cell population or CD25^{+}Foxp3^{+} cell cluster in CD3ε^{+}CD4^{+} T cell population.

Data in panels (**D**, **F**, **G**) are pooled from two independent experiments, and in panel (**A**) are from one of two independent experiments. Statistical analysis: two-tailed unpaired Student's t-test (**D-G**). Data in panels (**D-G**) are presented as mean ± SD.

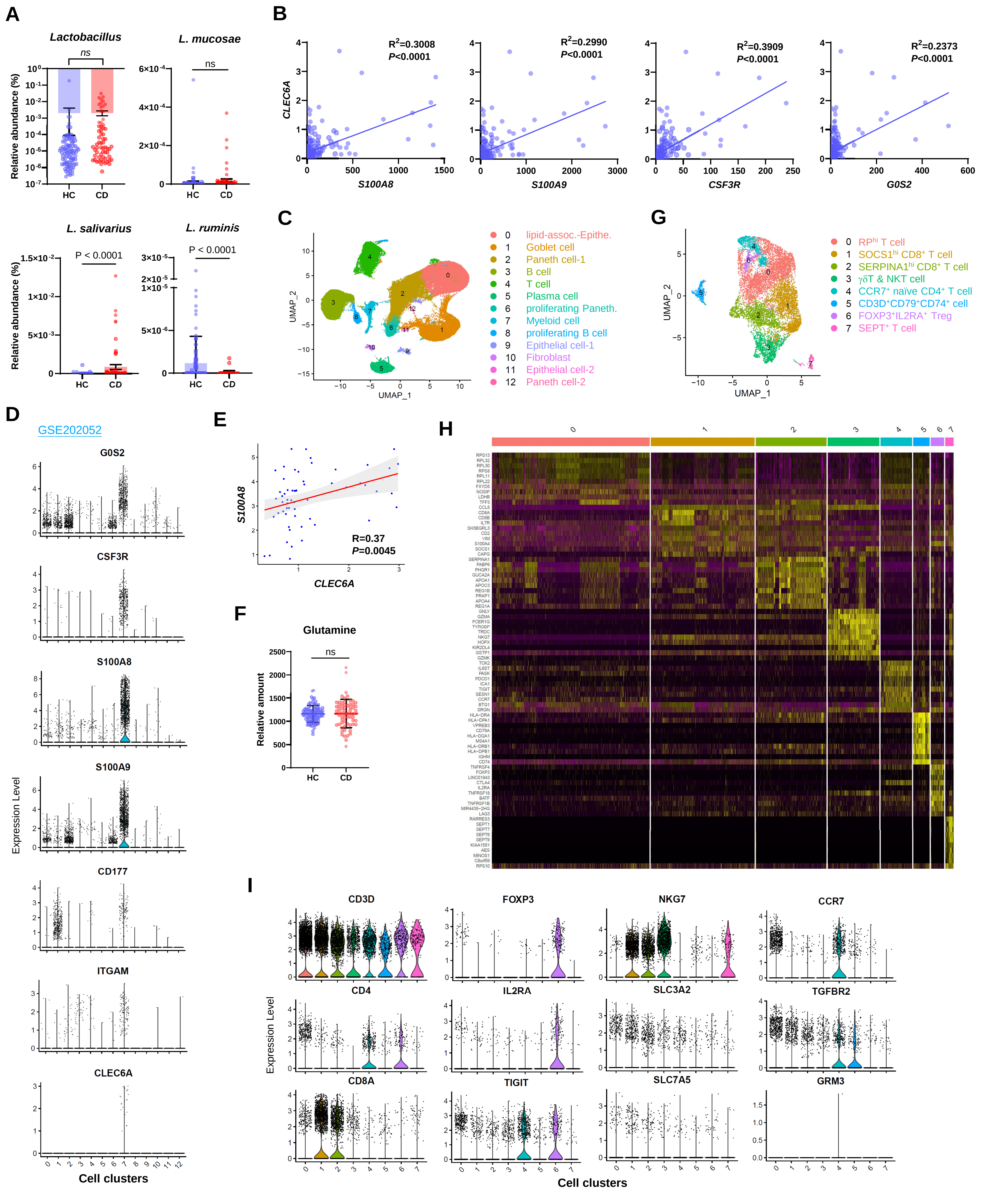
A
Lactobacillus
L. mucosae
L. salivarius
L. ruminis
Relative abundance (%)
ns
P < 0.0001
HC
CD
B
CLEC6A
R2=0.3008 P<0.0001
R2=0.2990 P<0.0001
R2=0.3909 P<0.0001
R2=0.2373 P<0.0001
S100A8
S100A9
CSF3R
G0S2
C
UMAP_1
UMAP_2
0 lipid-assoc.-Epithe.
1 Goblet cell
2 Paneth cell-1
3 B cell
4 T cell
5 Plasma cell
6 proliferating Paneth.
7 Myeloid cell
8 proliferating B cell
9 Epithelial cell-1
10 Fibroblast
11 Epithelial cell-2
12 Paneth cell-2
G
0 RPhi T cell
1 SOCS1hi CD8+ T cell
2 SERPINA1hi CD8+ T cell
3 γδT & NKT cell
4 CCR7+ naïve CD4+ T cell
5 CD3D+CD79+CD74+ cell
6 FOXP3+IL2RA+ Treg
7 SEPT+ T cell
D
GSE202052
G0S2
CSF3R
S100A8
S100A9
CD177
ITGAM
CLEC6A
Expression Level
Cell clusters
E
S100A8
CLEC6A
R=0.37
P=0.0045
F
Glutamine
Relative amount
ns
HC
CD
H
I
CD3D
FOXP3
NKG7
CCR7
CD4
IL2RA
SLC3A2
TGFBR2
CD8A
TIGIT
SLC7A5
GRM3
Expression Level
Cell clusters

Supplementary Figure 9. Constitutive expression of Glutamic acid receptors on colonic T cells

(A) The relative abundance of the whole *Lactobacilli* and indicated *Lactobacillus* species in fecal bacteria from CD patients and healthy individuals was determined by 16S rDNA sequencing analysis (HC, n=95; CD, n=65).

(B) Point plots show the correlation between the expression levels of *CLEC6A* and *S100A8*, *S100A9*, *CSF3R*, or *G0S2* in the colon tissues from all pooled human samples in the public RNA-seq dataset (GSE117993).

(C-E) A public scRNA-seq dataset of colonic cells from IBD patients (GSE202052) was reanalyzed. A UMAP plot identifies cell type clusters in inflammatory colon tissues **(C)**, violin plots illustrate the expression of indicated genes in each identified cell cluster **(D)**, and a point plot shows the correlation between the expression levels of *S100A8* and *CLEC6A* in *CLEC6A*$^{+}$*S100A8*$^{+}$ cells **(E)** .

(F) In parallel with the experiment described in Figure 7S, the relative amount of glutamine in the feces harvested from CD patients and healthy individuals was assessed by liquid chromatography for untargeted metabolomics (HC, n=80; CD, n=65).

(G-I) Another scRNA-seq public dataset of colonic cells from IBD patients (GSE202052) was reanalyzed. A UMAP plot displays each T cell cluster (subsets) in the colon of IBD patients **(G)**, a heatmap shows the gene profiles of identified T cell subsets **(H)**, and violin plots illustrate the expression of indicated T-cell associated genes and glutamic acid receptors SLC7A5, SLC3A2, and GRM3 in each identified cell cluster **(I)**.

Data in panel (**A**) are presented as mean ± SEM, and in (**F**) are presented as mean ± SD. Statistical analysis: two-tailed Mann-Whitney test (**A**), two-tailed unpaired Student’s t-test (**F**).

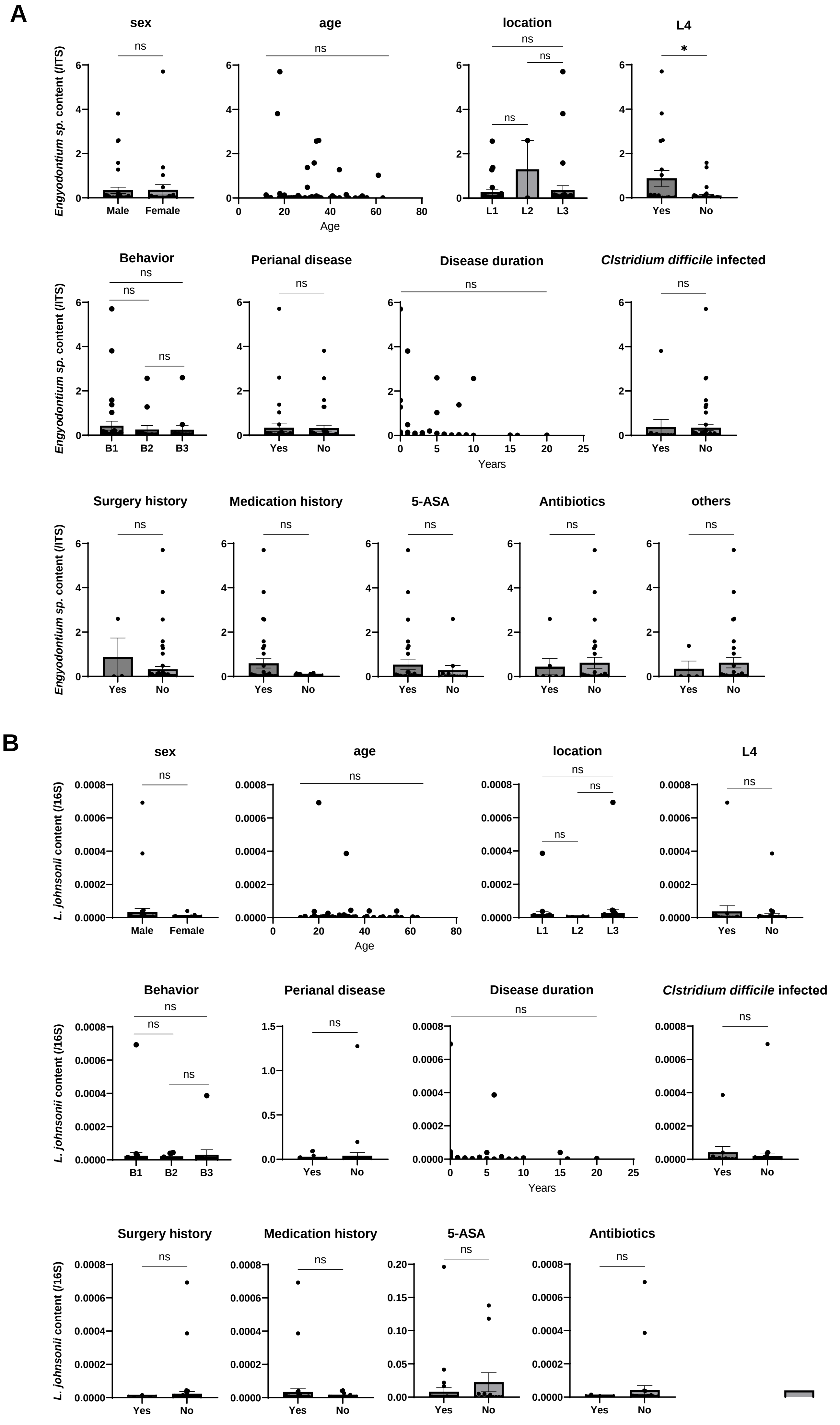
A
sex
age
location
L4
Engyodontium sp. content (/ITS)
Male
Female
Age
L1
L2
L3
Yes
No
ns
*
Behavior
Perianal disease
Disease duration
Clstridium difficile infected
B1
B2
B3
Years
Surgery history
Medication history
5-ASA
Antibiotics
others
B
L. johnsonii content (/16S)

Supplementary Figure 10. The abundances of *Engyodontium* sp. and *L. johnsonii* were not associated with clinical parameters of CD patients.

The potential correlation between the content of *Engyodontium* sp. **(A)** or *L. johnsonii* **(B)** and sex, age, disease location (L1: ileal, L2: colonic, L3: ileocolonic, L4: upper-isolated gastrointestinal), disease behavior (B1: non-stricturing and non-penetrating, B2: stricturing, B3: penetrating), disease duration, *Clostridium difficile* infection, or medication use (surgery history, 5-aminosalicylates (ASA), antibiotics and others) in CD patients (n=63). For categorical variables (e.g., disease location, medication use), comparisons were made using Mann-Whitney U or Kruskal-Wallis tests; for continuous variables (e.g., disease duration), Spearman correlation analysis was employed. Details in clinical parameters of individuals are listed in Supplementary Table 2.

Supplementary Table 1. Metabolite selection list (associated with Figure 5V, 5W)

Name	Formula	control	*E.coil*	*L.j.*WXY	*L.*mix	*L.j.*WXY/control	*L.*mix/control	*L.j.*WXY/*E.coli*	*L.*mix/*E.coli*
2-Dimethylamino-5,6-dimethyl-4-hydroxypyrimidine	C8 H13 N3 O	253469372	1175946072	3261509068	3459282889	12.9	13.6	2.8	2.9
heptaminol	C8 H19 N O	28757875	27700481	357889526	131842686	12.4	4.6	12.9	4.8
(2E)-N-(4-Aminobutyl)-3-(2,4-dihydroxy-3-methoxyphenyl)acrylamide	C14 H20 N2 O4	21666963	50476733	194883658	56619281	9.0	2.6	3.9	1.1
L-Glutamic acid	***C5 H9 N O4***	***72089773***	***56804073***	***143125045***	***120048310***	***2.0***	***1.7***	***2.5***	***2.1***
Stachydrine	*C7 H13 N O2*	*15697395*	*5439419*	*124803756*	*16881595*	*8.0*	*1.1*	*22.9*	*3.1*
4-Imidazolemethanol	C4 H6 N2 O	26179223	15653941	57388260	30380565	2.2	1.2	3.7	1.9
6-Methyl-2-pyridinemethanol	C7 H9 N O	16954306	11142222	51650154	31312847	3.0	1.8	4.6	2.8
7,8-Dihydroneopterin	C9 H13 N5 O4	565651	8012504	32538846	24311107	57.5	43.0	4.1	3.0
Acetylcholine	C7 H15 N O2	15270799	10604730	30966336	32835640	2.0	2.2	2.9	3.1
Zearalanone	C18 H24 O5	9998116	12378296	30549159	30097020	3.1	3.0	2.5	2.4
Histamine	C5 H9 N3	1942021	9248372	25150695	25966999	13.0	13.4	2.7	2.8
7-Methyladenine	C6 H7 N5	8981524	10911963	21584737	33604089	2.4	3.7	2.0	3.1
[(4Z,6E,10Z,13Z)-1-Carboxy-4,6,10,13-nonadecatetraen-8-yl]dioxidanyl	C20 H31 O4	308084	78682	21406764	417001	69.5	1.4	272.1	5.3
3-Î±-Mycarosylerythronolide B	C28 H50 O10	9603667	62351	20453351	17130117	2.1	1.8	328.0	274.7
n-Hexanamide	C6 H13 N O	9115584	7347416	18664506	9614562	2.0	1.1	2.5	1.3
Asparaginyl-4-hydroxyproline	C9 H15 N3 O5	1726198	7279201	16900300	7152294	9.8	4.1	2.3	1.0
3-[2-[(E)-[3-(2-carboxyethyl)-5-[(4-ethyl-3-methyl-5-oxo-pyrrolidin-2-yl)methyl]-4-methyl-pyrrol-2-ylidene]methyl]-5-[(3-ethyl-4-methyl-5-oxo-pyrrolidin-2-yl)methyl]-4-methyl-1H-pyrrol-3-yl]propanoic acid	C33 H46 N4 O6	6704247	64978	13367408	9803907	2.0	1.5	205.7	150.9
Oleamide	C18 H35 N O	5832914	1494861	11853679	7142491	2.0	1.2	7.9	4.8
1-(Methylnitrosoamino)-4-(3-pyridinyl)-1,4-butanediol	C10 H15 N3 O3	669519	3588897	10465084	2179408	15.6	3.3	2.9	0.6
Levetiracetam	C8 H14 N2 O2	3233985	1857144	7256745	4025374	2.2	1.2	3.9	2.2
4-Methylhistamine	C6 H11 N3	824202	200801	4826235	4308272	5.9	5.2	24.0	21.5
2,6-Diamino-4-hexenoic acid	C6 H12 N2 O2	1114791	1353556	2780151	12366240	2.5	11.1	2.1	9.1

Supplementary Table 2. Disease parameters in CD patients (associated with Supplementary Figure 10)

ID	sex	age	BMI	diff	Location	L4	Behavior	Perianal_disease	duration	no_medicine	5_ASA	antibiotics	others	surgery	*Engyodontium*	*L. johnsonii*
CD0065	1	34	24.8015873	0	3	0	2	1	0	1	0	0	0	0	0.09496693	4.4448E-05
CD0077	0	35	21.7784352	0	1	0	3	1	3	0	1	0	0	0	0.0413738	3.3277E-06
CD0087	1	18	30.7787011	0	1	0	1	0	4	0	1	0	0	0	0.19601532	1.1737E-05
CD0088	1	32		0	3	0	1	1	9	0	1	0	1	0	0.02169725	1.3169E-08
CD0091	0	48	16.2330728	1	3	0	3	0	1	0	1	0	0	0	0.00107279	7.6262E-11
CD0136	1	25	21.2802768	1	3	0	3	0	3	0	1	0	0	0	0.01657267	3.4927E-08
CD0153	1	44	18.3391004	0	1	1	2	0	0	0	1	0	0	0	1.27605649	2.6634E-08
CD0187	1	22	15.916955	1	3	0	2	0	0	1	0	0	0	0	0.0047123	4.8581E-06
CD0190	1	19	14.8729043	0	1	1	1	0	1	1	0	0	0	0	0.00360337	1.9476E-06
CD0207	1	31	16.8296485	0	1	0	3	0	0	1	0	0	0	0	0.0050438	2.4372E-06
CD0229	0	33	26.1426885	0	3	0	2	0	0	0	1	0	0	0	0.03711038	1.5735E-07
CD0255	0	12	12.2255367	0	3	1	1	0	1	1	0	0	0	0	0.13795237	2.893E-08
CD0266	1	54	27.6816609	0	1	0	1	1	20	0	1	0	0	0	0.00724787	3.7868E-06
CD0267	1	20	16.8061342	0	3	0	1	0	3	1	0	0	0	0	0.11802933	1.6722E-09
CD0270	0	54	23.2334562	0	3	0	2	1	5	0	1	0	0	0	0.09037015	3.9849E-05
CD0277	1	33	23.4567901	0	3	0	3	0	0	1	0	0	0	0	0.01304514	1.2827E-06
CD0280	0	19	15.8221589	0	1	0	2	1	0	0	1	0	0	0	0.00256898	7.6475E-08
CD0283	1	51	22.790329	0	1	1	2	1	0	1	0	0	0	0	0.00089242	4.8478E-08
CD0328	0	29	21.230572	1	3	0	1	0	0	1	0	0	0	0	0.00762535	3.2205E-06
CD0330	0	48	17.3333333	0	3	0	2	1	1	0	1	0	0	0	0.00158223	4.6134E-06
CD0331	0	41	24.7767953	0	1	1	1	1	2	1	0	0	0	0	0.09722083	5.9768E-07
CD0371	1	36	26.0802469	0	1	0	1	1	0	1	0	0	0	0	0.00107047	4.3904E-06
CD0374	0	18	21.1884318	0	3	0	1	0	0	1	0	0	0	0	0.02126909	1.3503E-10
CD0517	1	44	17.6005226	1		1	2	1	0	1	0	0	0	0	0.00917732	2.6606E-07
CD0529	1	22	28.4055044	0	3	0	1	0	3	1	0	0	0	0	0.00492275	2.3884E-08
CD0541	0	23	15.6734694	0	3	1	3	0	0	0	0	1	0	0	0.00590011	6.5448E-06
CD0557	0	51	16.025641	0	3	0	1	0	0	1	0	0	0	0	0.00137608	7.5786E-07
CD0563	0	31	16.0230732	1	3	0	2	1	0	1	0	0	0	0	0.01730141	1.6242E-05
CD0575	0	61	25.4374808	0		1	1	1	5	0	1	0	0	0	1.02809953	3.4837E-06
CD0624	1	24	18.6997809	0	3	1	1	1	0	1	0	0	0	0	0.0122366	2.6105E-05
CD0627	1	26	23.3217993	1	3	1	1	0	0	1	0	0	0	0	0.11269134	7.3595E-07
CD0631	1	18	16.1616162	0	1	0	1	0	0	0	1	0	0	0	0.05077775	3.679E-05
CD0632	1	33	20.6192537	0	3	0	1	0	0	0	1	0	0	0	1.58158933	6.0421E-06
CD0660	1	35	21.789966	0	3	1	1	1	8	1	0	0	0	0	0.02682979	4.1495E-07
CD0665	1	34	19.53125	0	1	1	2	0	10	0	1	0	0	0	2.56973904	7.9228E-09
CD0676	1	53	23.1206236	0	1	0	1	1	1	1	0	0	0	0	0.00418937	3.9107E-08
CD0679	1	42	17.3010381	1	3	0	2	0	15	1	0	0	0	0	0.01084388	4.0542E-05
CD0708	1	33	18.4964989	0	3	0	3	0	0	1	0	0	0	0	0.00102912	5.8104E-08
CD0709	1	29	21.2962963	0	1	1	1	1	7	0	0	1	0	1	0.01527035	1.4668E-05
CD0763	0	56	19.5555556	0	1	0	3	1	3	1	0	0	0	0	0.00735063	1.416E-07
CD0772	1	14	29.4117647	1	3	0	1	0	2	0	1	0	0	0	0.01725568	7.212E-06
CD0785	1	35	14.8788927	0	2	1	3	1	5	0	0	1	0	1	2.59593983	5.8198E-08
CD0818	0	30	20.5078125	0	1	0	1	1	8	0	1	0	1	0	1.37585014	6.8811E-08
CD0820	1	17	22.4913495	1	3	1	1	0	1	0	1	0	0	0	3.80902161	8.4514E-07
CD0846	0	18	20.4294184	0	3	1	1	1	0	0	1	0	0	0	5.70250845	2.1077E-07
CD0859	0	30	15.2352608	0	1	0	3	1	1	0	0	1	0	0	0.48379601	1.6864E-06
CD0866	1	14	18.9020381	0	1	1	1	1	1	0	1	0	0	0	0	1.6616E-07
CD0871	0	48	20.703125	0	1	0	2	0	3	0	1	0	0	0	0	1.8842E-07
CD0883	1	40	16.4153073	0		1	2	0	0	0	0	1	0	0	0	2.9758E-07
CD0894	1	41	21.4691505	0	3	0	3	1	0	0	1	0	0	0	0	7.3846E-07
CD0897	1	12	16.9921875	0	1	0	1	0	0	1	0	0	0	0	0	3.1859E-07
CD0898	0	32	18.4265342	0	1	0	3	1	1	0	1	0	0	0	0	9.1757E-06
CD0908	0	29	19.4674013	0	3	0	1	1	0	0	0	1	1	0	0	1.6337E-07
CD0910	0	14	11.1111111	0	3	0	1	1	1	1	0	0	0	0	0	3.1299E-06
CD0941	1	41	30.1038062	0	3	0	2	0	10	0	1	0	0	0	0	6.7791E-06
CD0949	0	63	21.484375	0	1	0	1	1	16	0	1	0	0	0	0	6.7348E-07
CD0952	0	27	20.0288415	0	3	0	2	1	6	0	1	0	1	0	0	4.0664E-07
CD0966	0	21	17.8980229	0	3	0	1	0	0	0	0	1	0	0	0	3.2638E-06
CD0980	1	26	22.0968079	0	3	0	1	0	2	0	1	0	0	0	0	4.0518E-06
CD0991	1	24	16.7311124	0		1	1	1	0	1	0	0	0	1	0	8.2384E-07
CD662	1	32	13.9079409	1	1	0	3	0	6	0	1	0	0	0	0.05937787	0.00038579
CD663	1	20	18.3654729	0	3	1	1	0	0	0	1	0	0	0	0.13479412	0.00069241
TB0021	1	47	20.8652666	0	3	0	1	1	0	1	0	0	0	0	0.15288995	1.1776E-06
HC				0											0.00412123	0.00019545
HC				0											0.00877708	0.00018102
HC				0											0.0166053	0.00035831
HC				0											0.00045068	0.00020729
HC				0											0.00012519	0.00065798
HC				0											0.00154932	6.1469E-06
HC				0											1.2728E-06	0.00011462
HC				0											0.11283461	5.9699E-05
HC				0											0.05420992	0.00129207
HC				0											0.00231172	0.00048718
HC				0											0.00245397	0.00046299
HC				0											0.13191727	0.00017162
HC				0											0.02457826	5.533E-05
HC				0											0.00200933	6.8496E-05
HC				0											0.00372163	0.00019289
HC				0											0.00992965	0.00014496
HC				0											0.01076237	0.00044139
HC				0											0.00295856	0.00133293
HC				0											0.00885267	0.00022354
HC				0											0.01073755	0.00124587
HC				0											0.01287112	0.00141947
HC				0											0.00422883	0.00075897
HC				0											0.01369175	0.00019261
HC				0											0.01261375	0.00041471
HC				0											0.0072039	0.00064959
HC				0											0.00078058	0.00408422
HC				0											0.0002776	7.2571E-05
HC				0											0.12955218	0.00055783
HC				0											0.01490699	0.00162734
HC				0											0.01057988	0.00078599
HC				0											0.00033371	0.00070853
HC				0											0.34552539	0.00039627
HC				0											0.0121554	0.00601424
HC				0											0.00235014	0.0002798
HC				0											0.00488016	0.00044835
HC				0											0.00071654	0.00011198
HC				0											0.01803319	0.00014069
HC				0											0.08284569	0.00012851
HC				0											0.01120457	1.3085E-05
HC				0											0.00026386	0.00031566
HC				0											0.00090117	0.00023694
HC				0											0.00573435	0.00029758
HC				0											0.00444438	0.00411152
HC				0											0.00788618	2.6356E-05
HC				0											0.00754425	1.504E-05
HC				0											0.01564621	2.491E-05
HC				0											0.01004088	1.0116E-05
HC				0											0.01693632	1.1484E-05
HC				0											0	1.3476E-05
HC				0											0	1.0978E-05
HC				0											0	1.322E-05
HC				0											0	1.9519E-05
HC				0											0	7.1597E-05
HC				0											0	2.3947E-06
HC				0											0	2.491E-05
HC				0											0	1.1484E-05
HC				0											0	3.0397E-06
HC				0											0	2.491E-05
HC				0											0	1.3434E-06
HC				0											0	2.0111E-06
HC				0											0	1.4494E-06
HC				0											0	2.4906E-05

Supplementary Table 3. Sequences and Primers

For qPCR (mouse)

**Gapdh**	mouse	F	TTCACCACCATGGAGAAGGC
		R	GGCATGGACTGTGGTCATGA
**Il6**	mouse	F	GAGGATACCACTCCCAACAGACC
		R	AAGTGCATCATCGTTGTTCATACA
**Tnf**	mouse	F	GAGGATACCACTCCCAACAGACC
		R	AAGTGCATCATCGTTGTTCATACA
**S100a9**	mouse	F	GAGGATACCACTCCCAACAGACC
		R	AAGTGCATCATCGTTGTTCATACA
**S100a8**	mouse	F	GAGGATACCACTCCCAACAGACC
		R	AAGTGCATCATCGTTGTTCATACA
**Cxcl2**	mouse	F	CCAACCACCAGGCTACAGG
		R	GCGTCACACTCAAGCTCTG
**Il17a**	mouse	F	TCCCACGAAATCCAGGATGC
		R	GGATGTTCAGGTTGACCATCAC
**Ifng**	mouse	F	GAACTGGCAAAAGGATGGTGA
		R	TGTGGGTTGTTGACCTCAAAC
**Il10**	mouse	F	GCTCTTACTGACTGGCATGAG
		R	CGCAGCTCTAGGAGCATGTG
**Tgfb1**	mouse	F	TGACGTCACTGGAGTTGTACGG
		R	GGTTCATGTCATGGATGGTGC
**Foxp3**	mouse	F	CCCATCCCCAGGAGTCTTG
		R	ACCATGACTAGGGGCACTGTA
**Il2ra**	mouse	F	GGGAAAACGGGGTGGACTC
		R	CTGTGGTGGTTATGGGGCAG
**Il2**	mouse	F	TGAGCAGGATGGAGAATTACAGG
		R	GTCCAAGTTCATCTTCTAGGCAC
**Defb1**	mouse	F	AGGTGTTGGCATTCTCACAAG
		R	GCTTATCTGGTTTACAGGTTCCC
**Defb2**	mouse	F	TATGCTGCCTCCTTTTCTCA
		R	GACTTCCATGTGCTTCCTTC
**Lyz1**	mouse	F	GAGACCGAAGCACCGACTATG
		R	CGGTTTTGACATTGTGTTCGC
**Lyz2**	mouse	F	ATGGAATGGCTGGCTACTATGG
		R	ACCAGTATCGGCTATTGATCTGA
**Defa1**	mouse	F	TCAAGAGGCTGCAAAGGAAGAGAAC
		R	TGGTCTCCATGTTCAGCGACAGC
**Reg3b**	mouse	F	CTCTCCTGCCTGATGCTCTT
		R	GTAGGAGCCATAAGCCTGGG
**Reg3g**	mouse	F	TCAGGTGCAAGGTGAAGTTG
		R	GGCCACTGTTACCACTGCTT
**Zo1**	mouse	F	GCCGCTAAGAGCACAGCAA
		R	GCCCTCCTTTTAACACATCAGA
**Cldn7**	mouse	F	GGCCTGATAGCGAGCACTG
		R	GTGACGCACTCCATCCAGA
**Cldn4**	mouse	F	GTCCTGGGAATCTCCTTGGC
		R	TCTGTGCCGTGACGATGTTG
**Tjp**	mouse	F	GCCGCTAAGAGCACAGCAA
		R	TCCCCACTCTGAAAATGAGGA
**Ocln**	mouse	F	TTGAAAGTCCACCTCCTTACAGA
		R	CCGGATAAAAAGAGTACGCTGG
**Muc2**	mouse	F	GCTGACGAGTGGTTGGTGAATG
		R	GATGAGGTGGCAGACAGGAGAC
**Ctla4**	mouse	F	GCTTCCTAGATTACCCCTTCTGC
		R	CGGGCATGGTTCTGGATCA
**Clec5a**	mouse	F	TCGGGGCTTATCGTAGTAGTG
		R	TGTAGGCATGGTACTTTCGTCAT
**Il2ra**	mouse	F	GGGAAAACGGGGTGGACTC
		R	CTGTGGTGGTTATGGGGCAG
**Il1b**	mouse	F	CAACCAACAAGTGATATTCTCCATG
		R	GATCCACACTCTCCAGCTGCA

For qPCR (human)

GAPDH	human	F	TTCACCACCATGGAGAAGGC
		R	GGCATGGACTGTGGTCATGA
S100A8	human	F	ATGCCGTCTACAGGGATGAC
		R	ACTGAGGACACTCGGTCTCTA
S100A9	human	F	GGTCATAGAACACATCATGGAGG
		R	GGCCTGGCTTATGGTGGTG
CXCL1	human	F	CGCTACAGCGACGTGAAGAA
		R	GTTCCAGGCGTTGTACCAC
CXCL2	human	F	CCGAAGTCATAGCCACACTCA
		R	TGGATTTGCCATTTTTCAGCATCT

For qPCR (bacteria & fungi)

**16S (8F&R357)**	bacteria	F	AGAGTTTGATCMTGGCTCAG
		R	CTGCTGCCTYCCGTA
**Bacteroidetes**	bacteria	F	CRAACAGGATTAGATACCCT
		R	GGTAAGGTTCCTCGCGTAT
**Firmicutes**	bacteria	F	TGAAACTYAAAGGAATTGACG
		R	ACCATGCACCACCTGTC
**Actinobacteria**	bacteria	F	TACGGCCGCAAGGCTA
		R	TCRTCCCCACCTTCCTCCG
**aProteobacteria**	bacteria	F	CIAGTGTAGAGGTGAAATT
		R	CCCCGTCAATTCCTTTGAGTT
**gProteobacteria**	bacteria	F	CGTAAGGGCCATGATG
		R	CTGCTGCCTYCCGTA
**bProteobacteria**	bacteria	F	AACGCGAAAAACCTTACCTACC
		R	TGCCCTTTCGTAGCAACTAGTG
**eProteobacteria**	bacteria	F	TAGGCTTGACATTGATAGAATC
		R	CTTACGAAGGCAGTCTCCTTA
**Cyanobacteria**	bacteria	F	GGGGAATYTTCCGCAATGGG
		R	GACTACTGGGGTATCTAATCCCATT
**Tenericutes**	bacteria	F	ATGTGTAGCGGTAAAATGCGTAA
		R	CMTACTTGCGTACGTACTACT
**Verrucomicrobia**	bacteria	F	TCAKGTCAGTATGGCCCTTAT
		R	CAGTTTTYAGGATTTCCTCCGCC
**Deferribacteres**	bacteria	F	CTATTTCCAGTTGCTAACGG
		R	GAGHTGCTTCCCTCTGATTATG
**Lactobacilli**	bacteria	F	TGGAAACAGRTGCTAATACCG
		R	GTCCATTGTGGAAGATTCCC
**Lactobacillus johnsonii**	bacteria	F	TCGAGCGAGCTTGCCTAGATGA
		R	TCCGGACAACGCTTGCCACC
**glsA**	bacteria	F	TGGTGAAGTAGGTGAAGCAGG
		R	AATGCGTTTGTTGTCCCATG
**ITS1-2**	fungi	F	CTTGGTCATTTAGAGGAAGTAA
		R	GCTGCGTTCTTCATCGATGC
**Engyodontium sp. SM14-25-6-1**	fungi	F	GGAGGGATCATTACCGAGTTTACA
		R	TCCGTTGTTGAAAGTTTTGATTCATTT
**Ascomycota**	fungi	F	GAATTGCAGMMWTCMGTGAATC
		R	GCCTGTYTGAGCGTCRTTTC
**Basidiomycota**	fungi	F	CGAATCTTTGAACGCAMCTTG
		R	GCCTGTTTGAGTATCATGA
**Zygomycota**	fungi	F	TTCAAAGAGTCAGGTTGTTTGG
		R	CAGTCTGGCTCCAAACGGTTC
**Chytridiomycota**	fungi	F	GTAACGGAGAATTAGGGTTCG
		R	CCTCCGCTTATTGATATGCT
**Glomeromycota**	fungi	F	GGAGATGCTGGAGAATTTG
		R	TGAAGGTGAGTCTGAAGAAG
**Rozellomycota**	fungi	F	CCTACGGGAGGCAGCAGT
		R	GACTACCAGGGTATCTAATCCTGTT

For mouse generating & typing

Clec4n KO CRISPR-Cpf1 gene.	target sequence	Exon 1	TTTCCCATGTCTTTAAATCAGTGGAGAG
		Exon 4	TTTCTACCAAGGAGAACTTCTGGAGCAC
	typing primers	F	CCACATTAGGAACTGAGAAAG
		R	ACAAGAAAGCACTACCTGCTCC
Card9 KO CRISPR-Cpf1 gene.	target sequence	Exon 2	TTTCCGGTATAACTGAGGGTAGTAGAGC
	typing primers	F	TTGACTCTGTCTTCCACCTGAA
		R	TCACACTTAGTCACTCCAACGC

Supplementary Table 4. Antibodies used

Antibodies	Clone	Cat#	Source
APC/Cyanine7 anti-mouse CD45	30-F11	103116	BioLegend
PE/Cyanine7 anti-mouse/human CD11b	M1/70	101216	BioLegend
FITC anti-mouse Ly-6G	1A8	127606	BioLegend
PE anti-mouse Ly-6C	HK1.4	128007	BioLegend
Pacific Blue™ anti-mouse CD11c	N418	117322	BioLegend
APC anti-mouse F4/80	BM8	123115	BioLegend
FITC anti-mouse CD3ε	145-2C11	100305	BioLegend
APC anti-mouse CD4	GK1.5	100411	BioLegend
PE anti-mouse CD8a	53-6.7	100707	BioLegend
PE/Cyanine7 anti-mouse CD25	PC61	102015	BioLegend
APC anti-mouse I-A/I-E	M5/114.15.2	107614	BioLegend
Pacific Blue anti-mouse IL-17A	TC11-18H10.1	506918	BioLegend
FITC anti-mouse IFN-γ	XMG1.2	505806	BioLegend
FITC anti-mouse IL-2	JES6-5H4	503805	BioLegend
PE anti-mouse FOXP3	MF-14	126403	BioLegend
Brilliant Violet 510 anti-human CD45	2D1	368526	BioLegend
FITC anti-human CD4	RPA-T4	300506	BioLegend
PE anti-human CD25	BC96	302606	BioLegend
PE/Dazzle 594 anti-human FOXP3	206D	320126	BioLegend
Rabbit polyclonal antibody to CLEC6A	Polyclonal	DF10182	Affinity Biosciences
Rabbit polyclonal antibody to S100A8	Polyclonal	DF6556	Affinity Biosciences
Rabbit polyclonal antibody to S100A9	Polyclonal	DF7596	Affinity Biosciences
HRP- labeled goat anti-rabbit IgG	Polyclonal	GB23303	ServiceBio
Biotin anti-mouse/human CD11b	M1/70	101203	BioLegend
Biotin anti-mouse CD11c	N418	117303	BioLegend
Biotin anti-mouse I-A/I-E	M5/114.15.2	107603	BioLegend
Biotin anti-mouse CD19	6D5	115504	BioLegend
Biotin anti-mouse TCR γ/δ	GL3	118103	BioLegend
Biotin anti-mouse NK-1.1	PK136	108703	BioLegend
Biotin anti-mouse CD8a	53-6.7	100704	BioLegend
Ultra-LEAF™ Purified anti-mouse IL-4	11B11	504135	BioLegend
Ultra-LEAF™ Purified anti-mouse IFN-γ	XMG1.2	505847	BioLegend
InVivoMAb anti-mouse CD25 (IL-2Rα)	PC-61.5.3	BE0012	Bio X Cell
InVivoMAb rat IgG1 isotype control	HRPN-CP167	BE0088	Bio X Cell
Goat Anti-Human IgG Fc (Biotin)	Polyclonal	Ab97223	Abcam
Anti-CXCL2 antibody	EPR28746-89	ab317569	Abcam
Anti-Dectin-2 antibody	D2.11E4	GTX41453	GeneTex
Ly6g Rabbit Polyclonal Antibody	Polyclonal	0809-11	HUABIO
